# Antibody-mediated broad sarbecovirus neutralization through ACE2 molecular mimicry

**DOI:** 10.1126/science.abm8143

**Published:** 2022-01-06

**Authors:** Young-Jun Park, Anna De Marco, Tyler N. Starr, Zhuoming Liu, Dora Pinto, Alexandra C. Walls, Fabrizia Zatta, Samantha K. Zepeda, John E. Bowen, Kaitlin R. Sprouse, Anshu Joshi, Martina Giurdanella, Barbara Guarino, Julia Noack, Rana Abdelnabi, Shi-Yan Caroline Foo, Laura E. Rosen, Florian A. Lempp, Fabio Benigni, Gyorgy Snell, Johan Neyts, Sean P. J. Whelan, Herbert W. Virgin, Jesse D. Bloom, Davide Corti, Matteo Samuele Pizzuto, David Veesler

**Affiliations:** 1 Department of Biochemistry, University of Washington, Seattle, WA 98195, USA.; 2 Howard Hughes Medical Institute, University of Washington, Seattle, WA 98195, USA.; 3 Humabs Biomed SA, a subsidiary of Vir Biotechnology, 6500 Bellinzona, Switzerland.; 4 Basic Sciences Division, Fred Hutchinson Cancer Research Center, Seattle, WA 98109, USA.; 5 Department of Molecular Microbiology, Washington University School of Medicine, St. Louis, MO 63110, USA.; 6 Vir Biotechnology, San Francisco, CA 94158, USA.; 7 Laboratory of Virology and Chemotherapy, Rega Institute for Medical Research, KU Leuven, 3000 Leuven, Belgium.; 8 Department of Pathology and Immunology, Washington University School of Medicine, St. Louis, MO 63110, USA.; 9 Department of Internal Medicine, University of Texas Southwestern Medical Center, Dallas, TX 75390, USA.

## Abstract

Understanding broadly neutralizing sarbecovirus antibody responses is key to developing countermeasures against severe acute respiratory syndrome coronavirus 2 (SARS-CoV-2) variants and future zoonotic sarbecoviruses. We describe the isolation and characterization of a human monoclonal antibody, designated S2K146, that broadly neutralizes viruses belonging to SARS-CoV– and SARS-CoV-2–related sarbecovirus clades, which use angiotensin-converting enzyme 2 (ACE2) as an entry receptor. Structural and functional studies show that most of the virus residues that directly bind S2K146 are also involved in binding to ACE2. This allows the antibody to potently inhibit receptor attachment. S2K146 protects against SARS-CoV-2 Beta variant challenge in hamsters, and viral passaging experiments reveal a high barrier for emergence of escape mutants, making it a good candidate for clinical development. The conserved ACE2-binding residues present a site of vulnerability that might be leveraged for developing vaccines eliciting broad sarbecovirus immunity.

## A site of vulnerability in SARS-COV-2

Neutralizing antibodies are a key defense against severe acute respiratory syndrome coronavirus 2 (SARS-CoV-2). Many neutralizing antibodies target the region of the viral spike protein that is involved in binding to the human ACE2 receptor, known as the receptor binding motif (RBM). This region of the protein is divergent between SARS-CoV-2 variants, leading to failure of existing monoclonal antibody treatments and evasion of antibodies elicited by previous infection or vaccination. Park *et al*. describe a monoclonal antibody that neutralizes a broad range of sarbecoviruses, including both the 2003 SARS-CoV and SARS-CoV-2. This antibody also binds in the RBM, but targets residues that are more conserved because they are involved in ACE2 binding. The antibody protects against the SARS-CoV-2 Beta variant, and none of the individual mutations in the Omicron variant affected antibody binding. —VV

## Teaser

An antibody neutralizes across sarbecoviruses that use ACE2 to enter cells by binding viral residues that interact with ACE2.

The zoonotic spillover of severe acute respiratory syndrome coronavirus 2 (SARS-CoV-2) has resulted in a global pandemic causing more than 266 million infections and more than 5.2 million fatalities as of December 2021. Continued SARS-CoV-2 evolution leads to the emergence of variants of concern (VOCs) that are characterized by higher transmissibility, immune evasion, and/or disease severity. For pandemic preparedness, we need pan-sarbecovirus countermeasures, such as vaccines and therapeutics that are effective against all SARS-CoV-2 variants and divergent zoonotic sarbecoviruses (*1*).

The coronavirus spike (S) glycoprotein promotes viral entry into host cells and is the main target of neutralizing antibodies elicited by infection or vaccination (*2*–*7*). The S protein comprises an S_1_ subunit, which recognizes host cell receptors, and an S_2_ subunit that drives viral cell membrane fusion. The S_1_ subunit includes the N-terminal domain and the receptor binding domain (RBD) and two additional domains designated C and D (*6*). For SARS-CoV and SARS-CoV-2, the RBD interacts with angiotensin-converting enzyme 2 (ACE2) to allow virus entry into host cells (*4*, *8*–*16*). The RBD is also the main target of serum neutralizing activity elicited by infection (*17*) and vaccination (*7*, *18*) and exposes multiple antigenic sites that are recognized by broadly neutralizing sarbecovirus antibodies (Abs) (*19*–*25*) (fig. S1). However, a large fraction of Abs in polyclonal sera (*17*) and most monoclonal Abs (mAbs) selected for therapeutic development (*26*) target a subset of epitopes that overlap the ACE2-contact surface [designated the receptor binding motif (RBM)]. The marked genetic divergence and plasticity of the RBM among SARS-CoV-2 variants and sarbecoviruses have thus far limited the breadth of Abs recognizing this region, and they are readily escaped by mutations (*20*, *27*–*32*).

**Fig. 1.**
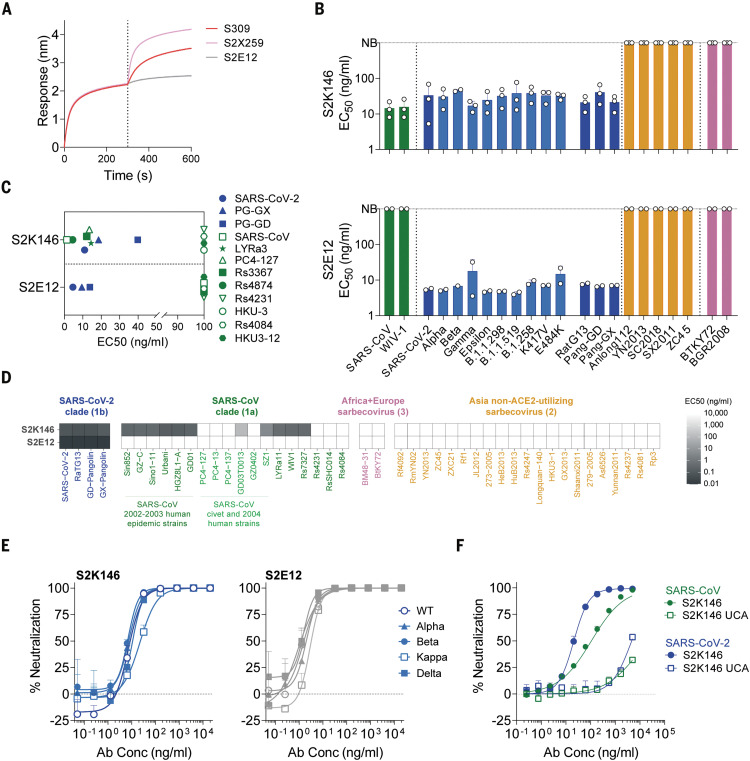
Identification of the S2K146 cross-reactive and broadly neutralizing sarbecovirus mAb. (**A**) Binding of site I–targeting S2E12 (*33*), site IV–targeting S309 (*19*), and site II–targeting S2X259 (*19*) recombinant IgG1 (second phase) after association of S2K146 mAb (first phase) to His-tagged SARS-CoV-2 RBD immobilized on anti-His sensors, as measured by biolayer interferometry. (**B**) Cross-reactivity of S2K146 (upper panel) and S2E12 (lower panel) with 22 sarbecovirus RBDs from four sarbecovirus clades and SARS-CoV-2 variants analyzed by ELISA. Median effective concentration (EC_50_) of at least two independent experiments is shown. Error bars indicate standard deviation between experimental repeats. (**C**) Flow cytometry analysis of S2K146 cross-reactivity with a panel of 12 S glycoproteins representative of sarbecovirus clades 1a and 1b transiently expressed on the surface of mammalian cells. (**D**) S2K146 cross-reactivity with sarbecovirus RBDs displayed at the surface of yeast. (**E**) S2K146- and S2E12-mediated neutralization of replication-competent SARS-CoV-2 (USA-WA1/2020) and SARS-CoV-2 VOC viruses. WT; wild type; Ab Conc, antibody concentration. (**F**) S2K146- and S2K146 UCA–mediated neutralization of VSV pseudotypes harboring SARS-CoV-2 S or SARS-CoV S. Error bars indicating standard deviation between replicates are represented only in one direction.

To identify broadly neutralizing sarbecovirus Abs, we isolated SARS-CoV-2 S-specific [immunoglobulin G (IgG)] memory B cells from one symptomatic COVID-19 convalescent individual (who was not hospitalized) 35 days after symptoms onset. We identified one mAb, designated S2K146 [immunoglobulin heavy and light variable genes 3-43 and 1-44, respectively (IGHV3-43; IGL1-44)], which did not compete with S309 (site IV) (*21*) or S2X259 (site II) (*19*) but competed with S2E12 (site I), a potent RBM mAb with neutralization breadth against SARS-CoV-2–related sarbecoviruses (*33*) (Fig. 1A and fig. S1). Like S2E12, S2K146 bound to all SARS-CoV-2 VOC RBDs as well as all clade 1b sarbecovirus RBDs tested by enzyme-linked immunosorbent assay (ELISA) (Fig. 1B and fig. S2A). In contrast to S2E12 and other site I–targeting Abs described so far, however, S2K146 also cross-reacted with the SARS-CoV and WIV-1 RBDs (clade 1a), which share 73 and 76% sequence identity with the SARS-CoV-2 RBD, respectively (Fig. 1B and fig. S2A). S2K146 did not bind to clades 2 and 3 sarbecovirus RBDs, similarly to the broadly neutralizing sarbecovirus S309 mAb but in contrast to the S2X259 and S2H97 (site V) mAbs (*19*, *20*). We observed S2K146 cross-reactivity with clades 1a and 1b sarbecoviruses using native S trimers transiently expressed on the surface of mammalian cells (Fig. 1C) and yeast surface–displayed RBDs (Fig. 1D), consistent with the ELISA results. On the basis of these results, we hypothesized that S2K146 recognizes a previously uncharacterized RBM epitope that is conserved among sarbecovirus clades 1a and 1b.

To evaluate the neutralization potency of the S2K146 mAb, we carried out dose-response inhibition assays using a vesicular stomatitis virus (VSV) pseudotyping platform. S2K146 efficiently blocked SARS-CoV and SARS-CoV-2 S-mediated entry into cells with median inhibitory concentration (IC_50_) of 108 and 16 ng/ml, respectively (fig. S2B). Moreover, S2K146 potently neutralized VSV pseudotypes harboring SARS-CoV-2 S glycoproteins from VOCs including Alpha, Beta, Gamma, Delta plus (AY.1/AY.2)_,_ Epsilon, and Lambda (fig. S2B). S2K146 also weakly neutralized VSV pseudotyped with BtKY72 S (clade 3) harboring the K493Y/T498W mutations (SARS-CoV-2 numbering) (fig. S2C), which enable human ACE2–mediated entry (*34*), whereas S2E12 did not recognize the wild-type or double-mutant BtKY72 RBD (fig. S3). Finally, S2K146 neutralized authentic SARS-CoV-2 (isolate USA-WA1/2020, lineage A, IC_50_ = 10 ng/ml) and SARS-CoV-2 VOCs (Alpha, IC_50_ = 9 ng/ml; Beta, IC_50_ = 9 ng/ml; Delta, IC_50_ = 8 ng/ml; Kappa, IC_50_ = 30 ng/ml) with a potency approaching that observed with the ultrapotent S2E12 mAb (*33*) (Wuhan-1, IC_50_ = 3.5 ng/ml; Alpha, IC_50_ = 2.5 ng/ml; Beta, IC_50_ = 2 ng/ml; Delta, IC_50_ = 1.5 ng/ml; Kappa, IC_50_ = 4.5 ng/ml) in a side-by-side comparison (Fig. 1E).

To assess the role of somatic mutations for S2K146 binding and neutralization, we generated its inferred unmutated common ancestor (S2K146 UCA). Alignment with the UCA amino acid sequence reveals that S2K146 harbors seven and two somatic hypermutations in the heavy- and light-chain complementarity determining regions (CDR), respectively [V_H_ (variable region of immunoglobulin heavy chain) identity: 94.4%; V_L_ (variable region of immunoglobulin light chain) identity: 98.9%] (fig. S2D). Except for WIV-1, S2K146 and S2K146 UCA showed no major cross-reactivity differences with a panel of RBDs representative of clade 1 sarbecoviruses, as determined by ELISA (fig. S2E). Nevertheless, biolayer interferometry revealed that S2K146 bound to prefusion-stabilized SARS-CoV and SARS-CoV-2 S ectodomain trimers with enhanced avidities compared with S2K146 UCA (fig. S2F). Accordingly, S2K146 UCA showed a marked loss in neutralizing activity against both SARS-CoV S and SARS-CoV-2 S VSV pseudotypes (Fig. 1F). Our results suggest that somatic hypermutations associated with S2K146 affinity maturation are especially important for enhancing mAb avidity and potency.

**Fig. 2.**
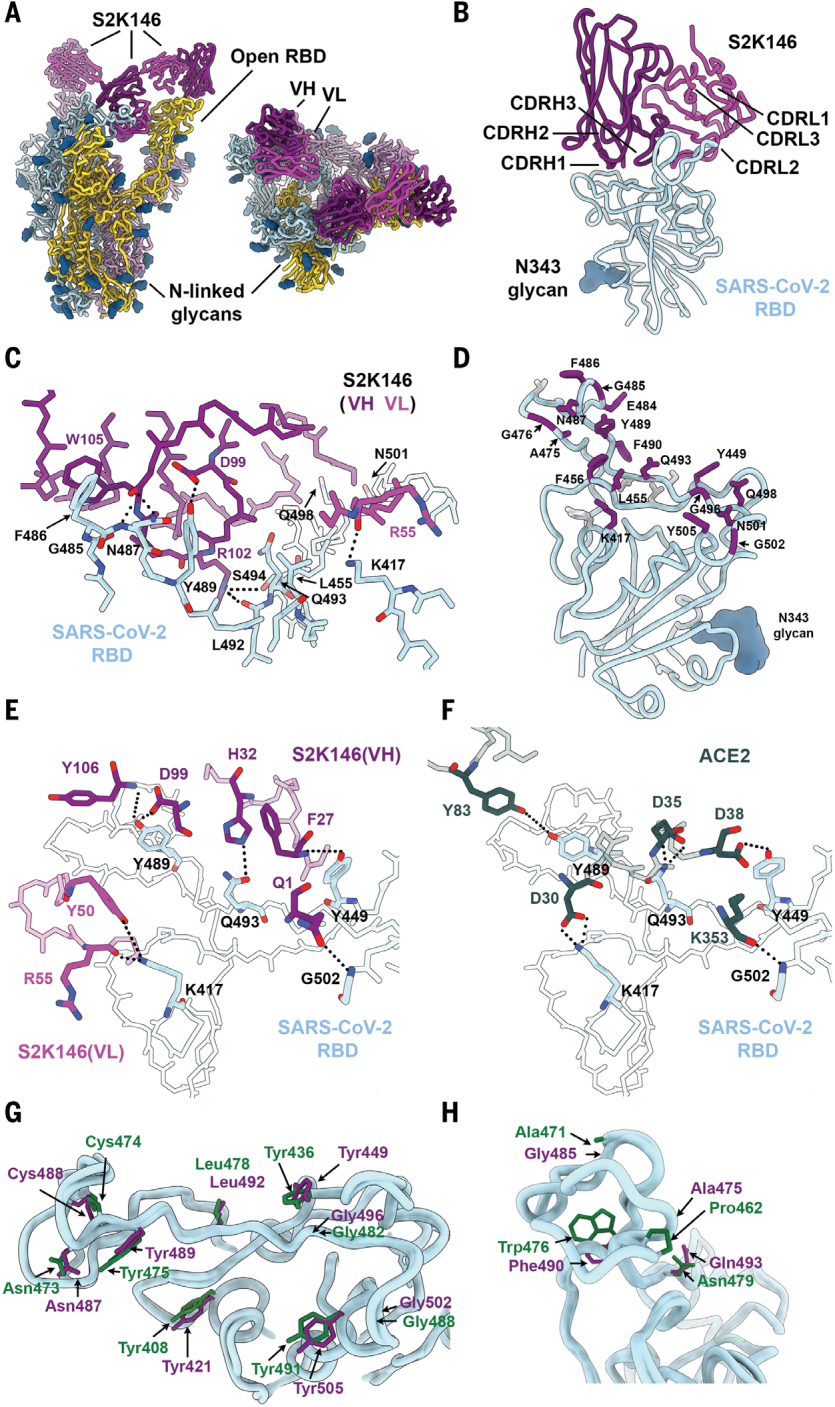
The S2K146 broadly neutralizing mAb recognizes RBD antigenic site I. (**A**) Cryo-EM structure viewed along two orthogonal orientations of the prefusion SARS-CoV-2 S ectodomain trimer with three S2K146 Fab fragments bound to two open RBDs and one partially closed RBD. SARS-CoV-2 S protomers are colored cyan, pink, and gold. S2K146 heavy chain and light chain variable domains are colored purple and magenta, respectively. Glycans are rendered as blue spheres. (**B**) Ribbon diagram of the S2K146-bound SARS-CoV-2 RBD. (**C**) Zoomed-in view of the contacts formed between S2K146 and the SARS-CoV-2 RBD. Selected epitope residues are shown as sticks, and electrostatic interactions are indicated with dotted lines. S2K146 heavy chain and light chain variable domains are colored as in (A). Single-letter abbreviations for the amino acid residues are as follows: A, Ala; C, Cys; D, Asp; E, Glu; F, Phe; G, Gly; H, His; I, Ile; K, Lys; L, Leu; M, Met; N, Asn; P, Pro; Q, Gln; R, Arg; S, Ser; T, Thr; V, Val; W, Trp; and Y, Tyr. (**D**) S2K146 epitope residues shown as sticks and colored purple (labeled) if they are involved in ACE2 binding or colored gray if not (unlabeled). (**E** and **F**) Similar electrostatic interactions (dotted lines) formed between S2K146 (E) or ACE2 (F) and the SARS-CoV-2 RBD. (**G**) The side chains of the nine S2K146 epitope residues conserved between the SARS-CoV-2 (purple) and SARS-CoV [green, Protein Data Bank (PDB) ID 2AJF (*12*)] RBDs are shown as sticks. (**H**) The side chains of the four S2K146 epitope residues conservatively substituted between the SARS-CoV-2 (purple) and SARS-CoV (green) RBDs are shown as sticks.

To understand the sarbecovirus cross-reactivity of the RBM-specific S2K146 mAb, we determined a cryo–electron microscopy (cryo-EM) structure of the S2K146 Fab fragment in complex with the SARS-CoV-2 S ectodomain trimer at 3.2-Å resolution (Fig. 2A, fig. S4, and table S1). Three-dimensional (3D) classification of the data led to the determination of a structure with three open RBDs, each bound to a S2K146 Fab, as well as a structure with two open RBDs and one closed RBD, with a Fab bound to each of them (fig. S4). Our cryo-EM data show that the opening of two RBDs is enough to allow three Fabs to bind to an S trimer, as the remaining closed RBD can engage an S2K146 Fab owing to its angle of approach.

To overcome the conformational heterogeneity of the S2K146-bound RBDs relative to the rest of the S trimer, we used focused 3D classification and local refinement of the S2K146 variable domains and RBD to obtain a reconstruction at 3.2-Å resolution enabling unambiguous model building and providing a detailed view of the binding interface (Fig. 2B, fig. S4, and table S1). S2K146 recognizes an epitope in antigenic site I (*17*), which overlaps with the RBM and is partially masked when the three RBDs adopt a closed state leading to clashes between the mAb and a neighboring RBD (Fig. 2, A and B, and fig. S1). The S2K146 paratope includes the heavy chain N terminus and CDRH1, CDRH2, and CDRH3, accounting for three-quarters of the surface buried upon binding, with light chain CDRL1, CDRL2, and CDRL3 making up the rest of the interface. A total of 1000 Å^2^ of the paratope surface is buried at the interface with the RBM through electrostatic interactions and shape complementarity.

The S2K146 footprint on the SARS-CoV-2 RBD highly resembles that of the ACE2 receptor, with 18 of 24 epitope residues shared with the ACE2 binding site, including key ACE2 contact positions L455, F486, Q493, Q498, and N501 (Fig. 2, C and D). Moreover, electrostatic interactions formed between S2K146 and the SARS-CoV-2 RBD recapitulate some of the contacts involved in ACE2 binding, such as with residues K417, Y449, Y489, Q493, and G502 (Fig. 2, E and F). Although some S2K146 contact residues are mutated in several variants, such as K417 (Beta and Gamma), L452 (Delta, Epsilon, and Kappa), E484 (Beta, Gamma, and Kappa), and N501 (Alpha, Beta, and Gamma), the retention of neutralization of these variants suggests that the binding interface is resilient to these residue substitutions (Fig. 1E and fig. S2B). The cross-reactivity with and broad neutralization of SARS-CoV by S2K146 may be partially explained by the strict conservation or conservative substitution of nine and four epitope residues relative to SARS-CoV-2, respectively (Fig. 2, G and H, and fig. S5A), consistent with the ability of both RBDs to bind human ACE2.

S2K146 therefore overcomes the mutational plasticity of the RBM, which is implicated in immune evasion, by targeting residues required for binding to the ACE2 receptor. This is supported by S2K146 recognition of the reconstructed RBD ancestor of SARS-CoV and SARS-CoV-2 (fig. S5, A and B), where human ACE2 binding first arose during sarbecovirus evolution (*34*), in line with the hypothesis that human ACE2 binding participates in conferring S2K146 susceptibility. As S2K146 does not compete with broadly neutralizing sarbecovirus mAbs targeting other antigenic sites, such as S309 (*21*) and S2X259 (*19*) (figs. S1 and S6), they could be combined in a cocktail to enhance breadth further and set an even higher barrier for emergence of escape mutants.

**Fig. 3.**
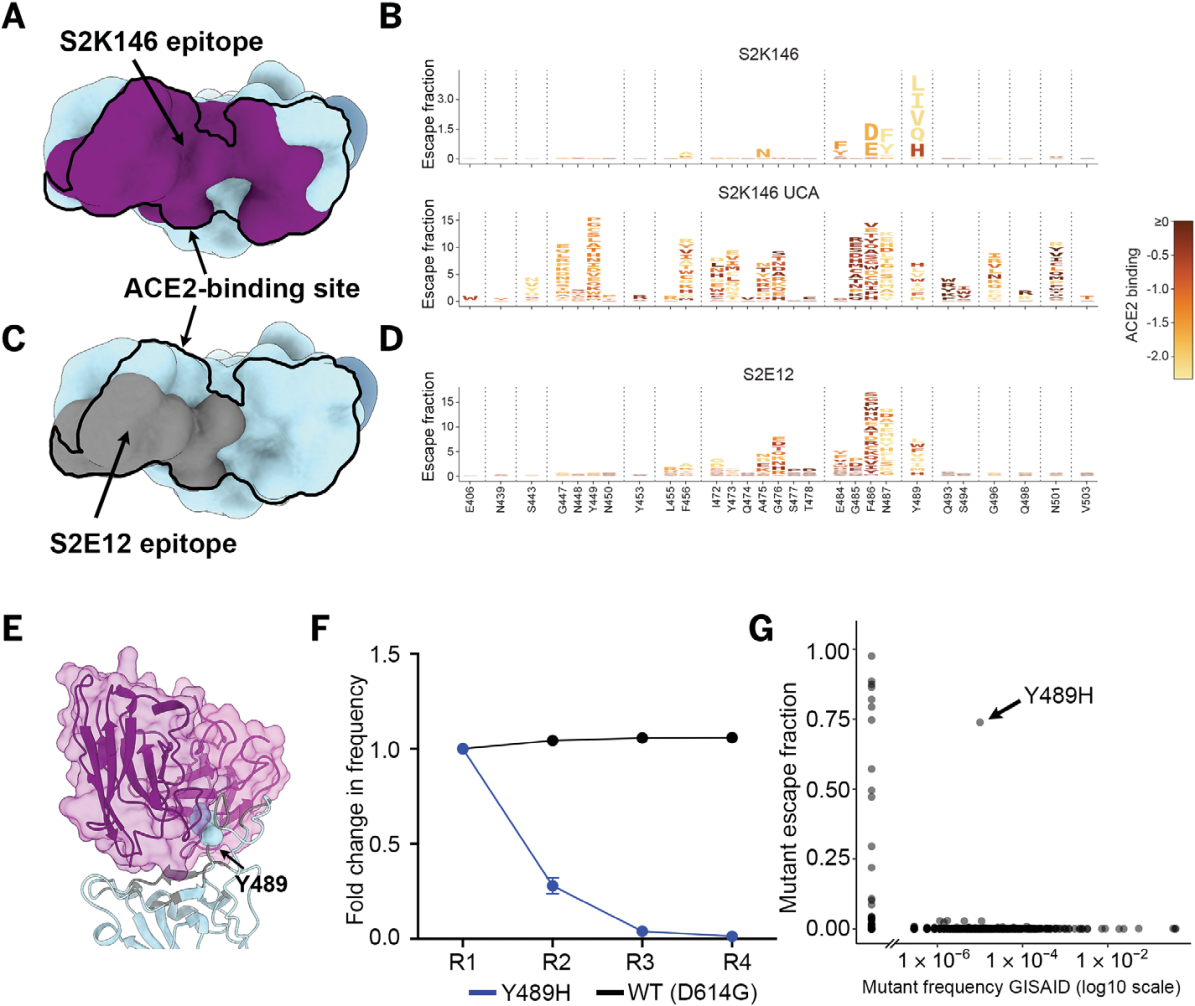
S2K146 is resilient to a broad spectrum of escape mutations. (**A**) Molecular surface representation of the SARS-CoV-2 RBD with the S2K146 epitope colored purple and the ACE2 footprint indicated as a black outline. (**B**) Mapping of RBD mutations reducing S2K146 (top) or S2K146 UCA (bottom) binding using DMS of the yeast-displayed SARS-CoV-2 RBD. Sites of strong escape (pink underlines in fig. S7A) are shown in logo plot. Letters are colored according to how mutations affect the ACE2 binding affinity of the SARS-CoV-2 RBD, as measured via yeast display by Starr *et al*. (*30*). (**C**) Molecular surface representation of the SARS-CoV-2 RBD with the S2E12 epitope colored gray and the ACE2 footprint indicated as a black outline. The N343 glycan is rendered as blue spheres in (A) and (C). (**D**) Mapping of RBD mutations reducing S2E12 binding using DMS of the yeast-displayed SARS-CoV-2 RBD. Sites of strong escape (purple underlines in fig. S7A) are shown in logo plot, as measured previously by Starr *et al.* (*20*). (**E**) Zoomed-in view of the S2K146-bound SARS-CoV-2 RBD (blue) highlighting the Y489H neutralization escape mutation. The S2K146 heavy and light chain variable domains are shown as ribbons within transparent purple and magenta surfaces, respectively. (**F**) Viral replication competition between VSV chimeras harboring the SARS-CoV-2 Wuhan-Hu-1/D614G S with or without the Y489H substitution using VeroE6 cells. (**G**) Mutations reducing binding of S2K146 to the RBD on the basis of DMS (escape score) are plotted versus their frequencies among the human-derived SARS-CoV-2 sequences on GISAID as of 27 September 2021. The large escape mutant (>5× global median escape fraction) with nonzero frequency is indicated.

To prospectively evaluate the impact of antigenic drift on S2K146 neutralization, we mapped RBD mutations that affect mAb binding using deep mutational scanning (DMS) of a yeast-displayed RBD mutant library covering all possible single residue substitutions in the Wuhan-Hu-1 RBD background (*30*). S2K146 binding was reduced by only a restricted number of amino acid substitutions compared to S2E12, which binds an overlapping but distinct epitope (Fig. 3, A to D, and fig. S7, A and B). All these mutations correspond to RBD residues buried upon ACE2 recognition (F456, A475, E484, F486, N487, and Y489) (Fig. 3B). Only one of these residue substitutions (Y489H) is accessible through a single-nucleotide change and could escape S2K146 recognition with a penalty on ACE2 binding affinity smaller than an order of magnitude, as determined by DMS data (*30*). None of the individual mutations present in the recently identified SARS-CoV-2 Omicron VOC affected S2K146 binding (Fig. 3B), although the effect of the full constellation of mutations remains to be evaluated. Conversely, DMS profiling of the S2K146 UCA revealed a greater number of binding-escape mutations, including some residue substitutions present in Omicron (e.g., Q493R or Q498R) (Fig. 3B). Therefore, the hotspot targeting of S2K146 on residues that are constrained in SARS-CoV-2 evolution appears to be a direct consequence of mAb affinity maturation.

To explore whether our escape map was consistent with in vitro viral evolution under mAb pressure, a replication competent VSV–SARS-CoV-2 S Wuhan-Hu-1/D614G chimera (*35*) was passaged in cell culture in the presence of the S2K146 mAb. Consistent with the DMS data, Y489H was the sole mutation resulting from a single nucleotide substitution that was detected in all the 36 neutralization-resistant plaques sampled (Fig. 3E, fig. S8A, and table S2). SARS-CoV-2 residue Y489 forms multiple interactions with S2K146 CDRH3 and accounts for ~10% of the total epitope buried surface area (Fig. 3E), in line with the major impact of the Y489H substitution on mAb neutralization. Of all the mutations at position 489 identified by DMS to reduce S2K146 binding (Fig. 3B), the Y-to-H substitution had the lowest impact on ACE2 binding, which might explain why it was the sole neutralization escape mutant selected upon passaging.

To evaluate the fitness of the Y489H mutant, we carried out a competition assay in which replicating VSV chimeras harboring the Wuhan-Hu-1/D614G S with or without the Y489H substitution were mixed at equal titers and passaged together without mAb. Because of the fitness cost associated with the mutation, which dampens ~4.5-fold the 1:1 ACE2-binding affinity to the SARS-CoV-2 RBD (fig. S8B), the Y489H S chimera was outcompeted by the Wuhan-Hu-1/D614G S chimera after only four rounds of passaging (Fig. 3F). Accordingly, only 29 out 2.9 million genomes were found to harbor the S Y489H mutation, underscoring the rarity of and the fitness cost imposed by this residue substitution (Fig. 3G). Collectively, these data illustrate the high barrier for emergence of escape mutants imposed by the S2K146 mAb, making it a good candidate for clinical development.

**Fig. 4.**
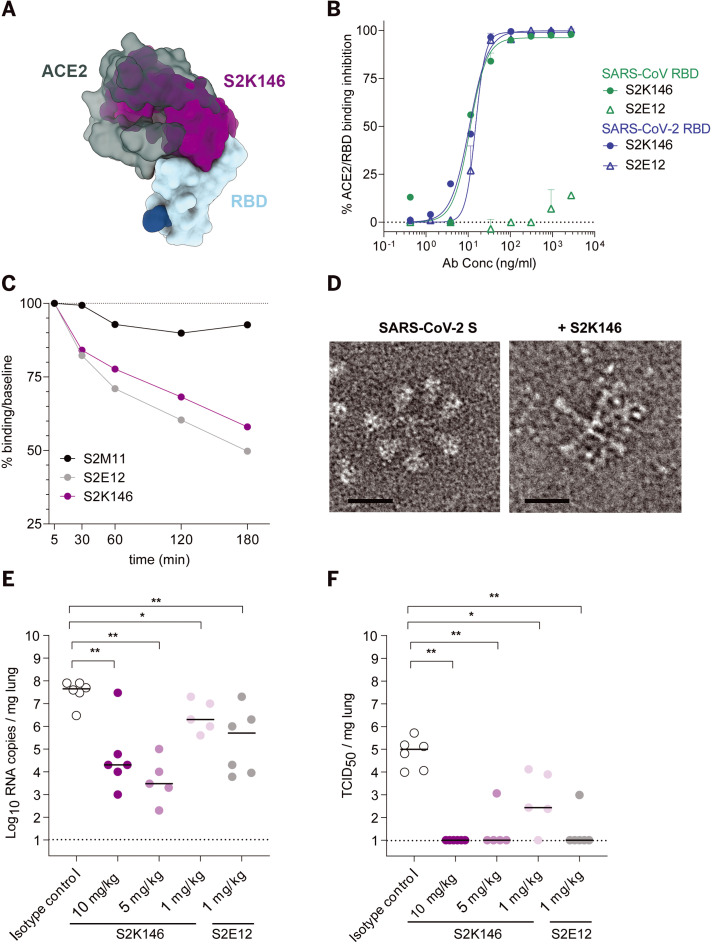
S2K146 blocks receptor attachment, triggers premature S refolding, and protects against SARS-CoV-2 challenge therapeutically. (**A**) Superimposition of the S2K146-bound (purple) and ACE2-bound [dark gray, PDB ID 6M0J (*16*)] SARS-CoV-2 RBD (light blue) structures showing steric overlap. The N343 glycan is rendered as blue spheres. (**B**) Preincubation of serial dilutions of S2K146 with the SARS-CoV-2 RBD prevents binding to immobilized human ACE2 (hACE2) ectodomain in ELISA. Error bars indicate standard deviation between replicates. (**C**) S2K146-mediated S_1_-shedding from cell surface–expressed SARS-CoV-2 S as determined by flow cytometry. S2E12 mAb was used as positive control, whereas S2M11 was used as a negative control. (**D**) Cropped electron micrographs of negatively stained SARS-CoV-2 S trimer before (left, prefusion state) or after (right, postfusion state) incubation with S2K146. One representative micrograph for each dataset is shown out of 93 (SARS-CoV-2 S alone) and 225 (SARS-CoV-2 S with S2K146) micrographs. Scale bars, 200 Å. (**E** and **F**) Quantification of viral RNA (E) and replicating virus titers [50% tissue culture infectious dose (TCID_50_)] (F) in the lung of Syrian hamster 4 days after intranasal infection with SARS-CoV-2 Beta VOC followed by therapeutic administration of S2K146 mAb at three different doses: 10, 5, or 1 mg/kg of body weight (*n* = 5 or 6 animals per group). S2E12 mAb was administered as control (*n* = 6 animals). Isotype control was administered at 10 mg/kg (*n* = 6 animals). *P < 0.5, **P < 0.01, as determined by Mann Whitney two-tailed test.

S2K146 targets antigenic site Ia, which overlaps with the RBM, indicating that mAb binding would compete with ACE2 attachment to the RBD via steric hindrance (Fig. 4A). Indeed, S2K146 inhibited binding of the SARS-CoV-2 and SARS-CoV RBDs to human ACE2 in a concentration-dependent manner, as measured by competition ELISA (Fig. 4B). As S2K146 conformationally selects for open RBDs, we assessed whether the mAb could promote shedding of the S_1_ subunit from cell surface–expressed full-length SARS-CoV-2 S, similar to some other RBD-specific mAbs (*17*, *19*, *24*, *33*). S2K146 induced shedding of the S_1_ subunit as efficiently as the RBM-targeting S2E12 mAb, whereas the control mAb S2M11, which locks S in the prefusion closed state, did not (*19*) (Fig. 4C). Furthermore, S2K146 Fab triggered fusogenic rearrangement of a wild-type–like S ectodomain trimer, as previously described for several SARS-CoV and SARS-CoV-2 neutralizing mAbs (*20*, *36*–*38*) (Fig. 4D). Thus S2K146-mediated sarbecovirus neutralization relies on competitively blocking viral attachment to the ACE2 receptor and putative inactivation of S trimers at the surface of virions before encountering host cells.

The efficient S2K146-induced S_1_ shedding could explain the lack of FcγRIIa and FcγRIIIa activation, which we used as a proxy for Ab-dependent cellular phagocytosis and Ab-dependent cellular cytotoxicity, respectively (fig. S9, A and B). However, S2K146 also did not activate FcγRIIa and triggered FcγRIIIa only weakly when target cells expressed an uncleavable prefusion-stabilized SARS-CoV-2 S protein (fig. S9, C and D). The greater efficiency of S2E12 for activating FcγRIIIa, relative to S2K146, might be explained by the different angles of approach at which these two mAbs bind to the RBD (fig. S9, E and F).

Next, we evaluated the therapeutic activity of S2K146 against challenge with the SARS-CoV-2 Beta VOC in a Syrian hamster model of infection (*39*, *40*). S2K146 was administered in doses of 1, 5, and 10 mg/kg of body weight via intraperitoneal injection 24 hours after intranasal challenge with SARS-CoV-2, and the lungs of the animals were collected 3 days later for the quantification of viral RNA and replicating virus. In parallel, six animals were administered 1 mg/kg of the ultrapotent S2E12 mAb for benchmarking (*33*). Viral RNA loads in the lungs were reduced by ~1, 4, and 3 orders of magnitude after receiving 1, 5, and 10 mg/kg of S2K146, respectively (Fig. 4E). Viral replication in the lungs was completely abrogated for the 5 and 10 mg/kg groups and reduced by greater than 2.5 orders of magnitude for the 1 mg/kg group (Fig. 4F). Overall serum mAb concentrations measured at day 4 after infection inversely correlated with viral RNA loads and infectious virus in the lungs (fig. S10, A and B). S2K146 therefore effectively protects against SARS-CoV-2 challenge in vivo in a stringent therapeutic setting.

The SARS-CoV-2 RBD accounts for most serum neutralizing activity in both COVID-19 convalescent (*17*, *41*) and vaccinated individuals (*7*, *18*), and a subset of antigenic sites are targeted by broadly neutralizing sarbecovirus Abs (*19*–*25*). RBD-based subunit vaccines and mRNA vaccines based on chimeric S glycoproteins elicit broadly neutralizing sarbecovirus Abs and heterotypic protection in vivo (*42*–*46*). Most of the Abs with broad neutralizing activity are expected to target conserved RBD epitopes, owing to their much greater potency and protection efficacy compared to Abs that target the conserved fusion machinery (*47*–*52*). The discovery of a functionally constrained and conserved RBM epitope associated with broad sarbecovirus neutralization is consistent with the strong cross-reactivity with the SARS-CoV RBM observed with polyclonal Abs elicited by a clinical stage SARS-CoV-2 vaccine in nonhuman primates (*44*) and will guide the development of next-generation pan-sarbecovirus vaccines to protect from future zoonotic transmission events.

The broadly neutralizing sarbecovirus mAb S309 was isolated from a survivor of a 2003 SARS-CoV infection, and its derivative (sotrovimab) has received emergency use authorizations in several countries around the world for the early treatment of mild-to-moderate COVID-19 in adults and some pediatric patients who test positive for SARS-CoV-2 by direct viral testing and who are at high risk for progression to severe COVID-19, including hospitalization or death (*19*, *27*, *29*, *53*). S309 has proven resilient to the emergence of SARS-CoV-2 variants in preclinical studies, possibly owing to targeting of a conserved RBD epitope with very limited mutational tolerance (*20*, *53*). The mechanism of S2K146-mediated ACE2 molecular mimicry also provides a high barrier for emergence of escape mutants in spite of the known mutational plasticity of the SARS-CoV-2 RBM (*30*). Therefore, the discovery of the S2K146 mAb might be a milestone for future treatment of COVID-19 patients and for pandemic preparedness against divergent sarbecoviruses.

## Supplementary Material

20220107-1

## Supplementary Materials

### This PDF file includes:

Materials and Methods Figs. S1 to S10 Tables S1 and S2 References (*54*–*76*)

### Other Supplementary Material for this manuscript includes the following:

MDAR Reproducibility Checklist

## Notes

View/request a protocol for this paper from *Bio-protocol*.
