# Supplementary material for: Antibody-mediated broad sarbecovirus neutralization through ACE2 molecular mimicry
Source: Science. 2022 Jan 6;375(6579):449–54. doi: 10.1126/science.abm8143 (PMC9400459; doi:10.1126/science.abm8143)
Supplement: 20220107-1 [file science.abm8143.v1.pdf]

Cite as: Y.-J. Park *et al.*, *Science* 10.1126/science.abm8143 (2022).

Antibody-mediated broad sarbecovirus neutralization through ACE2 molecular mimicry

Young-Jun Park[1,2]†, Anna De Marco[3]†, Tyler N. Starr[4]†, Zhuoming Liu[5]†, Dora Pinto[3], Alexandra C. Walls[1,2], Fabrizia Zatta[3], Samantha K. Zepeda[1], John E. Bowen[1], Kaitlin R. Sprouse[1], Anshu Joshi[1], Martina Giurdanella[3], Barbara Guarino[3], Julia Noack[6], Rana Abdelnabi[7], Shi-Yan Caroline Foo[7], Laura E. Rosen[6], Florian A. Lempp[6], Fabio Benigni[3], Gyorgy Snell[6], Johan Neyts[7], Sean P. J. Whelan[5], Herbert W. Virgin[6,8,9], Jesse D. Bloom[2,4], Davide Corti[3]*, Matteo Samuele Pizzuto[3]*, David Veesler[1,2]*

[1]Department of Biochemistry, University of Washington, Seattle, WA 98195, USA. [2]Howard Hughes Medical Institute, University of Washington, Seattle, WA 98195, USA. [3]Humabs Biomed SA, a subsidiary of Vir Biotechnology, 6500 Bellinzona, Switzerland. [4]Basic Sciences Division, Fred Hutchinson Cancer Research Center, Seattle, WA 98109, USA. [5]Department of Molecular Microbiology, Washington University School of Medicine, St. Louis, MO 63110, USA. [6]Vir Biotechnology, San Francisco, CA 94158, USA. [7]Laboratory of Virology and Chemotherapy, Rega Institute for Medical Research, KU Leuven, 3000 Leuven, Belgium. [8]Department of Pathology and Immunology, Washington University School of Medicine, St. Louis, MO 63110, USA. [9]Department of Internal Medicine, University of Texas Southwestern Medical Center, Dallas, TX 75390, USA.

†These authors contributed equally to this work.

***Corresponding author. Email: dveesler@uw.edu (D.V.); mpizzuto@vir.bio (M.S.P.); dcorti@vir.bio (D.C.)**

Understanding broadly neutralizing sarbecovirus antibody responses is key to developing countermeasures against SARS-CoV-2 variants and future zoonotic sarbecoviruses. We describe the isolation and characterization of a human monoclonal antibody, designated S2K146, that broadly neutralizes viruses belonging to SARS-CoV- and SARS-CoV-2-related sarbecovirus clades which use ACE2 as an entry receptor. Structural and functional studies show that most of the virus residues that directly bind S2K146 are also involved in binding to ACE2. This allows the antibody to potently inhibit receptor attachment. S2K146 protects against SARS-CoV-2 Beta challenge in hamsters and viral passaging experiments reveal a high barrier for emergence of escape mutants, making it a good candidate for clinical development. The conserved ACE2-binding residues present a site of vulnerability that might be leveraged for developing vaccines eliciting broad sarbecovirus immunity.

The zoonotic spillover of SARS-CoV-2 has resulted in a global pandemic causing over 266 million infections and more than 5.2 million fatalities as of December 2021. Continued SARS-CoV-2 evolution leads to the emergence of variants of concern (VOC) that are characterized by higher transmissibility, immune evasion or disease severity. For pandemic preparedness, we need pan-sarbecovirus countermeasures, such as vaccines and therapeutics that are effective against all SARS-CoV-2 variants and divergent zoonotic sarbecoviruses (*1*).

The coronavirus spike glycoprotein (S) promotes viral entry into host cells and is the main target of neutralizing antibodies elicited by infection or vaccination (*2*–*7*). S comprises an S_1 subunit, which recognizes host cell receptors, and an S_2 subunit that drives viral-cell membrane fusion. The S_1 subunit includes the N-terminal domain and the receptor-binding domain (RBD) and two additional domains designated C and D (*6*). For SARS-CoV and SARS-CoV-2, the RBD interacts with angiotensin-converting enzyme 2 (ACE2) to allow virus entry into host cells (*4*, *8*–*16*). The RBD is also the main target of serum neutralizing activity elicited by infection (*17*) and vaccination (*7*, *18*) and exposes multiple antigenic sites that are recognized by broadly neutralizing sarbecovirus antibodies (Abs) (*19*–*25*) (fig. S1). However, a large fraction of Abs in polyclonal sera (*17*) and most monoclonal Abs (mAbs) selected for therapeutic development (*26*) target a subset of epitopes that overlap the ACE2-contact surface [designated the receptor-binding motif (RBM)]. The marked genetic divergence and plasticity of the RBM among SARS-CoV-2 variants and sarbecoviruses has thus far limited the breadth of Abs recognizing this region and they are readily escaped by mutations (*20*, *27*–*32*).

To identify broadly neutralizing sarbecovirus Abs, we isolated SARS-CoV-2 S-specific (IgG) memory B cells from one symptomatic COVID-19 convalescent individual (who was not hospitalized) 35 days after symptoms onset. We identified one mAb, designated S2K146 (IGHV3-43; IGL1-44), which did not compete with S309 (site IV) (*21*) or S2X259 (site II) (*19*) but competed with S2E12 (site I), a potent RBM mAb with neutralization breadth against SARS-CoV-2-related

sarbecoviruses (*33*) (Fig. 1A and fig. S1). Similar to S2E12, S2K146 bound to all SARS-CoV-2 VOC RBDs as well as all clade 1b sarbecovirus RBDs tested by ELISA (Fig. 1B and fig. S2A). In contrast to S2E12 and other site I-targeting Abs described so far, however, S2K146 also cross-reacted with the SARS-CoV and WIV-1 RBDs (clade 1a), which share 73% and 76% sequence identity with the SARS-CoV-2 RBD, respectively (Fig. 1B and fig. S2A). S2K146 did not bind to clades 2 and 3 sarbecovirus RBDs, similarly to the broadly neutralizing sarbecovirus S309 mAb but in contrast to the S2X259 and S2H97 (site V) mAbs (*19*, *20*). Consistent with the ELISA results, we observed S2K146 cross-reactivity with clades 1a and 1b sarbecoviruses using native S trimers transiently expressed on the surface of mammalian cells (Fig. 1C) and yeast-surface displayed RBDs (Fig. 1D). Based on these results, we hypothesized that S2K146 recognizes a previously uncharacterized RBM epitope which is conserved among sarbecovirus clades 1a and 1b.

To evaluate the neutralization potency of the S2K146 mAb, we carried out dose-response inhibition assays using a vesicular stomatitis virus (VSV) pseudotyping platform. S2K146 efficiently blocked SARS-CoV S- and SARS-CoV-2 S-mediated entry into cells with half maximum inhibitory concentration (IC_{50}) of 108 and 16 ng/ml, respectively (fig. S2B). Moreover, S2K146 potently neutralized VSV pseudotypes harboring SARS-CoV-2 S glycoproteins from VOCs including Alpha, Beta, Gamma, Delta plus (AY.1/AY.2), Epsilon and Lambda (fig. S2B). S2K146 also weakly neutralized VSV pseudotyped with BtKY72 S (clade 3) harboring the K493Y/T498W mutations (SARS-CoV-2 numbering) (fig. S2C), which enable human ACE2-mediated entry (*34*), whereas S2E12 did not recognize the wildtype or double mutant BtKY72 RBD (fig. S3). Finally, S2K146 neutralized authentic SARS-CoV-2 (isolate USA-WA1/2020, lineage A, IC_{50} = 10 ng/ml) and SARS-CoV-2 VOC (Alpha, IC_{50} = 9 ng/ml; Beta, IC_{50} = 9 ng/ml; Delta, IC_{50} = 8 ng/ml; Kappa, IC_{50} = 30 ng/ml) with a potency approaching that observed with the ultrapotent S2E12 mAb (*33*) (Wuhan-1, IC_{50} = 3.5 ng/ml; Alpha, IC_{50} = 2.5 ng/ml; Beta, IC_{50} = 2 ng/ml; Delta, IC_{50} = 1.5 ng/ml; Kappa, IC_{50} = 4.5 ng/ml) in a side-by-side comparison (Fig. 1E).

To assess the role of somatic mutations for S2K146 binding and neutralization, we generated its inferred unmutated common ancestor (S2K146 UCA). Alignment with the UCA amino acid sequence reveals that S2K146 harbors seven and two somatic hypermutations in the heavy- and light-chain complementarity determining regions (CDR), respectively (VH identity: 94.4% and VL identity: 98.9%, fig. S2D). Except for WIV1, S2K146 and S2K146 UCA showed no major cross-reactivity differences with a panel of RBDs representative of clade 1 sarbecoviruses, as determined by ELISA (fig. S2E). Nevertheless, biolayer interferometry revealed that S2K146 bound to prefusion-stabilized SARS-CoV and SARS-CoV-2 S ectodomain trimers with enhanced avidities compared to S2K146 UCA (fig. S2F). Accordingly, S2K146 UCA showed a marked loss in neutralizing activity against both SARS-CoV S and SARS-CoV-2 S VSV pseudotypes (Fig. 1F). Our results suggest that somatic hypermutations associated with S2K146 affinity maturation are especially important to enhance mAb avidity and potency.

To understand the sarbecovirus cross-reactivity of the RBM-specific S2K146 mAb, we determined a cryo-electron microscopy structure of the S2K146 Fab fragment in complex with the SARS-CoV-2 S ectodomain trimer at 3.2 Å resolution (Fig. 2A, fig. S4, and table S1). 3D classification of the data led to the determination of a structure with three open RBDs, each bound to a S2K146 Fab, as well as a structure with two open RBDs and one closed RBD, with a Fab bound to each of them (fig. S4). Our cryoEM data show that opening of two RBDs is enough to allow three Fabs to bind to an S trimer, as the remaining closed RBD can engage an S2K146 Fab due to its angle of approach.

To overcome the conformational heterogeneity of the S2K146-bound RBDs relative to the rest of the S trimer, we used focused 3D classification and local refinement of the S2K146 variable domains and RBD to obtain a reconstruction at 3.2 Å resolution enabling unambiguous model building and providing a detailed view of the binding interface (Fig. 2B, fig. S4, and table S1). S2K146 recognizes an epitope in antigenic site I (*17*), which overlaps with the RBM and is partially masked when the three RBDs adopt a closed state leading to clashes between the mAb and a neighboring RBD (Fig. 2, A and B, and fig. S1). The S2K146 paratope includes the heavy chain N terminus and CDRH1, H2 and H3, accounting for 3/4 of the surface buried upon binding, with light chain CDRL1, L2 and L3, making up the rest of the interface. A total of 1,000 Å^2 of the paratope surface is buried at the interface with the RBM through electrostatic interactions and shape complementarity.

The S2K146 footprint on the SARS-CoV-2 RBD strikingly resembles that of the ACE2 receptor with 18 out of 24 epitope residues shared with the ACE2-binding site, including key ACE2-contact positions L455, F486, Q493, Q498 and N501 (Fig. 2, C and D). Moreover, electrostatic interactions formed between S2K146 and the SARS-CoV-2 RBD recapitulate some of the contacts involved in ACE2 binding, such as with residues K417, Y449, Y489, Q493 and G502 (Fig. 2, E and F). Although some S2K146 contact residues are mutated in several variants, such as K417 (Beta and Gamma), L452 (Delta, Epsilon and Kappa), E484 (Beta, Gamma and Kappa) and N501 (Alpha, Beta and Gamma), the retention of neutralization of these variants suggests that the binding interface is resilient to these residue substitutions (Fig. 1E and fig. S2B). The cross-reactivity with and broad neutralization of SARS-CoV by

S2K146 may be partially explained by the strict conservation or conservative substitution of nine and four epitope residues relative to SARS-CoV-2, respectively (Fig. 2, G and H, and fig. S5A), consistent with the ability of both RBDs to bind human ACE2.

S2K146 therefore overcomes the mutational plasticity of the RBM, that is implicated in immune evasion, by targeting residues required for binding to the ACE2 receptor. This is supported by S2K146 recognition of the reconstructed RBD ancestor of SARS-CoV and SARS-CoV-2 (fig. S5, A and B), where human ACE2 binding first arose during sarbecovirus evolution (*34*), in line with the hypothesis that human ACE2 binding participates in conferring S2K146 susceptibility. As S2K146 does not compete with broadly neutralizing sarbecovirus mAbs targeting other antigenic sites, such as S309 (*21*) and S2X259 (*19*) (figs. S1 and S6), they could be combined in a cocktail to enhance breadth further and set an even higher barrier for emergence of escape mutants.

To prospectively evaluate the impact of antigenic drift on S2K146 neutralization, we mapped RBD mutations that affect mAb binding using deep-mutational scanning (DMS) of a yeast-displayed RBD mutant library covering all possible single residue substitutions in the Wuhan-Hu-1 RBD background (*30*). S2K146 binding was reduced by only a restricted number of amino acid substitutions, compared to S2E12 which binds an overlapping but distinct epitope (Fig. 3, A to D, and fig. S7, A and B). All these mutations correspond to RBD residues buried upon ACE2 recognition (F456, A475, E484, F486, N487 and Y489) (Fig. 3B). Only one of these residue substitutions (Y489H) is accessible through a single-nucleotide change and could escape S2K146 recognition with a penalty on ACE2 binding affinity smaller than an order of magnitude, as determined by DMS data (*30*). None of the individual mutations present in the recently identified SARS-CoV-2 Omicron VOC impacted S2K146 binding (Fig. 3B), although the effect of the full constellation of mutations remains to be evaluated. Conversely, DMS profiling of the S2K146 UCA revealed a greater number of binding-escape mutations, including some residue substitutions present in Omicron (e.g., Q493K or Q498R) (Fig. 3B). Therefore, the hotspot targeting of S2K146 on residues which are constrained in SARS-CoV-2 evolution appears to be a direct consequence of mAb affinity maturation.

To explore whether our escape map was consistent with in vitro viral evolution under mAb pressure, a replication competent VSV-SARS-CoV-2 S Wuhan-Hu-1/D614G chimera (*35*) was passaged in cell culture in the presence of the S2K146 mAb. Consistent with the DMS data, Y489H was the sole mutation resulting from a single nucleotide substitution that was detected in all the 36 neutralization-resistant plaques sampled (Fig. 3E, fig. S8A, and table S2). SARS-CoV-2 residue Y489 forms multiple interactions with S2K146 CDRH3 and accounts for ~10% of the total epitope buried surface area (Fig. 3E), in line with the major impact of the Y489H substitution on mAb neutralization. Out of all the mutations at position 489 identified by DMS to reduce S2K146 binding (Fig. 3B), the Y to H substitution had the lowest impact on ACE2 binding, which might explain why it was the sole neutralization escape mutant selected upon passaging.

To evaluate the fitness of the Y489H mutant, we carried out a competition assay in which replicating VSV chimeras harboring the Wuhan-Hu-1/D614G S with or without the Y489H substitution were mixed at equal titers and passaged together without mAb. Due to the fitness cost associated with the mutation, which dampens ~4.5 fold the 1:1 ACE2-binding affinity to the SARS-CoV-2 RBD (fig. S8B), the Y489H S chimera was outcompeted by the Wuhan-Hu-1/D614G S chimera after only four rounds of passaging (Fig. 3F). Accordingly, only 29 out 2.9 million genomes were found to harbor the S Y489H mutation, underscoring the rarity of and the fitness cost imposed by this residue substitution (Fig. 3G). Collectively, these data illustrate the high barrier for emergence of escape mutants imposed by the S2K146 mAb, making it a good candidate for clinical development.

S2K146 targets antigenic site Ia which overlaps with the RBM, indicating that mAb binding would compete with ACE2 attachment to the RBD via steric hindrance (Fig. 4A). Indeed, S2K146 inhibited binding of the SARS-CoV-2 and SARS-CoV RBDs to human ACE2 in a concentration-dependent manner, as measured by competition ELISA (Fig. 4B). As S2K146 conformationally selects for open RBDs, we assessed if the mAb could promote shedding of the S_1 subunit from cell-surface-expressed full-length SARS-CoV-2 S, similar to some other RBD-specific mAbs (*17*, *19*, *24*, *33*). S2K146 induced shedding of the S_1 subunit as efficiently as the RBM-targeting S2E12 mAb, whereas the control mAb S2M11, which locks S in the prefusion closed state, did not (*19*) (Fig. 4C). Furthermore, S2K146 Fab triggered fusogenic rearrangement of a wildtype-like S ectodomain trimer, as previously described for several SARS-CoV and SARS-CoV-2 neutralizing mAbs (*20*, *36*–*38*) (Fig. 4D). Thus S2K146-mediated sarbecovirus neutralization relies on competitively blocking viral attachment to the ACE2 receptor and putative inactivation of S trimers at the surface of virions before encountering host cells.

The efficient S2K146-induced S_1 shedding could explain the lack of FcγRIIa and FcγRIIIa activation, which we used as a proxy for Ab-dependent cellular phagocytosis and Ab-dependent cellular cytotoxicity, respectively (fig. S9, A and B). However, S2K146 also did not activate FcγRIIa and triggered FcγRIIIa only weakly when target cells expressed an uncleavable prefusion-stabilized SARS-CoV-2 S protein (fig. S9, C and D). The greater efficiency of S2E12 for activating FcγRIIIa, relative to S2K146, might be explained by the different angles of approach at which these two mAbs bind to the RBD (fig.

S9, E and F).

Next, we evaluated the therapeutic activity of S2K146 against challenge with the SARS-CoV-2 Beta VOC in a Syrian hamster model of infection (*39*, *40*). S2K146 was administered at 1, 5 and 10 mg/kg via intraperitoneal injection 24 hours after intranasal challenge with SARS-CoV-2 and the lungs of the animals were collected 3 days later for the quantification of viral RNA and replicating virus. In parallel, 6 animals were administered 1 mg/kg of the ultrapotent S2E12 mAb for benchmarking (*33*). Viral RNA loads in the lungs were reduced approximately by 1, 4 and 3 orders of magnitude after receiving 1, 5 and 10 mg/kg of S2K146, respectively (Fig. 4E). Viral replication in the lungs was completely abrogated for the 5 and 10 mg/kg groups and reduced by greater than 2.5 orders of magnitude for the 1 mg/kg group (Fig. 4F). Overall serum mAb concentrations measured at day 4 post-infection inversely correlated with viral RNA loads and infectious virus in the lungs (fig. S10, A and B). S2K146 therefore effectively protects against SARS-CoV-2 challenge in vivo in a stringent therapeutic setting.

The SARS-CoV-2 RBD accounts for most serum neutralizing activity in both COVID-19 convalescent (*17*, *41*) and vaccinated individuals (*7*, *18*) and a subset of antigenic sites are targeted by broadly neutralizing sarbecovirus Abs (*19*–*25*). RBD-based subunit vaccines and mRNA vaccines based on chimeric S glycoproteins elicit broadly neutralizing sarbecovirus Abs and heterotypic protection in vivo (*42*–*46*). Most of the Abs with broad neutralizing activity are expected to target conserved RBD epitopes, due to their much greater potency and protection efficacy compared to Abs that target the conserved fusion machinery (*47*–*52*). The discovery of a functionally constrained and conserved RBM epitope associated with broad sarbecovirus neutralization is consistent with the strong cross-reactivity with the SARS-CoV RBM observed with polyclonal Abs elicited by a clinical stage SARS-CoV-2 vaccine in non-human primates (*44*) and will guide the development of next-generation pan-sarbecovirus vaccines to protect from future zoonotic transmission events.

The broadly neutralizing sarbecovirus mAb S309 was isolated from a survivor of a 2003 SARS-CoV infection and its derivative (sotrovimab) has received emergency use authorizations in several countries around the world for the early treatment of mild-to-moderate COVID-19 in adults and some pediatric patients who test positive for SARS-CoV-2 by direct viral testing, and who are at high risk for progression to severe COVID-19, including hospitalization or death (*19*, *27*, *29*, *53*). S309 has proven resilient to the emergence of SARS-CoV-2 variants in pre-clinical studies, possibly due to targeting of a conserved RBD epitope with very limited mutational tolerance (*20*, *53*). The mechanism of S2K146-mediated ACE2 molecular mimicry also provides a high barrier for emergence of escape mutants in spite of the known mutational plasticity of the SARS-CoV-2 RBM (*30*). Therefore, the discovery of the S2K146 mAb might be a milestone for future treatment of COVID-19 patients and for pandemic preparedness against divergent sarbecoviruses.

REFERENCES AND NOTES

1. C. A. Sánchez, H. Li, K. L. Phelps, C. Zambrana-Torrelio, L.-F. Wang, K. J. Olival, P. Daszak, A strategy to assess spillover risk of bat SARS-related coronaviruses in Southeast Asia. medRxiv 2021.09.09.21263359 [Preprint] (2021). https://doi.org/10.1101/2021.09.09.21263359.
2. A. C. Walls, M. A. Tortorici, B. J. Bosch, B. Frenz, P. J. M. Rottier, F. DiMaio, F. A. Rey, D. Veesler, Cryo-electron microscopy structure of a coronavirus spike glycoprotein trimer. *Nature* **531**, 114–117 (2016). doi:10.1038/nature16988 Medline
3. A. C. Walls, M. A. Tortorici, J. Snijder, X. Xiong, B. J. Bosch, F. A. Rey, D. Veesler, Tectonic conformational changes of a coronavirus spike glycoprotein promote membrane fusion. *Proc. Natl. Acad. Sci. U.S.A.* **114**, 11157–11162 (2017). doi:10.1073/pnas.1708727114 Medline
4. A. C. Walls, Y. J. Park, M. A. Tortorici, A. Wall, A. T. McGuire, D. Veesler, Structure, function, and antigenicity of the SARS-CoV-2 spike glycoprotein. *Cell* **181**, 281–292.e6 (2020). doi:10.1016/j.cell.2020.02.058 Medline
5. D. Wrapp, N. Wang, K. S. Corbett, J. A. Goldsmith, C. L. Hsieh, O. Abiona, B. S. Graham, J. S. McLellan, Cryo-EM structure of the 2019-nCoV spike in the prefusion conformation. *Science* **367**, 1260–1263 (2020). doi:10.1126/science.abb2507 Medline
6. M. A. Tortorici, D. Veesler, Structural insights into coronavirus entry. *Adv. Virus Res.* **105**, 93–116 (2019). doi:10.1016/bs.aivir.2019.08.002 Medline
7. L. Stamatatos, J. Czartoski, Y.-H. Wan, L. J. Homad, V. Rubin, H. Glantz, M. Neradilek, E. Seydoux, M. F. Jennewein, A. J. MacCamy, J. Feng, G. Mize, S. C. De Rosa, A. Finzi, M. P. Lemos, K. W. Cohen, Z. Moodie, M. J. McElrath, A. T. McGuire, mRNA vaccination boosts cross-variant neutralizing antibodies elicited by SARS-CoV-2 infection. *Science* **372**, 1413–1418 (2021). doi:10.1126/science.abg9175 Medline
8. P. Zhou, X. L. Yang, X. G. Wang, B. Hu, L. Zhang, W. Zhang, H. R. Si, Y. Zhu, B. Li, C. L. Huang, H. D. Chen, J. Chen, Y. Luo, H. Guo, R. D. Jiang, M. Q. Liu, Y. Chen, X. R. Shen, X. Wang, X. S. Zheng, K. Zhao, Q. J. Chen, F. Deng, L. L. Liu, B. Yan, F. X. Zhan, Y. Y. Wang, G. F. Xiao, Z. L. Shi, A pneumonia outbreak associated with a new coronavirus of probable bat origin. *Nature* **579**, 270–273 (2020). doi:10.1038/s41586-020-2012-7 Medline
9. M. Letko, A. Marzi, V. Munster, Functional assessment of cell entry and receptor usage for SARS-CoV-2 and other lineage B betacoronaviruses. *Nat. Microbiol.* **5**, 562–569 (2020). doi:10.1038/s41564-020-0688-y Medline
10. M. Hoffmann, H. Kleine-Weber, S. Schroeder, N. Krüger, T. Herrler, S. Erichsen, T. S. Schiergens, G. Herrler, N. H. Wu, A. Nitsche, M. A. Müller, C. Drosten, S. Pöhlmann, SARS-CoV-2 cell entry depends on ACE2 and TMPRSS2 and is blocked by a clinically proven protease inhibitor. *Cell* **181**, 271–280.e8 (2020). doi:10.1016/j.cell.2020.02.052 Medline
11. W. Li, M. J. Moore, N. Vasilieva, J. Sui, S. K. Wong, M. A. Berne, M. Somasundaran, J. L. Sullivan, K. Luzuriaga, T. C. Greenough, H. Choe, M. Farzan, Angiotensin-converting enzyme 2 is a functional receptor for the SARS coronavirus. *Nature* **426**, 450–454 (2003). doi:10.1038/nature02145 Medline
12. F. Li, W. Li, M. Farzan, S. C. Harrison, Structure of SARS coronavirus spike receptor-binding domain complexed with receptor. *Science* **309**, 1864–1868 (2005). doi:10.1126/science.1116480 Medline
13. Q. Wang, Y. Zhang, L. Wu, S. Niu, C. Song, Z. Zhang, G. Lu, C. Qiao, Y. Hu, K.-Y. Yuen, Q. Wang, H. Zhou, J. Yan, J. Qi, Structural and functional basis of SARS-CoV-2 entry by using human ACE2. *Cell* **181**, 894–904.e9 (2020). doi:10.1016/j.cell.2020.03.045 Medline
14. R. Yan, Y. Zhang, Y. Li, L. Xia, Y. Guo, Q. Zhou, Structural basis for the recognition of SARS-CoV-2 by full-length human ACE2. *Science* **367**, 1444–1448 (2020). doi:10.1126/science.abb2762 Medline
15. J. Shang, G. Ye, K. Shi, Y. Wan, C. Luo, H. Aihara, Q. Geng, A. Auerbach, F. Li, Structural basis of receptor recognition by SARS-CoV-2. *Nature* **581**, 221–224 (2020). doi:10.1038/s41586-020-2179-y Medline
16. J. Lan, J. Ge, J. Yu, S. Shan, H. Zhou, S. Fan, Q. Zhang, X. Shi, Q. Wang, L. Zhang, X.

Wang, Structure of the SARS-CoV-2 spike receptor-binding domain bound to the ACE2 receptor. *Nature* **581**, 215–220 (2020). doi:10.1038/s41586-020-2180-5 Medline
17. L. Piccoli, Y. J. Park, M. A. Tortorici, N. Czudnochowski, A. C. Walls, M. Beltramello, C. Silacci-Fregni, D. Pinto, L. E. Rosen, J. E. Bowen, O. J. Acton, S. Jaconi, B. Guarino, A. Minola, F. Zatta, N. Sprugasci, J. Bassi, A. Peter, A. De Marco, J. C. Nix, F. Mele, S. Jovic, B. F. Rodriguez, S. V. Gupta, F. Jin, G. Piumatti, G. Lo Presti, A. F. Pellanda, M. Biggiogero, M. Tarkowski, M. S. Pizzuto, E. Cameroni, C. Havenar-Daughton, M. Smithey, D. Hong, V. Lepori, E. Albanese, A. Ceschi, E. Bernasconi, L. Elzi, P. Ferrari, C. Garzoni, A. Riva, G. Snell, F. Sallusto, K. Fink, H. W. Virgin, A. Lanzavecchia, D. Corti, D. Veesler, Mapping neutralizing and immunodominant sites on the SARS-CoV-2 spike receptor-binding domain by structure-guided high-resolution serology. *Cell* **183**, 1024–1042.e21 (2020). doi:10.1016/j.cell.2020.09.037 Medline
18. A. J. Greaney, A. N. Loes, L. E. Gentles, K. H. D. Crawford, T. N. Starr, K. D. Malone, H. Y. Chu, J. D. Bloom, Antibodies elicited by mRNA-1273 vaccination bind more broadly to the receptor binding domain than do those from SARS-CoV-2 infection. *Sci. Transl. Med.* **13**, eabi9915 (2021). doi:10.1126/scitranslmed.abi9915 Medline
19. M. A. Tortorici, N. Czudnochowski, T. N. Starr, R. Marzi, A. C. Walls, F. Zatta, J. E. Bowen, S. Jaconi, J. Di Iulio, Z. Wang, A. De Marco, S. K. Zepeda, D. Pinto, Z. Liu, M. Beltramello, I. Bartha, M. P. Housley, F. A. Lempp, L. E. Rosen, E. Dellota Jr., H. Kaiser, M. Montiel-Ruiz, J. Zhou, A. Addetia, B. Guarino, K. Culap, N. Sprugasci, C. Saliba, E. Vetti, I. Giacchetto-Sasselli, C. S. Fregni, R. Abdelnabi, S. C. Foo, C. Havenar-Daughton, M. A. Schmid, F. Benigni, E. Cameroni, J. Neyts, A. Telenti, H. W. Virgin, S. P. J. Whelan, G. Snell, J. D. Bloom, D. Corti, D. Veesler, M. S. Pizzuto, Broad sarbecovirus neutralization by a human monoclonal antibody. *Nature* **597**, 103–108 (2021). doi:10.1038/s41586-021-03817-4 Medline
20. T. N. Starr, N. Czudnochowski, Z. Liu, F. Zatta, Y.-J. Park, A. Addetia, D. Pinto, M. Beltramello, P. Hernandez, A. J. Greaney, R. Marzi, W. G. Glass, I. Zhang, A. S. Dingens, J. E. Bowen, M. A. Tortorici, A. C. Walls, J. A. Wojcechowskyj, A. De Marco, L. E. Rosen, J. Zhou, M. Montiel-Ruiz, H. Kaiser, J. R. Dillen, H. Tucker, J. Bassi, C. Silacci-Fregni, M. P. Housley, J. di Iulio, G. Lombardo, M. Agostini, N. Sprugasci, K. Culap, S. Jaconi, M. Meury, E. Dellota Jr., R. Abdelnabi, S. C. Foo, E. Cameroni, S. Stumpf, T. I. Croll, J. C. Nix, C. Havenar-Daughton, L. Piccoli, F. Benigni, J. Neyts, A. Telenti, F. A. Lempp, M. S. Pizzuto, J. D. Chodera, C. M. Hebner, H. W. Virgin, S. P. J. Whelan, D. Veesler, D. Corti, J. D. Bloom, G. Snell, SARS-CoV-2 RBD antibodies that maximize breadth and resistance to escape. *Nature* **597**, 97–102 (2021). doi:10.1038/s41586-021-03807-6 Medline
21. D. Pinto, Y. J. Park, M. Beltramello, A. C. Walls, M. A. Tortorici, S. Bianchi, S. Jaconi, K. Culap, F. Zatta, A. De Marco, A. Peter, B. Guarino, R. Spreafico, E. Cameroni, J. B. Case, R. E. Chen, C. Havenar-Daughton, G. Snell, A. Telenti, H. W. Virgin, A. Lanzavecchia, M. S. Diamond, K. Fink, D. Veesler, D. Corti, Cross-neutralization of SARS-CoV-2 by a human monoclonal SARS-CoV antibody. *Nature* **583**, 290–295 (2020). doi:10.1038/s41586-020-2349-y Medline
22. C. A. Jette, A. A. Cohen, P. N. P. Gnanapragasam, F. Muecksch, Y. E. Lee, K. E. Huey-Tubman, F. Schmidt, T. Hatziioannou, P. D. Bieniasz, M. C. Nussenzweig, A. P. West Jr., J. R. Keeffe, P. J. Bjorkman, C. O. Barnes, Broad cross-reactivity across sarbecoviruses exhibited by a subset of COVID-19 donor-derived neutralizing antibodies. *Cell Rep.* **36**, 109760 (2021). doi:10.1016/j.celrep.2021.109760
23. D. R. Martinez, A. Schaefer, S. Gobeil, D. Li, G. De la Cruz, R. Parks, X. Lu, M. Barr, K. Manne, K. Mansouri, R. J. Edwards, B. Yount, K. Anasti, S. A. Montgomery, S. Shen, T. Zhou, P. D. Kwong, B. S. Graham, J. R. Mascola, D. C. Montefiori, M. Alam, G. D. Sempowski, K. Wiehe, K. O. Saunders, P. Acharya, B. F. Haynes, R. S. Baric, A broadly neutralizing antibody protects against SARS-CoV, pre-emergent bat CoVs, and SARS-CoV-2 variants in mice. bioRxiv 2021.04.27.441655 [Preprint] (2021). https://doi.org/10.1101/2021.04.27.441655.
24. A. Z. Wec, D. Wrapp, A. S. Herbert, D. P. Maurer, D. Haslwanter, M. Sakharkar, R. K. Jangra, M. E. Dieterle, A. Lilov, D. Huang, L. V. Tse, N. V. Johnson, C. L. Hsieh, N. Wang, J. H. Nett, E. Champney, I. Burnina, M. Brown, S. Lin, M. Sinclair, C. Johnson, S. Pudi, R. Bortz 3rd, A. S. Wirchnianski, E. Laudermilch, C. Florez, J. M. Fels, C. M. O'Brien, B. S. Graham, D. Nemazee, D. R. Burton, R. S. Baric, J. E. Voss, K. Chandran, J. M. Dye, J. S. McLellan, L. M. Walker, Broad neutralization of SARS-related viruses by human monoclonal antibodies. *Science* **369**, 731–736 (2020). doi:10.1126/science.abc7424 Medline
25. C. G. Rappazzo, L. V. Tse, C. I. Kaku, D. Wrapp, M. Sakharkar, D. Huang, L. M. Deveau, T. J. Yockachonis, A. S. Herbert, M. B. Battles, C. M. O'Brien, M. E. Brown, J. C. Geoghegan, J. Belk, L. Peng, L. Yang, Y. Hou, T. D. Scobey, D. R. Burton, D. Nemazee, J. M. Dye, J. E. Voss, B. M. Gunn, J. S. McLellan, R. S. Baric, L. E. Gralinski, L. M. Walker, Broad and potent activity against SARS-like viruses by an engineered human monoclonal antibody. *Science* **371**, 823–829 (2021). doi:10.1126/science.abf4830 Medline
26. D. Corti, L. A. Purcell, G. Snell, D. Veesler, Tackling COVID-19 with neutralizing monoclonal antibodies. *Cell* **184**, 3086–3108 (2021). doi:10.1016/j.cell.2021.05.005 Medline
27. M. McCallum, J. Bassi, A. De Marco, A. Chen, A. C. Walls, J. Di Iulio, M. A. Tortorici, M.-J. Navarro, C. Silacci-Fregni, C. Saliba, K. R. Sprouse, M. Agostini, D. Pinto, K. Culap, S. Bianchi, S. Jaconi, E. Cameroni, J. E. Bowen, S. W. Tilles, M. S. Pizzuto, S. B. Guastalla, G. Bona, A. F. Pellanda, C. Garzoni, W. C. Van Voorhis, L. E. Rosen, G. Snell, A. Telenti, H. W. Virgin, L. Piccoli, D. Corti, D. Veesler, SARS-CoV-2 immune evasion by the B.1.427/B.1.429 variant of concern. *Science* **373**, 648–654 (2021). doi:10.1126/science.abi7994 Medline
28. M. McCallum, A. C. Walls, K. R. Sprouse, J. E. Bowen, L. E. Rosen, H. V. Dang, A. De Marco, N. Franko, S. W. Tilles, J. Logue, M. C. Miranda, M. Ahlrichs, L. Carter, G. Snell, M. S. Pizzuto, H. Y. Chu, W. C. Van Voorhis, D. Corti, D. Veesler, Molecular basis of immune evasion by the Delta and Kappa SARS-CoV-2 variants. *Science* **374**, 1621–1626 (2021). doi:10.1126/science.abl8506 Medline
29. P. Mlcochova, S. A. Kemp, M. S. Dhar, G. Papa, B. Meng, I. A. T. M. Ferreira, R. Datir, D. A. Collier, A. Albecka, S. Singh, R. Pandey, J. Brown, J. Zhou, N. Goonawardane, S. Mishra, C. Whittaker, T. Mellan, R. Marwal, M. Datta, S. Sengupta, K. Ponnusamy, V. S. Radhakrishnan, A. Abdullahi, O. Charles, P. Chattopadhyay, P. Devi, D. Caputo, T. Peacock, C. Wattal, N. Goel, A. Satwik, R. Vaishya, M. Agarwal, A. Mavousian, J. H. Lee, J. Bassi, C. Silacci-Fegni, C. Saliba, D. Pinto, T. Irie, I. Yoshida, W. L. Hamilton, K. Sato, S. Bhatt, S. Flaxman, L. C. James, D. Corti, L. Piccoli, W. S. Barclay, P. Rakshit, A. Agrawal, R. K. Gupta; Indian SARS-CoV-2 Genomics Consortium (INSACOG); Genotype to Phenotype Japan (G2P-Japan) Consortium; CITIID-NIHR BioResource COVID-19 Collaboration, SARS-CoV-2 B.1.617.2 Delta variant replication and immune evasion. *Nature* **599**, 114–119 (2021). doi:10.1038/s41586-021-03944-y Medline
30. T. N. Starr, A. J. Greaney, S. K. Hilton, D. Ellis, K. H. D. Crawford, A. S. Dingens, M. J. Navarro, J. E. Bowen, M. A. Tortorici, A. C. Walls, N. P. King, D. Veesler, J. D. Bloom, Deep mutational scanning of SARS-CoV-2 receptor binding domain reveals constraints on folding and ACE2 binding. *Cell* **182**, 1295–1310.e20 (2020). doi:10.1016/j.cell.2020.08.012 Medline
31. T. N. Starr, A. J. Greaney, A. S. Dingens, J. D. Bloom, Complete map of SARS-CoV-2 RBD mutations that escape the monoclonal antibody LY-CoV555 and its cocktail with LY-CoV016. *Cell Rep. Med.* **2**, 100255 (2021). doi:10.1016/j.xcrm.2021.100255 Medline
32. T. N. Starr, A. J. Greaney, A. Addetia, W. W. Hannon, M. C. Choudhary, A. S. Dingens, J. Z. Li, J. D. Bloom, Prospective mapping of viral mutations that escape antibodies used to treat COVID-19. *Science* **371**, 850–854 (2021). doi:10.1126/science.abf9302 Medline
33. M. A. Tortorici, M. Beltramello, F. A. Lempp, D. Pinto, H. V. Dang, L. E. Rosen, M. McCallum, J. Bowen, A. Minola, S. Jaconi, F. Zatta, A. De Marco, B. Guarino, S. Bianchi, E. J. Lauron, H. Tucker, J. Zhou, A. Peter, C. Havenar-Daughton, J. A. Wojcechowskyj, J. B. Case, R. E. Chen, H. Kaiser, M. Montiel-Ruiz, M. Meury, N. Czudnochowski, R. Spreafico, J. Dillen, C. Ng, N. Sprugasci, K. Culap, F. Benigni, R. Abdelnabi, S. C. Foo, M. A. Schmid, E. Cameroni, A. Riva, A. Gabrieli, M. Galli, M. S. Pizzuto, J. Neyts, M. S. Diamond, H. W. Virgin, G. Snell, D. Corti, K. Fink, D. Veesler, Ultrapotent human antibodies protect against SARS-CoV-2 challenge via multiple mechanisms. *Science* **370**, 950–957 (2020). doi:10.1126/science.abe3354 Medline
34. T. N. Starr, S. K. Zepeda, A. C. Walls, A. J. Greaney, D. Veesler, J. D. Bloom, ACE2 binding is an ancestral and evolvable trait of sarbecoviruses. bioRxiv 2021.07.17.452804 [Preprint] (2021). https://doi.org/10.1101/2021.07.17.452804.
35. J. B. Case, P. W. Rothlauf, R. E. Chen, Z. Liu, H. Zhao, A. S. Kim, L. M. Bloyet, Q. Zeng, S. Tahan, L. Droit, M. X. G. Ilagan, M. A. Tartell, G. Amarasinghe, J. P. Henderson, S. Miersch, M. Ustav, S. Sidhu, H. W. Virgin, D. Wang, S. Ding, D. Corti, E. S. Theel, D. H. Fremont, M. S. Diamond, S. P. J. Whelan, Neutralizing antibody

and soluble ACE2 inhibition of a replication-competent VSV-SARS-CoV-2 and a clinical isolate of SARS-CoV-2. *Cell Host Microbe* **28**, 475–485.e5 (2020). doi:10.1016/j.chom.2020.06.021 Medline
36. A. C. Walls, X. Xiong, Y. J. Park, M. A. Tortorici, J. Snijder, J. Quispe, E. Cameroni, R. Gopal, M. Dai, A. Lanzavecchia, M. Zambon, F. A. Rey, D. Corti, D. Veesler, Unexpected receptor functional mimicry elucidates activation of coronavirus fusion. *Cell* **176**, 1026–1039.e15 (2019). doi:10.1016/j.cell.2018.12.028 Medline
37. F. A. Lempp, L. B. Soriaga, M. Montiel-Ruiz, F. Benigni, J. Noack, Y.-J. Park, S. Bianchi, A. C. Walls, J. E. Bowen, J. Zhou, H. Kaiser, A. Joshi, M. Agostini, M. Meury, E. Dellota Jr., S. Jaconi, E. Cameroni, J. Martinez-Picado, J. Vergara-Alert, N. Izquierdo-Useros, H. W. Virgin, A. Lanzavecchia, D. Veesler, L. A. Purcell, A. Telenti, D. Corti, Lectins enhance SARS-CoV-2 infection and influence neutralizing antibodies. *Nature* **598**, 342–347 (2021). doi:10.1038/s41586-021-03925-1 Medline
38. J. Huo, Y. Zhao, J. Ren, D. Zhou, H. M. E. Duyvesteyn, H. M. Ginn, L. Carrique, T. Malinauskas, R. R. Ruza, P. N. M. Shah, T. K. Tan, P. Rijal, N. Coombes, K. R. Bewley, J. A. Tree, J. Radecke, N. G. Paterson, P. Supasa, J. Mongkolsapaya, G. R. Screaton, M. Carroll, A. Townsend, E. E. Fry, R. J. Owens, D. I. Stuart, Neutralization of SARS-CoV-2 by destruction of the prefusion spike. *Cell Host Microbe* **28**, 445–454.e6 (2020). doi:10.1016/j.chom.2020.06.010 Medline
39. R. Abdelnabi, R. Boudewijns, C. S. Foo, L. Seldeslachts, L. Sanchez-Felipe, X. Zhang, L. Delang, P. Maes, S. J. F. Kaptein, B. Weynand, G. Vande Velde, J. Neyts, K. Dallmeier, Comparing infectivity and virulence of emerging SARS-CoV-2 variants in Syrian hamsters. *EBioMedicine* **68**, 103403 (2021). doi:10.1016/j.ebiom.2021.103403 Medline
40. R. Boudewijns, H. J. Thibaut, S. J. F. Kaptein, R. Li, V. Vergote, L. Seldeslachts, J. Van Weyenbergh, C. De Keyzer, L. Bervoets, S. Sharma, L. Liesenborghs, J. Ma, S. Jansen, D. Van Looveren, T. Vercruysse, X. Wang, D. Jochmans, E. Martens, K. Roose, D. De Vlieger, B. Schepens, T. Van Buyten, S. Jacobs, Y. Liu, J. Martí-Carreras, B. Vanmechelen, T. Wawina-Bokalanga, L. Delang, J. Rocha-Pereira, L. Coelmont, W. Chiu, P. Leyssen, E. Heylen, D. Schols, L. Wang, L. Close, J. Matthijnssens, M. Van Ranst, V. Compernolle, G. Schramm, K. Van Laere, X. Saelens, N. Callewaert, G. Opdenakker, P. Maes, B. Weynand, C. Cawthorne, G. Vande Velde, Z. Wang, J. Neyts, K. Dallmeier, STAT2 signaling restricts viral dissemination but drives severe pneumonia in SARS-CoV-2 infected hamsters. *Nat. Commun.* **11**, 5838 (2020). doi:10.1038/s41467-020-19684-y Medline
41. W. Dejnirattisai, D. Zhou, H. M. Ginn, H. M. E. Duyvesteyn, P. Supasa, J. B. Case, Y. Zhao, T. S. Walter, A. J. Mentzer, C. Liu, B. Wang, G. C. Paesen, J. Slon-Campos, C. López-Camacho, N. M. Kafai, A. L. Bailey, R. E. Chen, B. Ying, C. Thompson, J. Bolton, A. Fyfe, S. Gupta, T. K. Tan, J. Gilbert-Jaramillo, W. James, M. Knight, M. W. Carroll, D. Skelly, C. Dold, Y. Peng, R. Levin, T. Dong, A. J. Pollard, J. C. Knight, P. Klenerman, N. Temperton, D. R. Hall, M. A. Williams, N. G. Paterson, F. K. R. Bertram, C. A. Siebert, D. K. Clare, A. Howe, J. Radecke, Y. Song, A. R. Townsend, K.-Y. A. Huang, E. E. Fry, J. Mongkolsapaya, M. S. Diamond, J. Ren, D. I. Stuart, G. R. Screaton, The antigenic anatomy of SARS-CoV-2 receptor binding domain. *Cell* **184**, 2183–2200.e22 (2021). doi:10.1016/j.cell.2021.02.032 Medline
42. P. S. Arunachalam, A. C. Walls, N. Golden, C. Atyeo, S. Fischinger, C. Li, P. Aye, M. J. Navarro, L. Lai, V. V. Edara, K. Röltgen, K. Rogers, L. Shirreff, D. E. Ferrell, S. Wrenn, D. Pettie, J. C. Kraft, M. C. Miranda, E. Kepl, C. Sydeman, N. Brunette, M. Murphy, B. Fiala, L. Carter, A. G. White, M. Trisal, C.-L. Hsieh, K. Russell-Lodrigue, C. Monjure, J. Dufour, S. Spencer, L. Doyle-Meyers, R. P. Bohm, N. J. Maness, C. Roy, J. A. Plante, K. S. Plante, A. Zhu, M. J. Gorman, S. Shin, X. Shen, J. Fontenot, S. Gupta, D. T. O'Hagan, R. Van Der Most, R. Rappuoli, R. L. Coffman, D. Novack, J. S. McLellan, S. Subramaniam, D. Montefiori, S. D. Boyd, J. A. L. Flynn, G. Alter, F. Villinger, H. Kleanthous, J. Rappaport, M. S. Suthar, N. P. King, D. Veesler, B. Pulendran, Adjuvanting a subunit COVID-19 vaccine to induce protective immunity. *Nature* **594**, 253–258 (2021). doi:10.1038/s41586-021-03530-2 Medline
43. A. C. Walls, B. Fiala, A. Schäfer, S. Wrenn, M. N. Pham, M. Murphy, L. V. Tse, L. Shehata, M. A. O'Connor, C. Chen, M. J. Navarro, M. C. Miranda, D. Pettie, R. Ravichandran, J. C. Kraft, C. Ogohara, A. Palser, S. Chalk, E. C. Lee, K. Guerriero, E. Kepl, C. M. Chow, C. Sydeman, E. A. Hodge, B. Brown, J. T. Fuller, K. H. Dinnon 3rd, L. E. Gralinski, S. R. Leist, K. L. Gully, T. B. Lewis, M. Guttman, H. Y. Chu, K. K. Lee, D. H. Fuller, R. S. Baric, P. Kellam, L. Carter, M. Pepper, T. P. Sheahan, D. Veesler, N. P. King, Elicitation of potent neutralizing antibody responses by designed protein nanoparticle vaccines for SARS-CoV-2. *Cell* **183**, 1367–1382.e17 (2020). doi:10.1016/j.cell.2020.10.043 Medline
44. A. C. Walls, M. C. Miranda, A. Schäfer, M. N. Pham, A. Greaney, P. S. Arunachalam, M.-J. Navarro, M. A. Tortorici, K. Rogers, M. A. O'Connor, L. Shirreff, D. E. Ferrell, J. Bowen, N. Brunette, E. Kepl, S. K. Zepeda, T. Starr, C.-L. Hsieh, B. Fiala, S. Wrenn, D. Pettie, C. Sydeman, K. R. Sprouse, M. Johnson, A. Blackstone, R. Ravichandran, C. Ogohara, L. Carter, S. W. Tilles, R. Rappuoli, S. R. Leist, D. R. Martinez, M. Clark, R. Tisch, D. T. O'Hagan, R. Van Der Most, W. C. Van Voorhis, D. Corti, J. S. McLellan, H. Kleanthous, T. P. Sheahan, K. D. Smith, D. H. Fuller, F. Villinger, J. Bloom, B. Pulendran, R. S. Baric, N. P. King, D. Veesler, Elicitation of broadly protective sarbecovirus immunity by receptor-binding domain nanoparticle vaccines. *Cell* **184**, 5432–5447.e16 (2021). doi:10.1016/j.cell.2021.09.015 Medline
45. K. O. Saunders, E. Lee, R. Parks, D. R. Martinez, D. Li, H. Chen, R. J. Edwards, S. Gobeil, M. Barr, K. Mansouri, S. M. Alam, L. L. Sutherland, F. Cai, A. M. Sanzone, M. Berry, K. Manne, K. W. Bock, M. Minai, B. M. Nagata, A. B. Kapingidza, M. Azoitei, L. V. Tse, T. D. Scobey, R. L. Spreng, R. W. Rountree, C. T. DeMarco, T. N. Denny, C. W. Woods, E. W. Petzold, J. Tang, T. H. Oguin 3rd, G. D. Sempowski, M. Gagne, D. C. Douek, M. A. Tomai, C. B. Fox, R. Seder, K. Wiehe, D. Weissman, N. Pardi, H. Golding, S. Khurana, P. Acharya, H. Andersen, M. G. Lewis, I. N. Moore, D. C. Montefiori, R. S. Baric, B. F. Haynes, Neutralizing antibody vaccine for pandemic and pre-emergent coronaviruses. *Nature* **594**, 553–559 (2021). doi:10.1038/s41586-021-03594-0 Medline
46. D. R. Martinez, A. Schäfer, S. R. Leist, G. De la Cruz, A. West, E. N. Atochina-Vasserman, L. C. Lindesmith, N. Pardi, R. Parks, M. Barr, D. Li, B. Yount, K. O. Saunders, D. Weissman, B. F. Haynes, S. A. Montgomery, R. S. Baric, Chimeric spike mRNA vaccines protect against Sarbecovirus challenge in mice. *Science* **373**, 991–998 (2021). doi:10.1126/science.abi4506 Medline
47. D. Pinto, M. M. Sauer, N. Czudnochowski, J. S. Low, M. A. Tortorici, M. P. Housley, J. Noack, A. C. Walls, J. E. Bowen, B. Guarino, L. E. Rosen, J. di Iulio, J. Jerak, H. Kaiser, S. Islam, S. Jaconi, N. Sprugasci, K. Culap, R. Abdelnabi, C. Foo, L. Coelmont, I. Bartha, S. Bianchi, C. Silacci-Fregni, J. Bassi, R. Marzi, E. Vetti, A. Cassotta, A. Ceschi, P. Ferrari, P. E. Cippà, O. Giannini, S. Ceruti, C. Garzoni, A. Riva, F. Benigni, E. Cameroni, L. Piccoli, M. S. Pizzuto, M. Smithey, D. Hong, A. Telenti, F. A. Lempp, J. Neyts, C. Havenar-Daughton, A. Lanzavecchia, F. Sallusto, G. Snell, H. W. Virgin, M. Beltramello, D. Corti, D. Veesler, Broad betacoronavirus neutralization by a stem helix-specific human antibody. *Science* **373**, 1109–1116 (2021). doi:10.1126/science.abj3321 Medline
48. M. M. Sauer, M. A. Tortorici, Y.-J. Park, A. C. Walls, L. Homad, O. J. Acton, J. E. Bowen, C. Wang, X. Xiong, W. de van der Schueren, J. Quispe, B. G. Hoffstrom, B.-J. Bosch, A. T. McGuire, D. Veesler, Structural basis for broad coronavirus neutralization. *Nat. Struct. Mol. Biol.* **28**, 478–486 (2021). doi:10.1038/s41594-021-00596-4 Medline
49. G. Song, W.-T. He, S. Callaghan, F. Anzanello, D. Huang, J. Ricketts, J. L. Torres, N. Beutler, L. Peng, S. Vargas, J. Cassell, M. Parren, L. Yang, C. Ignacio, D. M. Smith, J. E. Voss, D. Nemazee, A. B. Ward, T. Rogers, D. R. Burton, R. Andrabi, Cross-reactive serum and memory B-cell responses to spike protein in SARS-CoV-2 and endemic coronavirus infection. *Nat. Commun.* **12**, 2938 (2021). doi:10.1038/s41467-021-23074-3 Medline
50. P. Zhou, M. Yuan, G. Song, N. Beutler, N. Shaabani, D. Huang, W.-T. He, X. Zhu, S. Callaghan, P. Yong, F. Anzanello, L. Peng, J. Ricketts, M. Parren, E. Garcia, S. A. Rawlings, D. M. Smith, D. Nemazee, J. R. Teijaro, T. F. Rogers, I. A. Wilson, D. R. Burton, R. Andrabi, A protective broadly cross-reactive human antibody defines a conserved site of vulnerability on beta-coronavirus spikes. bioRxiv 2021.03.30.437769 [Preprint] (2021). https://doi.org/10.1101/2021.03.30.437769.
51. C. Wang, R. van Haperen, J. Gutiérrez-Álvarez, W. Li, N. M. A. Okba, I. Albulescu, I. Widjaja, B. van Dieren, R. Fernandez-Delgado, I. Sola, D. L. Hurdiss, O. Daramola, F. Grosveld, F. J. M. van Kuppeveld, B. L. Haagmans, L. Enjuanes, D. Drabek, B.-J. Bosch, A conserved immunogenic and vulnerable site on the coronavirus spike protein delineated by cross-reactive monoclonal antibodies. *Nat. Commun.* **12**, 1715 (2021). doi:10.1038/s41467-021-21968-w Medline
52. C.-L. Hsieh, A. P. Werner, S. R. Leist, L. J. Stevens, E. Falconer, J. A. Goldsmith, C.-W. Chou, O. M. Abiona, A. West, K. Westendorf, K. Muthuraman, E. J. Fritch, K. H. Dinnon 3rd, A. Schäfer, M. R. Denison, J. D. Chappell, R. S. Baric, B. S. Graham, K.

S. Corbett, J. S. McLellan, Stabilized coronavirus spike stem elicits a broadly protective antibody. *Cell Rep.* **37**, 109929 (2021). doi:10.1016/j.celrep.2021.109929 Medline

53. A. L. Cathcart, C. Havenar-Daughton, F. A. Lempp, D. Ma, M. Schmid, M. L. Agostini, B. Guarino, J. Di iulio, L. Rosen, H. Tucker, J. Dillen, S. Subramanian, B. Sloan, S. Bianchi, J. Wojcechowskyj, J. Zhou, H. Kaiser, A. Chase, M. Montiel-Ruiz, N. Czudnochowski, E. Cameroni, S. Ledoux, C. Colas, L. Soriaga, A. Telenti, S. Hwang, G. Snell, H. W. Virgin, D. Corti, C. M. Hebner, The dual function monoclonal antibodies VIR-7831 and VIR-7832 demonstrate potent in vitro and in vivo activity against SARS-CoV-2. bioRxiv 2021.03.09.434607 [Preprint] (2021). https://doi.org/10.1101/2021.03.09.434607.
54. C. L. Hsieh, J. A. Goldsmith, J. M. Schaub, A. M. DiVenere, H. C. Kuo, K. Javanmardi, K. C. Le, D. Wrapp, A. G. Lee, Y. Liu, C. W. Chou, P. O. Byrne, C. K. Hjorth, N. V. Johnson, J. Ludes-Meyers, A. W. Nguyen, J. Park, N. Wang, D. Amengor, J. J. Lavinder, G. C. Ippolito, J. A. Maynard, I. J. Finkelstein, J. S. McLellan, Structure-based design of prefusion-stabilized SARS-CoV-2 spikes. *Science* **369**, 1501–1505 (2020). doi:10.1126/science.abd0826 Medline
55. D. Pinto, C. Fenwick, C. Caillat, C. Silacci, S. Guseva, F. Dehez, C. Chipot, S. Barbieri, A. Minola, D. Jarrossay, G. D. Tomaras, X. Shen, A. Riva, M. Tarkowski, O. Schwartz, T. Bruel, J. Dufloo, M. S. Seaman, D. C. Montefiori, A. Lanzavecchia, D. Corti, G. Pantaleo, W. Weissenhorn, Structural basis for broad HIV-1 neutralization by the MPER-specific human broadly neutralizing antibody LN01. *Cell Host Microbe* **26**, 623–637.e8 (2019). doi:10.1016/j.chom.2019.09.016 Medline
56. Y. Kaname, H. Tani, C. Kataoka, M. Shiokawa, S. Taguwa, T. Abe, K. Moriishi, T. Kinoshita, Y. Matsuura, Acquisition of complement resistance through incorporation of CD55/decay-accelerating factor into viral particles bearing baculovirus GP64. *J. Virol.* **84**, 3210–3219 (2010). doi:10.1128/JVI.02519-09 Medline
57. A. J. Greaney, T. N. Starr, P. Gilchuk, S. J. Zost, E. Binshtein, A. N. Loes, S. K. Hilton, J. Huddleston, R. Eguia, K. H. D. Crawford, A. S. Dingens, R. S. Nargi, R. E. Sutton, N. Suryadevara, P. W. Rothlauf, Z. Liu, S. P. J. Whelan, R. H. Carnahan, J. E. Crowe Jr., J. D. Bloom, Complete mapping of mutations to the SARS-CoV-2 spike receptor-binding domain that escape antibody recognition. *Cell Host Microbe* **29**, 44–57.e9 (2021). doi:10.1016/j.chom.2020.11.007 Medline
58. C. Suloway, J. Pulokas, D. Fellmann, A. Cheng, F. Guerra, J. Quispe, S. Stagg, C. S. Potter, B. Carragher, Automated molecular microscopy: The new Leginon system. *J. Struct. Biol.* **151**, 41–60 (2005). doi:10.1016/j.jsb.2005.03.010 Medline
59. D. Tegunov, P. Cramer, Real-time cryo-electron microscopy data preprocessing with Warp. *Nat. Methods* **16**, 1146–1152 (2019). doi:10.1038/s41592-019-0580-y Medline
60. A. Punjani, J. L. Rubinstein, D. J. Fleet, M. A. Brubaker, cryoSPARC: Algorithms for rapid unsupervised cryo-EM structure determination. *Nat. Methods* **14**, 290–296 (2017). doi:10.1038/nmeth.4169 Medline
61. J. Zivanov, T. Nakane, B. O. Forsberg, D. Kimanius, W. J. Hagen, E. Lindahl, S. H. Scheres, New tools for automated high-resolution cryo-EM structure determination in RELION-3. *eLife* **7**, e42166 (2018). doi:10.7554/eLife.42166 Medline
62. A. Punjani, H. Zhang, D. J. Fleet, Non-uniform refinement: Adaptive regularization improves single-particle cryo-EM reconstruction. *Nat. Methods* **17**, 1214–1221 (2020). doi:10.1038/s41592-020-00990-8 Medline
63. J. Zivanov, T. Nakane, S. H. W. Scheres, A Bayesian approach to beam-induced motion correction in cryo-EM single-particle analysis. *IUCrJ* **6**, 5–17 (2019). doi:10.1107/S205225251801463X Medline
64. S. Chen, G. McMullan, A. R. Faruqi, G. N. Murshudov, J. M. Short, S. H. Scheres, R. Henderson, High-resolution noise substitution to measure overfitting and validate resolution in 3D structure determination by single particle electron cryomicroscopy. *Ultramicroscopy* **135**, 24–35 (2013). doi:10.1016/j.ultramic.2013.06.004 Medline
65. P. B. Rosenthal, R. Henderson, Optimal determination of particle orientation, absolute hand, and contrast loss in single-particle electron cryomicroscopy. *J. Mol. Biol.* **333**, 721–745 (2003). doi:10.1016/j.jmb.2003.07.013 Medline
66. E. F. Pettersen, T. D. Goddard, C. C. Huang, G. S. Couch, D. M. Greenblatt, E. C. Meng, T. E. Ferrin, UCSF Chimera—A visualization system for exploratory research and analysis. *J. Comput. Chem.* **25**, 1605–1612 (2004). doi:10.1002/jcc.20084 Medline
67. P. Emsley, B. Lohkamp, W. G. Scott, K. Cowtan, Features and development of *Coot*. *Acta Crystallogr. D Biol. Crystallogr.* **66**, 486–501 (2010). doi:10.1107/S0907444910007493 Medline
68. B. Frenz, S. Rämisch, A. J. Borst, A. C. Walls, J. Adolf-Bryfogle, W. R. Schief, D. Veesler, F. DiMaio, Automatically fixing errors in glycoprotein structures with Rosetta. *Structure* **27**, 134–139.e3 (2019). doi:10.1016/j.str.2018.09.006 Medline
69. R. Y. Wang, Y. Song, B. A. Barad, Y. Cheng, J. S. Fraser, F. DiMaio, Automated structure refinement of macromolecular assemblies from cryo-EM maps using Rosetta. *eLife* **5**, e17219 (2016). doi:10.7554/eLife.17219 Medline
70. V. B. Chen, W. B. Arendall 3rd, J. J. Headd, D. A. Keedy, R. M. Immormino, G. J. Kapral, L. W. Murray, J. S. Richardson, D. C. Richardson, *MolProbity*: All-atom structure validation for macromolecular crystallography. *Acta Crystallogr. D Biol. Crystallogr.* **66**, 12–21 (2010). doi:10.1107/S0907444909042073 Medline
71. B. A. Barad, N. Echols, R. Y. Wang, Y. Cheng, F. DiMaio, P. D. Adams, J. S. Fraser, EMRinger: Side chain-directed model and map validation for 3D cryo-electron microscopy. *Nat. Methods* **12**, 943–946 (2015). doi:10.1038/nmeth.3541 Medline
72. D. Liebschner, P. V. Afonine, M. L. Baker, G. Bunkóczi, V. B. Chen, T. I. Croll, B. Hintze, L. W. Hung, S. Jain, A. J. McCoy, N. W. Moriarty, R. D. Oeffner, B. K. Poon, M. G. Prisant, R. J. Read, J. S. Richardson, D. C. Richardson, M. D. Sammito, O. V. Sobolev, D. H. Stockwell, T. C. Terwilliger, A. G. Urzhumtsev, L. L. Videau, C. J. Williams, P. D. Adams, Macromolecular structure determination using X-rays, neutrons and electrons: Recent developments in *Phenix*. *Acta Crystallogr. D Struct. Biol.* **75**, 861–877 (2019). doi:10.1107/S2059798319011471 Medline
73. J. Agirre, J. Iglesias-Fernández, C. Rovira, G. J. Davies, K. S. Wilson, K. D. Cowtan, Privateer: Software for the conformational validation of carbohydrate structures. *Nat. Struct. Mol. Biol.* **22**, 833–834 (2015). doi:10.1038/nsmb.3115 Medline
74. T. D. Goddard, C. C. Huang, E. C. Meng, E. F. Pettersen, G. S. Couch, J. H. Morris, T. E. Ferrin, UCSF ChimeraX: Meeting modern challenges in visualization and analysis. *Protein Sci.* **27**, 14–25 (2018). doi:10.1002/pro.3235 Medline
75. L. J. Reed, H. Muench, A simple method of estimating fifty per cent endpoints. *Am. J. Epidemiol.* **27**, 493–497 (1938). doi:10.1093/oxfordjournals.aje.a118408
76. K. H. D. Crawford, R. Eguia, A. S. Dingens, A. N. Loes, K. D. Malone, C. R. Wolf, H. Y. Chu, M. A. Tortorici, D. Veesler, M. Murphy, D. Pettie, N. P. King, A. B. Balazs, J. D. Bloom, Protocol and reagents for pseudotyping lentiviral particles with SARS-CoV-2 spike protein for neutralization assays. *Viruses* **12**, 513 (2020). doi:10.3390/v12050513 Medline

ACKNOWLEDGMENTS

The authors thank Cindy Castado and Normand Blais (GSK Vaccines) for their help in the selection of the genetically divergent sarbecoviruses used in this study and Hideki Tani (University of Toyama) for providing the reagents necessary for preparing VSV pseudotyped viruses. **Funding:** This study was supported by the National Institute of Allergy and Infectious Diseases (DP1AI158186 and HHSN272201700059C to D.V.), National Institute of General Medical Sciences (5T32GM008268 to S.K.Z.), a Pew Biomedical Scholars Award (D.V.), an Investigators in the Pathogenesis of Infectious Disease Awards from the Burroughs Wellcome Fund (D.V.), Fast Grants (D.V.), the University of Washington Arnold and Mabel Beckman cryoEM center and the National Institute of Health grant S10OD032290 (to D.V.). J.D.B. and D.V. are investigators of the Howard Hughes Medical Institute. **Author contributions:** Y.J.P., A.D.M., T.N.S., Z.L., D.P., J.D.B., D.C., M.S.P. and D.V. designed the experiments; A.D.M., D.P., A.C.W., S.K.Z., K.S.S. F.Z., M.G., J.N. and F.A.L. isolated mAb and performed binding, neutralization assays and biolayer interferometry measurements; A.D.M. and D.P. performed ACE2 binding inhibition and S_1 shedding assays; B.G. evaluated effector functions; T.N.S. and J.D.B. performed deep-mutational scanning; Z.L. and S.P.J.W. performed mutant selection and fitness assays; R.A., S.-Y.C.F., F.B., and J.N., D.C. and M.S.P. performed hamster model experiments and data analysis; Y.J.P. carried out cryoEM specimen preparation, data collection and processing. Y.J.P. and D.V. built and refined the atomic models. S.K.Z., A.J. and J.E.B. purified recombinant glycoproteins. Y.J.P., A.D.M., T.N.S., Z.L., D.P., J.D.B., D.C., M.S.P. and D.V. analyzed the data; Y.J.P., A.D.M., D.C., M.S.P. and D.V. wrote the manuscript with input from all authors; F.A.L., F.B., G.S., J.N., S.P.J.W., H.W.V., J.D.B., D.C., M.S.P. and D.V. supervised the project. **Competing interests:** A.D.M., D.P., F.Z., M.G., B.G., J.N., F.A.L., F.B., G.S., H.W.V., D.C., M.S.P. are employees of Vir

Biotechnology Inc. and may hold shares in Vir Biotechnology Inc. D.C. is currently listed as an inventor on multiple patent applications, which disclose the subject matter described in this manuscript. J.D.B. is an inventor on patents licensed by Fred Hutchinson cancer research center related to deep mutational scanning of viral proteins. The Veesler and Neyts laboratories have received sponsored research agreements from Vir Biotechnology Inc. H.W.V. is a founder of PierianDx and Casma Therapeutics. Neither company provided funding for this work or is performing related work. J.D.B. consults for Moderna, Oncorus and Flagship Labs 77. **Data and materials availability:** The cryoEM map and coordinates have been deposited to the Electron Microscopy Databank and Protein Data Bank with the following accession numbers; SARS-CoV-2 S/S2K146 (3RBDs open) EMD-25785; SARS-CoV-2 S/S2K146 (2RBDs open) PDB 7TAT, EMD-25784; SARS-CoV-2 S RBD/S2K146 (Local refinement) PDB 7TAS, EMD-25783. Materials generated in this study will be made available on request, but we may require a completed materials transfer agreement signed with Vir Biotechnology or the University of Washington. This work is licensed under a Creative Commons Attribution 4.0 International (CC BY 4.0) license, which permits unrestricted use, distribution, and reproduction in any medium, provided the original work is properly cited. To view a copy of this license, visit https://creativecommons.org/licenses/by/4.0/. This license does not apply to figures/photos/artwork or other content included in the article that is credited to a third party; obtain authorization from the rights holder before using such material.

SUPPLEMENTARY MATERIALS

science.org/doi/10.1126/science.abm8143
Materials and Methods
Figs. S1 to S10
Tables S1 and S2
References (*54*–*76*)
MDAR Reproducibility Checklist

13 October 2021; accepted 22 December 2021
Published online 6 January 2022
10.1126/science.abm8143

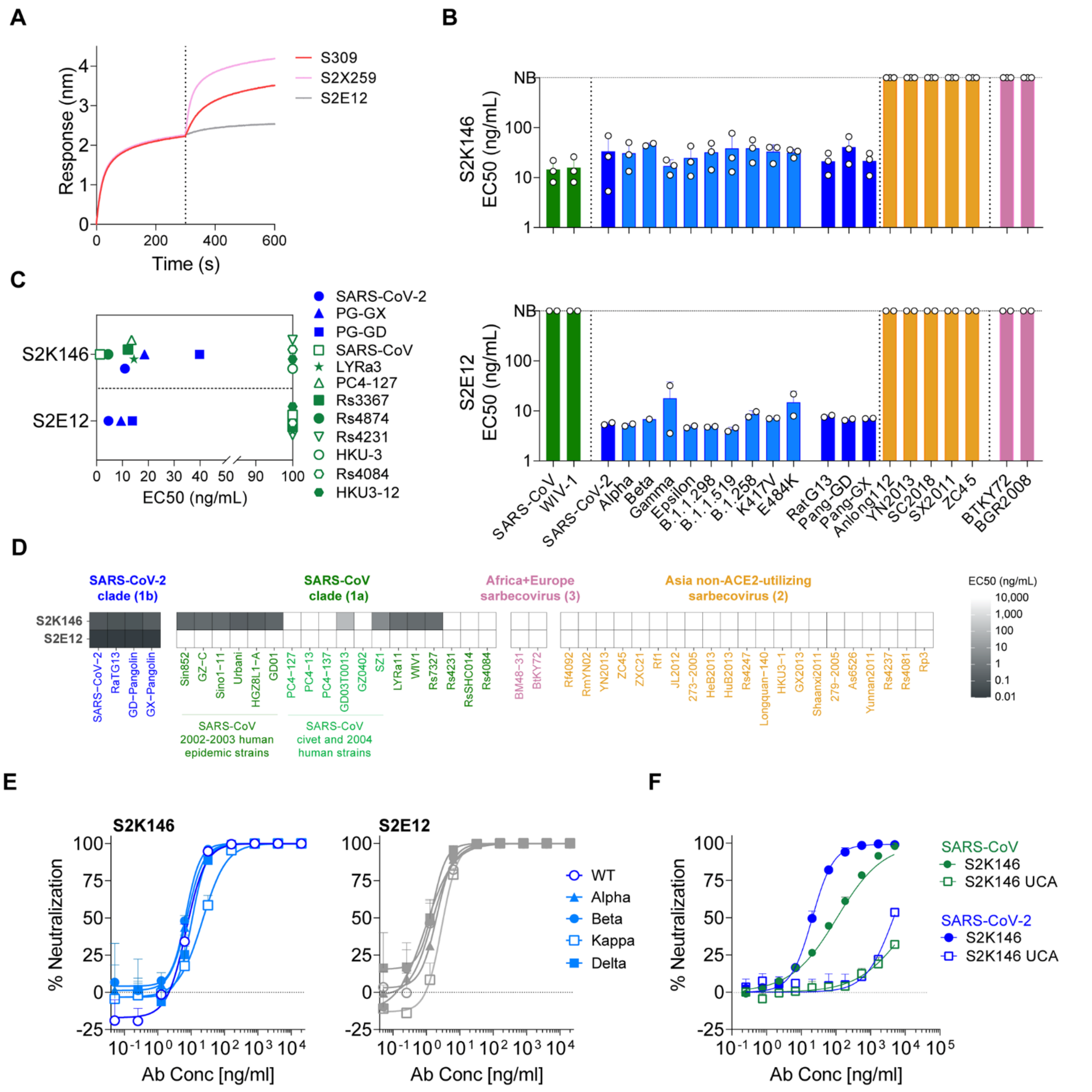
A
B
C
D
E
F
Response (nm)
Time (s)
S309
S2X259
S2E12
S2K146 EC50 (ng/mL)
S2E12 EC50 (ng/mL)
NB
SARS-CoV
WIV-1
SARS-CoV-2
Alpha
Beta
Gamma
Epsilon
B.1.1.298
B.1.1.519
B.1.258
K417V
E484K
Rat G13
Pang-GD
Pang-GX
Anlong112
YN2013
SC2018
SX2011
ZC45
BTKY72
BGR2008
S2K146
S2E12
EC50 (ng/mL)
SARS-CoV-2
PG-GX
PG-GD
SARS-CoV
LYRa3
PC4-127
Rs3367
Rs4874
Rs4231
HKU-3
Rs4084
HKU3-12
SARS-CoV-2 clade (1b)
SARS-CoV clade (1a)
Africa+Europe sarbecovirus (3)
Asia non-ACE2-utilizing sarbecovirus (2)
SARS-CoV 2002-2003 human epidemic strains
SARS-CoV civet and 2004 human strains
% Neutralization
Ab Conc [ng/ml]
WT
Alpha
Beta
Kappa
Delta
SARS-CoV
S2K146
S2K146 UCA
SARS-CoV-2
S2K146
S2K146 UCA

Fig. 1. Identification of the S2K146 cross-reactive and broadly neutralizing sarbecovirus mAb. (**A**) Binding of site I-targeting S2E12 (*33*), site IV-targeting S309 (*19*), or site II-targeting S2X259 (*19*) recombinant IgG1 (second phase) following association of S2K146 mAb (first phase) to His-tagged SARS-CoV-2 RBD immobilized on anti-His sensors, as measured by biolayer interferometry. (**B**) Cross-reactivity of S2K146 (upper panel) and S2E12 (lower panel) with 22 sarbecovirus RBDs from four sarbecovirus clades and SARS-CoV-2 variants analyzed by ELISA. EC50 of at least two independent experiments are shown. Error bars indicate standard deviation between experimental repeats. (**C**) Flow cytometry analysis of S2K146 cross-reactivity with a panel of 12 S glycoproteins representative of sarbecovirus clades 1a and 1b transiently expressed on the surface of mammalian cells. (**D**) S2K146 cross-reactivity with sarbecovirus RBDs displayed at the surface of yeast. (**E**) S2K146- and S2E12-mediated neutralization of replication-competent SARS-CoV-2 (USA-WA1/2020) and SARS-CoV-2 VOC viruses. (**F**) S2K146- and S2K146 UCA-mediated neutralization of VSV pseudotypes harboring SARS-CoV-2 S or SARS-CoV S. Error bars indicating standard deviation between replicates are represented only in one direction.

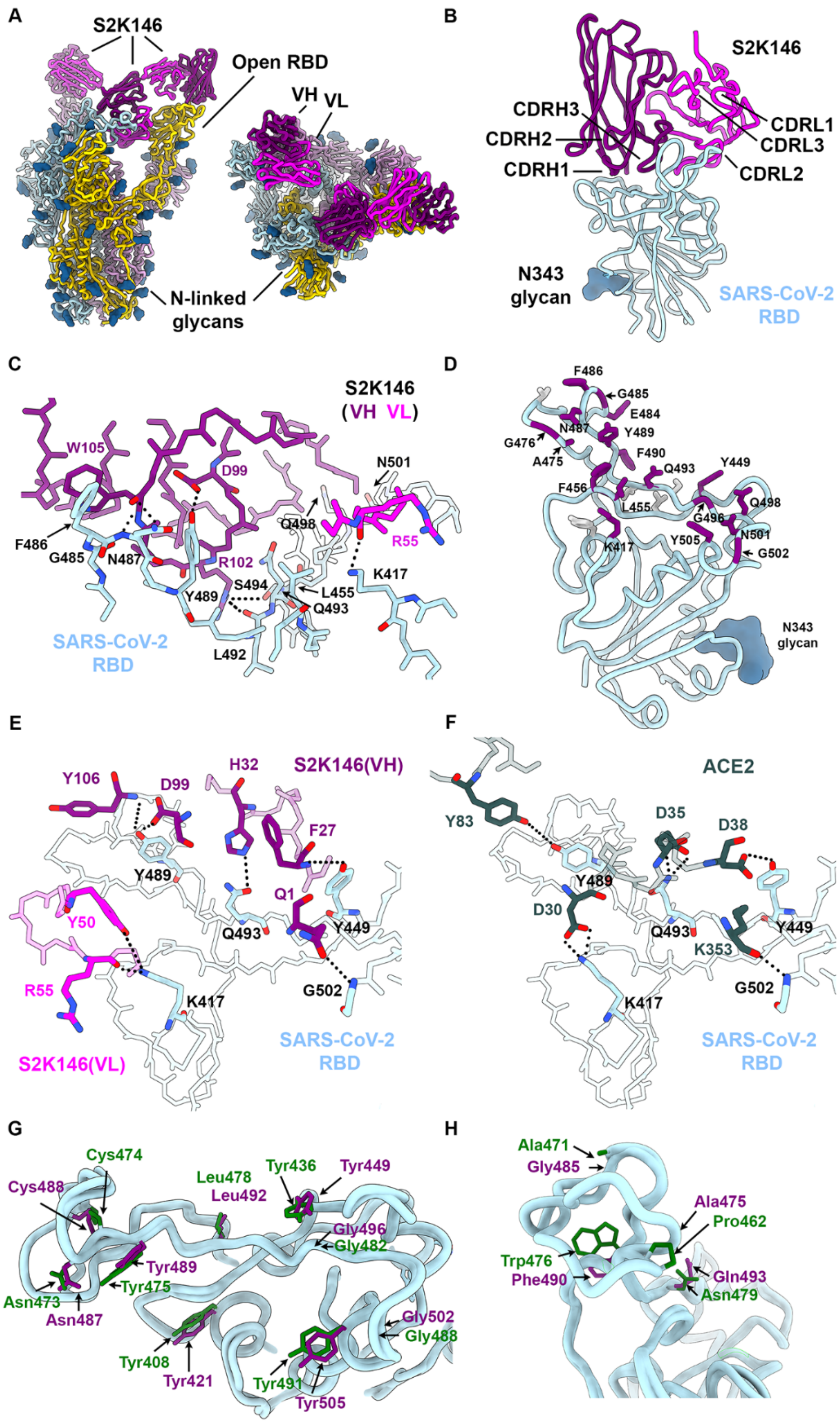
A
S2K146
Open RBD
VH
VL
N-linked
glycans
B
S2K146
CDRH3
CDRH2
CDRH1
CDRL1
CDRL3
CDRL2
N343
glycan
SARS-CoV-2
RBD
C
S2K146
(VH VL)
W105
D99
N501
Q498
R55
F486
G485
N487
R102
K417
S494
Y489
L455
Q493
L492
SARS-CoV-2
RBD
D
F486
G485
E484
G476
N487
Y489
A475
F490
Q493
Y449
F456
L455
G496
Q498
Y505
N501
K417
G502
N343
glycan
E
Y106
D99
H32
S2K146(VH)
F27
Y489
Y50
Q1
Q493
Y449
R55
K417
G502
S2K146(VL)
SARS-CoV-2
RBD
F
ACE2
Y83
D35
D38
Y489
D30
Q493
Y449
K353
G502
K417
SARS-CoV-2
RBD
G
Cys474
Cys488
Leu478
Leu492
Tyr436
Tyr449
Gly496
Gly482
Tyr489
Tyr475
Asn473
Asn487
Gly502
Gly488
Tyr408
Tyr421
Tyr491
Tyr505
H
Ala471
Gly485
Ala475
Pro462
Trp476
Phe490
Gln493
Asn479

Fig. 2. The S2K146 broadly neutralizing mAb recognizes RBD antigenic site I. (**A**) CryoEM structure viewed along two orthogonal orientations of the prefusion SARS-CoV-2 S ectodomain trimer with three S2K146 Fab fragments bound to two open RBDs and one partially closed RBD. SARS-CoV-2 S protomers are colored cyan, pink and gold. S2K146 heavy chain and light chain variable domains are colored purple and magenta, respectively. Glycans are rendered as blue spheres. (**B**) Ribbon diagram of the S2K146-bound SARS-CoV-2 RBD. (**C**) Zoomed-in view of the contacts formed between S2K146 and the SARS-CoV-2 RBD. Selected epitope residues are shown as sticks and electrostatic interactions are indicated with dotted lines. S2K146 heavy chain and light chain variable domains are colored as in panel A. (**D**) S2K146 epitope residues shown as sticks and colored purple (labeled) if they are involved in ACE2 binding or grey otherwise (unlabeled). (**E** and **F**) Similar electrostatic interactions (dotted lines) formed between S2K146 (E) or ACE2 (F) and the SARS-CoV-2 RBD. (**G**) The side chains of the nine S2K146 epitope residues conserved between the SARS-CoV-2 (purple) and SARS-CoV [green, PDB: 2AJF (*12*)] RBDs are shown as sticks. (**H**) The side chains of the four S2K146 epitope residues conservatively substituted between the SARS-CoV-2 (purple) and SARS-CoV (green) RBDs are shown as sticks.

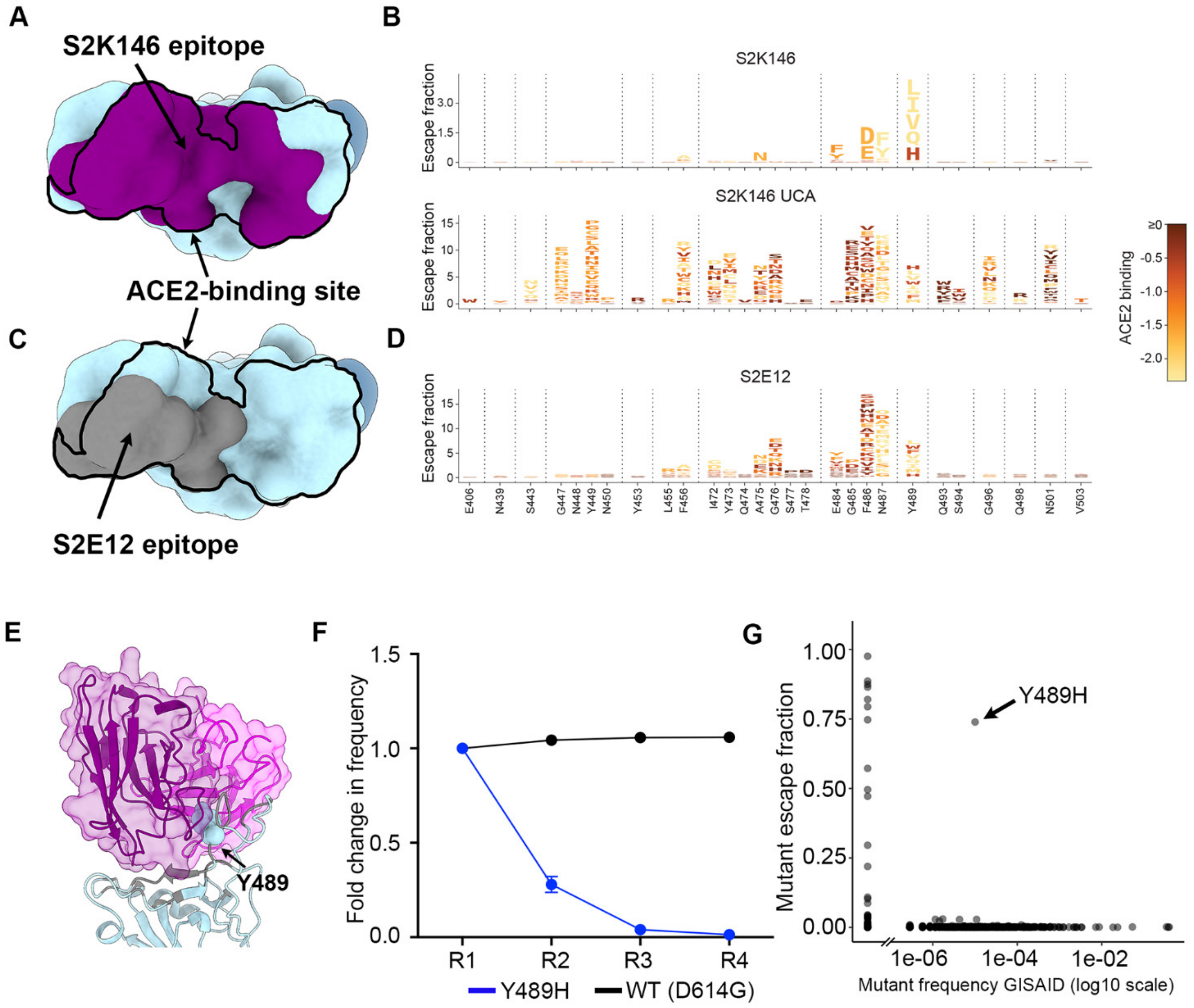

Fig. 3. S2K146 is resilient to a broad spectrum of escape mutations. (**A**) Molecular surface representation of the SARS-CoV-2 RBD with the S2K146 epitope colored purple and the ACE2 footprint indicated as a black outline. (**B**) Mapping of RBD mutations reducing S2K146 (top) or S2K146 UCA (bottom) binding using DMS of the yeast-displayed SARS-CoV-2 RBD. Sites of strong escape (pink underlines in fig. S7A) are shown in logo plot. Letters are colored according to how mutations affect the ACE2 binding affinity of the SARS-CoV-2 RBD, as measured via yeast display in Starr *et al*. (*30*). (**C**) Molecular surface representation of the SARS-CoV-2 RBD with the S2E12 epitope colored grey and the ACE2 footprint indicated as a black outline. The N343 glycan is rendered as blue spheres in A and C. (**D**) Mapping of RBD mutations reducing S2E12 binding using DMS of the yeast-displayed SARS-CoV-2 RBD. Sites of strong escape (purple underlines in fig. S7A) are shown in logo plot, as measured previously in Starr *et. al* (*20*). (**E**) Zoomed-in view of the S2K146-bound SARS-CoV-2 RBD (blue) highlighting the Y489H neutralization escape mutation. The S2K146 heavy and light chain variable domains are shown as ribbons within transparent purple and magenta surfaces, respectively. (**F**) Viral replication competition between VSV chimeras harboring the SARS-CoV-2 Wuhan-Hu-1/D614G S with or without the Y489H substitution using VeroE6 cells. (**G**) Mutations reducing binding of S2K146 to the RBD based on DMS (escape score) are plotted versus their frequencies among the human-derived SARS-CoV-2 sequences on GISAID as of Sep 27 2021. The large escape mutant (>5× global median escape fraction) with nonzero frequency is indicated.

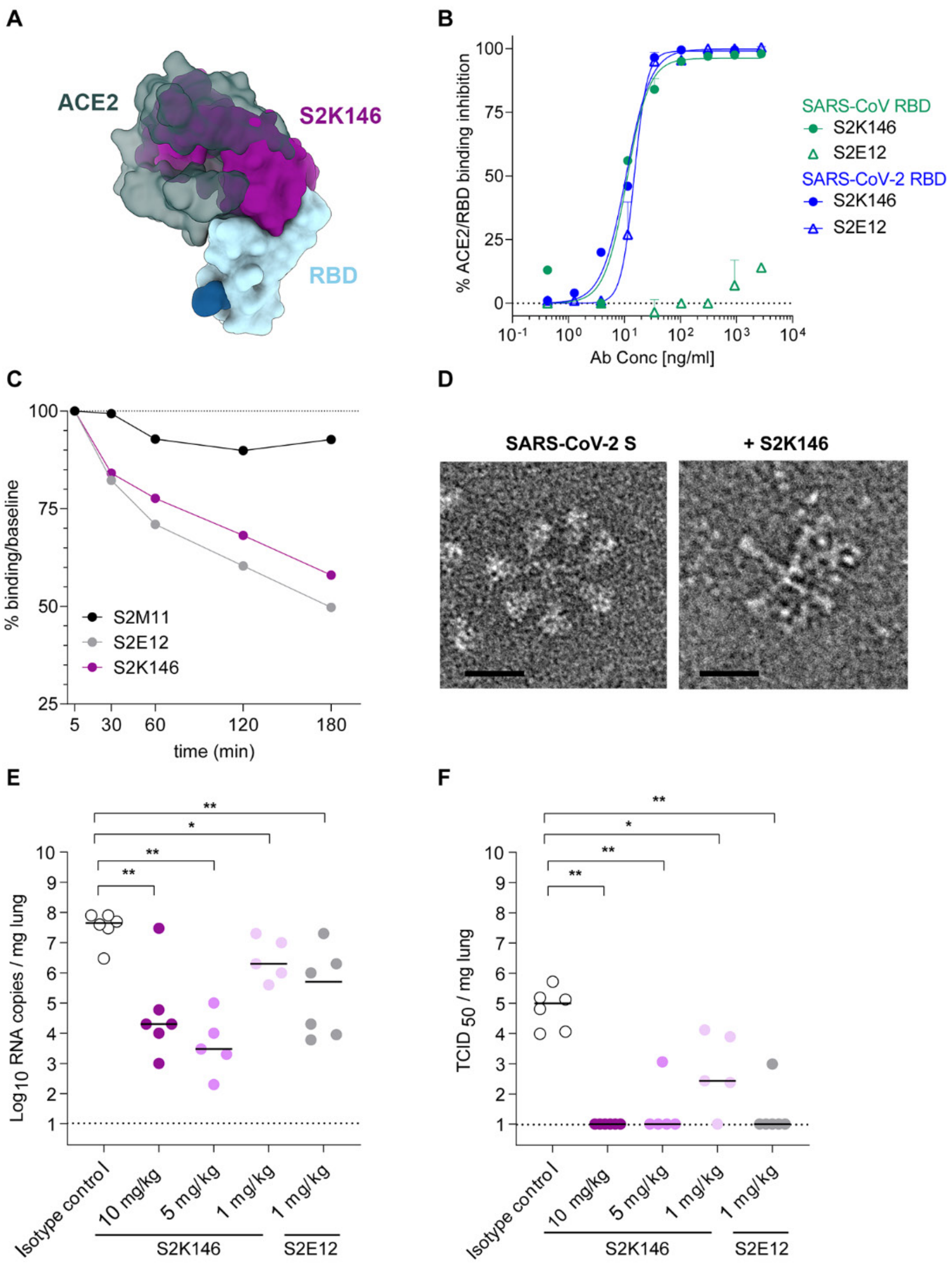

Fig. 4. S2K146 blocks receptor attachment, triggers premature S refolding and protects against SARS-CoV-2 challenge therapeutically. (**A**) Superimposition of the S2K146-bound (purple) and ACE2-bound [dark slate gray, PDB: 6M0J (*16*)] SARS-CoV-2 RBD (light blue) structures showing steric overlap. The N343 glycan is rendered as blue spheres. (**B**) Pre-incubation of serial dilutions of S2K146 with the SARS-CoV-2 RBD prevents binding to immobilized human ACE2 (hACE2) ectodomain in ELISA. Error bars indicate standard deviation between replicates. (**C**) S2K146-mediated S_1-shedding from cell-surface expressed SARS-CoV-2 S as determined by flow cytometry. S2E12 mAb was used as positive control whereas S2M11 was used as a negative control. (**D**) Cropped electron micrographs of negatively stained SARS-CoV-2 S trimer before (left, prefusion state) or after (right, postfusion state) incubation with S2K146. One representative micrograph for each dataset is shown out of 93 (SARS-CoV-2 S alone) and of 225 (SARS-CoV-2 S with S2K146) micrographs. The scale bar represents 200 Å. (**E** and **F**) Quantification of viral RNA (E) and replicating virus titers (TCID50), (F) in the lung of Syrian hamster 4 days post-intranasal infection with SARS-CoV-2 Beta VOC following therapeutic administration of S2K146 mAb at three different doses: 10-5-1mg/kg (n = 6/5 animal for each group). S2E12 mAb was administrated as control (n = 6 animals). Isotype control was administered at 10 mg/kg (n = 6 animals).
