# Supplementary material for: Antibody-mediated broad sarbecovirus neutralization through ACE2 molecular mimicry
Source: Science. 2022 Jan 6;375(6579):449–54. doi: 10.1126/science.abm8143 (PMC9400459; doi:10.1126/science.abm8143)
Supplement: Supplementary file 2 — Materials and Methods Figs. S1 to S10 Tables S1 and S2 References (54–76) [file science.abm8143_sm.pdf]

Science
AAAS

Supplementary Materials for

Antibody-mediated broad sarbecovirus neutralization through ACE2 molecular mimicry

Young-Jun Park *et al.*

Corresponding authors: Davide Corti, dcorti@vir.bio; Matteo Samuele Pizzuto, mpizzuto@vir.bio; David Veesler, dveesler@uw.edu

Science **375**, 449 (2022)
DOI: 10.1126/science.abm8143

The PDF file includes:

Materials and Methods
Figs. S1 to S10
Tables S1 and S2
References

Other Supplementary Material for this manuscript includes the following:

MDAR Reproducibility Checklist

Materials and Methods

Cell lines

Cell lines used in this study were obtained from ATCC (Vero-E6) or ThermoFisher Scientific (Expi CHO cells and Expi293F™ cells) or were generated via lentiviral transduction (Expi CHO-S, HEK293T-ACE2, Vero-TMPRSS2) (*37*).

Recombinant protein production

RBDs from different sarbecoviruses used in ELISA and BLI experiments were expressed with N-terminal signal peptide and C-terminal thrombin cleavage siteTwinStrep-8xHis-tag in Expi293F cells at 37°C and 8% CO_2. Cells were transfected using PEI MAX (Polysciences) at a DNA:PEI ratio of 1:3.75. Transfected cells were supplemented three days after transfection with 3 g/L glucose (Bioconcept) and 5 g/L soy hydrolysate (Sigma-Aldrich Chemie GmbH). Cell culture supernatant (423 mL) was collected seven days after transfection and supplemented with 47 mL 10x binding buffer (1 M Tris-HCl, 1.5 M NaCl, 20 mM EDTA, pH 8.0) and 25 mL BioLock (IBA GmbH) and incubated on ice for 30 min. Proteins were purified using a 5 mL Strep-Tactin XT Superflow high capacity cartridge (IBA GmbH) followed by buffer exchange to PBS using HiPrep 26/10 desalting columns (Cytiva).

SARS-CoV-2 S hexapro (*54*), used for cryo-EM single particle studies, was expressed and purified as described before (*33*).

The SARS-CoV-2 S 'wildtype' ectodomain trimer used for refolding experiments followed by negative stain EM was engineered as follows and recombinantly expressed as previously described (*37*). The SARS-CoV-2 S D614G 'wildtype' has a mu-phosphatase signal peptide ending in ETGT, begins at Q14, a mutated S1/S2 cleavage site (SGAR), ends at residue K1211 and is followed by a TEV cleavage site, fold-on trimerization motif, and an hexa-histidine tag in the pCMV vector.

The SARS-CoV-2 HexaPro construct was previously described (*54*) and placed into CMVR with an octa-histidine tag.

The SARS-CoV-2 Wuhan-Hu-1 RBD (*4*) was synthesized by GenScript into pcDNA3.1 and contains an N-terminal mu-phosphatase signal peptide, a C-terminal octa-histidine tag, flexible linker, and avi tag (GHHHHHHHHGGSSGLNDIFEAQKIEWHE). The boundaries of the construct are $_{328}$RFPN$_{331}$ and $_{528}$KKST$_{531}$.

The AncAsia-RBD sequence was taken from Starr et al. (*34*). The AncAsia-RBD-Avi construct was synthesized by GenScript into CMVR with an N-terminal mu-phosphatase signal peptide and a C-terminal octa-histidine tag, flexible linker, and avi tag (GHHHHHHHHGGSSGLNDIFEAQKIEWHE). The boundaries of the construct are $_{328}$RFPN$_{331}$ and $_{528}$KLST$_{531}$ (SARS-CoV-2 numbering).

SARS-CoV-2 S-D614G used for refolding experiments contains a mu-phosphatase signal peptide beginning at Q14, a mutated S_1/S_2 cleavage site (SGAR), ends at residue K1211 followed by a TEV cleavage site, foldon trimerization motif, and an octa-histidine tag in the pCMV vector.

SARS-CoV-2 S HexaPro, SARS-CoV-2 S D614G, SARS-CoV-2 Wuhan-Hu-1 RBD and AncAsia RBD were produced in Expi293F Cells (ThermoFisher Scientific)

grown in suspension using Expi293 Expression Medium (ThermoFisher Scientific) at 37°C in a humidified 8% CO_2 incubator rotating at 130 rpm. Cells grown to a density of 3 million cells per mL were transfected using the ExpiFectamine 293 Transfection Kit (ThermoFisher Scientific) and cultivated for 3-5 days. Proteins were purified from clarified supernatants using a nickel HisTrap HP affinity column (Cytiva) and washed with ten column volumes of 20 mM imidazole, 25 mM sodium phosphate pH 8.0, and 300 mM NaCl before elution on a gradient to 500 mM imidazole. Proteins were buffer exchanged into 20 mM sodium phosphate pH 8 and 100 mM NaCl and concentrated using centrifugal filters (Amicon Ultra) before being flash frozen.

Recombinant BtKY72 RBD production

As previously described, BtKY72 RBD construct (BtKY72 S residues 318-520) was synthesized by GenScript into a CMVR plasmid with a N-terminal mu-phosphatase signal peptide and a C-terminal hexa-histidine tag (-HHHHHHHH) joined by a short linker (-GGSS) to an Avi tag (-GLNDIFEAQKIEWHE) (*34*). BtKY72 mutant construct K493Y/T498W (BtKY72 S residue 482/487) were subcloned by GenScript from the BtKY72 RBD construct. BtKY72 and BtKY72 mutant RBD constructs were produced in Expi293F cells in Gibco Expi293 Expression Medium at 37°C in a humidified 8% CO_2 incubator rotating at 130 rpm. The cultures were transfected using PEI-25K with cells grown to a density of 3 million cells per mL and cultivated for 3-5 days. Proteins were purified from clarified supernatants using a 1mL HisTrap HP affinity column (Cytiva), concentrated, and then biotinylated with a commercial BirA kit (Avidity). Proteins were then purified from the BirA enzyme by affinity purification using a 1 mL HisTrap HP affinity column (Cytiva), concentrated, and flash frozen in 1x PBS, pH 7.4 as previously described (*34*).

Biolayer Interferometry (BLI) analysis of mAb binding to BtKY72 RBDs

Experiments were carried out on a Octet Red96 (Forte Bio) and all samples were prepared in 10x Kinetics Buffer (Forte Bio). To assess S2K146, S2X259, S2E12 association with wildtype BtKY72, BtKY72 K493Y/T498W, SARS-CoV-2 RBD, RBDs were immobilized on Ni-NTA biosensors (Forte Bio) that were hydrated in water for 10 min. The biosensors were equilibrated to the kinetics buffer for 60 seconds prior to loading of the RBDs. Loading occurred in 10-20 µg/mL RBD and normalized to a 1 nm shift, then a baseline signal was established in 10x Kinetics Buffer for 120 seconds. Association with S2K146, S2X259, and S2E12 was performed in 200 ug/mL of IgG, and dissociation was performed in 10x Kinetics buffer. Reference subtraction was performed with no load controls used to assess non-specific association between IgG solutions and the biosensor. Figures were plotted in Prism.

Antibody isolation and recombinant production

Antigen specific IgG^+ memory B cells were isolated and cloned from PBMC of SARS-CoV-2 convalescent individuals. Briefly, $CD19^+$ B cells were enriched by staining with CD19 PE-Cy7 and anti-PE microbeads (Milteniy), followed by positive selection using LS columns. Enriched B cells were stained with anti-IgD, anti-IgM, anti-IgA, anti-CD14, all PE labelled and prefusion SARS-CoV-2 S-Avi tag conjugated with streptavidin Alexa-Fluor 647 (Life Technologies). SARS-CoV-2-specific IgG^+ memory B cells were sorted

and seeded on MSC (mesenchymal stromal cells) at 0.5 cell/well in the presence of CpG2006, IL-2, IL6, IL-10 and IL-21, as previously described (*55*). After 7 days, B cell supernatants were screened by ELISA for binding to a panel of RBDs representative of different sarbecovirus clades as well as by neutralization using high-throughput VSV SARS-CoV-2 S-abesed microneutralization. Abs VH and VL sequences were obtained by reverse transcription PCR (RT-PCR) and mAbs were expressed as recombinant human IgG1, carrying the half-life extending M428L/N434S (LS) mutation in the Fc region fragment. ExpiCHO cells were transiently transfected with heavy and light chain expression vectors as previously described (*19*). Using the Database IMGT (http://www.imgt.org), the VH and VL gene family and the number of somatic mutations were determined by analyzing the homology of the VH and VL sequences to known human V, D and J genes. UCA sequences of heavy and light variable regions were constructed using IMGT/V-QUEST.

MAbs affinity purification was performed on ÄKTA Xpress FPLC (Cytiva) operated by UNICORN software version 5.11 (Build 407) using HiTrap Protein A columns (Cytiva) for full length human and hamster mAbs and CaptureSelect CH1-XL MiniChrom columns (ThermoFisher Scientific) for Fab fragments, using PBS as mobile phase. Buffer exchange to the appropriate formulation buffer was performed with a HiTrap Fast desalting column (Cytiva). The final products were sterilized by filtration through 0.22 µm filters and stored at 4ºC

Enzyme-linked immunosorbent assay

96 half area well-plates (Corning, 3690) were coated over-night at 4°C with 25 µl of sarbecoviruses RBD proteins WIV1 (AGZ48831.1), Anlong-112 (ARI44804.1), YN2013 (AIA62330.1), SC2018 (QDF43815.1), ZC45 (AVP78031.1), Rp/Shaanxi2011 (AGC74165.1), BM48-31/BGR/2008 (YP_003858584.1), RaTG13 (QHR63300.2), SARS-CoV2 (YP_009724390.1), SARS-CoV Urbani (AAP13441.1), BtKY72 (APO40579.1), Pangolin-Guangdong-2019 (EPI_ISL_410721), Pangolin_Guanxi-2017 (EPI_ISL_410539) and SARS-CoV-2 RBD mutants, prepared at 5 µg/ml in PBS pH 7.2. After a blocking step of 60 min at room temperature with PBS 1% BSA (Sigma-Aldrich, A3059), plates were incubated with mAb serial dilutions for 60 min at room temperature. After 4 washing steps with PBS 0.05% Tween 20 (PBS-T) (Sigma-Aldrich, 93773), goat anti-human IgG secondary antibody (Southern Biotech, 2040-04) was added and incubated for 45 min at room temperature. Plates were then washed 4 times with PBS-T and 4-NitroPhenyl phosphate (pNPP, Sigma-Aldrich, 71768) substrate was added. After 45 min incubation, absorbance at 405 nm was measured by a plate reader (Biotek) and data plotted using Prism GraphPad.

30 µL of 5 µg/mL of SARS-CoV-2 AncAsia RBD diluted in PBS was incubated on a 384-well Nunc Maxisorp plate (ThermoFisher 464718) overnight at 4˚C. Plates were slapped dry before addition of 60 µL blocker Casein in PBS (ThermoFisher) and incubation for one hour at 37˚C. Plates were slapped dry and a 1:4 serial dilution of mAbs beginning at 300 µg/mL was added and incubated for one hour at 37˚C. Plates were slapped dry and washed 4x with TBST using a BioTek plate washer followed by

addition of Invitrogen anti-Human IgG (ThermoFisher A18817) and one hour incubation at 37˚C. Plates were once again slapped dry and washed 4x with TBST before addition of room temperature TMB Microwell Peroxidase (Seracare 5120-0083). The reaction was quenched after 1-2 minutes with 1 N HCl and the A450 of each well was read using a BioTek plate reader. Prism (GraphPad) nonlinear regression with "Sigmoidal, 4PL, X is concentration" was used to determine the ED_{50} of each sample.

Transient Expression of sarbecovirus S protein in ExpiCHO-S Cells
ExpiCHO cells were seeded at 6 × 10^6 cells/ml into 50 ml bioreactor tubes in 5 ml culture medium. Spike coding plasmids (5 μg) were diluted in OptiPRO SFM, mixed with ExpiFectamine CHO Reagent (Life Technologies) and added to the cells. After transfection, cells were incubated at 37°C with 8% CO_2 with an orbital shaking speed of 120 rpm (orbital diameter of 25 mm) for 48 hours.

Binding to cell surface expressed sarbecovirus S proteins by flow cytometry
Transiently transfected ExpiCHO cells were harvested and washed in wash buffer (PBS 2% FBS, 2 mM EDTA). Cells were counted, distributed into round bottom 96-well plates (Corning) and incubated with serially diluted antibodies in wash buffer (starting concentration: 10 μg/ml, 8 points of dilution 1:4). Alexa Fluor647-labeled Goat Anti-Human IgG secondary Ab (Jackson Immunoresearch) was prepared at 2 µg/mL added onto cells after two washing steps. Cells were then washed twice and resuspended in wash buffer for data acquisition at ZE5 cytometer (Biorad).

Competition assay and affinity determination by Biolayer Interferometry (BLI)
BLI experiments were carried out using an Octet Red96 (ForteBio) and all reagents were prepared in Kinetics buffer (KB) (PBS 0.01% BSA).
To assess S2K146 competition with S2X259, S309 and S2E12, His-tagged SARS-CoV-2 RBD was prepared at 8 µg/ml in Kinetics buffer (PBS 0.01% BSA) and loaded on pre-hydrated anti-penta-HIS biosensors (Sartorius) for 2.5 min. Biosensors were then moved into a solution containing 20 µg/ml S2K146 mAb and association recorded for 5 min. A second association step was subsequently performed into S2X259, S309 and S2E12 mAbs solutions at 20 µg/ml and recorded for 5 min. Response values were exported and plotted using GraphPad Prism (version 9.1.1).

To assess binding affinities, S2K146 and respective UCA Ab were prepared at 3 μg/ml and immobilized on pre-hydrated protein-A biosensors (Sartorius) for 75 sec. After a 30 sec stabilization step in KB, biosensors were moved in SARS-CoV or SARS-CoV-2 :2 dilution series (starting concentration: 18.5 nM) for the 600 sec association step, and then moved back in KB to record dissociation signals for 540 sec. The data were baseline subtracted, results fitted using the Pall FortéBio/Sartorius analysis software (version 12.0) and plotted using GraphPad Prism (version 9.1.1)

VSV-based pseudovirus production and neutralization assay
VSV psudoviruses were produced using the following constructs: SARS-CoV S, SARS-CoV-2 S, WIV-1 S, RaTG13 S, PG-GD S, PG-GX S, the VOC Alfa, Beta, Gamma,

Epsilon, the VOI B.1.1.519, B.1.1.218 and SARS-CoV2 S bearing the single mutations K417V, E484K. Pseudotyped viruses were prepared using Lenti-X 293 cells seeded in 15-cm dishes. Briefly, cells in culture medium (DMEM supplemented with 10% heat-inactivated FBS, 1% PenStrep) were transfected with 25 μg of plasmid encoding for the corresponding S glycoprotein using TransIT-Lenti (Mirus) as transfectant reagent. One day post-transfection, cells were infected with VSV (G*ΔG-luciferase) for 1 h, washed 3 times in PBS with Ca^{2+}/Mg^{2+} (Thermo Fisher) before adding 25 ml of culture medium/dish. Particles were harvested after 18-24 h, clarified from cellular debris by centrifugation at 2,000 x g for 20 min at 4°C, aliquoted and stored at −80°C until use in neutralization experiments.

For neutralization experiments, Vero E6 cells were seeded at 20,000 cells/well in culture medium into white 96-well plates (PerkinElmer, 6005688) and cultured overnight at 37°C 5% CO_2. Ten-point 3-fold mAb serial dilutions were prepared in culture medium and mixed 1:1 with pseudotyped VSV prepared in culture medium in order to infect cells with the desired MOI. After 60 min incubation at 37 °C, cell culture medium was aspirated and 50 μl of PVs/mAb mixture was added onto cells and incubated 60 min at 37°C 5% CO_2. After 60 min, 100 μl of culture medium was added to the cells and incubation at 37°C 5% CO_2 followed in the next 16-24 h. At the end of the incubation time, culture medium was removed from the cells and 50 μl/well of Steadylite (PerkinElmer) diluted 1:2 with PBS with $Ca^{2+}Mg^{2+}$ was added to the cells and incubated in the dark for 10 min. Luminescence signals were read using a Synergy H1 Hybrid Multi-Mode plate reader (Biotek). Measurements were done in duplicate and at least six wells per plate contained untreated infected cells (defining the 0% of neutralization, "MAX RLU" value) and infected cells in the presence of S2E12 and S2X259 at 25 μg/ml each (defining the 100% of neutralization, "MIN RLU" value). Average of Relative light units (RLUs) of untreated infected wells (MAX RLUave) was subtracted by the average of MIN RLU (MIN RLUave) and used to normalize percentage of neutralization of individual RLU values of experimental data according to the following formula: (1-(RLUx - MIN RLUave) / (MAX RLUave – MIN RLUave)) x 100. Data were analyzed and visualized with Prism (Version 9.1.1). IC50 values were calculated from the interpolated value from the log(inhibitor) versus response, using variable slope (four parameters) nonlinear regression with an upper constraint of ≤100, and a lower constrain equal to 0.

BtKY72 (K493Y/T498W) S pseudovirus production and neutralization assay
VSV pseudovirus harboring BtKY72 K493Y/T498W S (based on SARS-CoV-2 numbering) with a native signal peptide and C-terminal 21 residue deletion synthesized by GenScript were prepared as previously described (*34*). Briefly, HEK-293T cells seeded in poly-D-lysine coated 100 mm dishes at ~75 % confluency were washed five times with Opti-MEM and co-transfected with Lipofectamine 2000 (Life Technologies) with 24 μg of the S glycoprotein plasmids. After 5 h at 37°C, media supplemented with 20% FBS and 2% PenStrep was added. After 20 hours, cells were washed five times with DMEM and cells were transduced with VSVΔG-luc (*56*) and incubated at 37°C. After 2 h, infected cells were washed an additional five times with DMEM prior to adding media supplemented with anti-VSV-G antibody (I1-mouse hybridoma supernatant diluted 1:25, from CRL-2700, ATCC) to reduce parental background. After 18-24 h, the supernatant

was harvested and clarified by low-speed centrifugation at 2,500 g for 10 min. The supernatant was then filtered (0.45 µm) and concentrated 10 times using a 30 kDa cut off membrane. The pseudotypes were then aliquoted and frozen at -80 °C.
For neutralization experiments, HEK-293T cells expressing hACE2 (Crawford et al. 2020) in DMEM supplemented with 10% FBS and 1% PenStrep were seeded at 20,000 cells per well into clear bottom, white manually poly-D-lysine coated 96 well plates and incubated at 37°C. The following day, an additional half-area, 96-well plate was prepared with twelve 3-fold serial dilutions of mAb of either S2K146 or S2X259 starting at 250ug/mL or 25 ug/mL respectively. An equal volume of diluted pseudovirus was added and incubated for 30 minutes at room temperature. Excess media was removed from cells and the mAb-pseudovirus mixture was transferred to the cells for 2 hours at 37C. After the 2 hour incubation, an equal volume of DMEM-20%FBS-2%PenStrep was added for overnight incubation. The following day, One-Glo-EX substrate (Promega) was added and incubated in the dark for 5 minutes. The plates were immediately read on a Biotek plate reader. Relative luciferase units were plotted and normalized in Prism with cells alone without pseudovirus defining 100% neutralization and cells with pseudovirus only defining 0% neutralization. Data were analyzed and visualized with Prism (Version 9.1.1). IC50 values were calculated from the interpolated value from the log(inhibitor) versus response, using variable slope (four parameters) nonlinear regression with an upper constraint of ≤100, and a lower constrain equal to 0.

Authentic SARS-CoV-2 isolates

SARS-CoV-2 strains used in this study were obtained from BEI (WT: Lineage A, BEI ref. NR-5228; Alpha: Lineage B.1.1.7, BEI ref. NR-54000; Beta: Lineage B.1.351, BEI ref. NR-54009; Kappa: Lineage B.1.617.1, BEI ref. NR-55486; Delta: Lineage B.1.617.2, BEI ref. NR-55611).

Neutralization of authentic SARS-CoV-2 viruses

Vero-TMPRSS2 cells were seeded into black-walled, clear-bottom 96-well plates at 2 × 10^4 cells/well and cultured overnight at 37°C. The next day, 9-point 5-fold serial dilutions of mAbs were prepared in infection media (DMEM + 10% FBS). The different SARS-CoV-2 strains were diluted in infection media at a final MOI of 0.01 PFU/cell, added to the mAb dilutions and incubated for 30 min at 37°C. Media was removed from the cells, mAb-virus complexes were added and incubated at 37°C for 18 h. Cells were fixed with 4% PFA (Electron Microscopy Sciences, #15714S), permeabilized with Triton X-100 (SIGMA, #X100-500ML) and stained with an antibody against the viral nucleocapsid protein (Sino Biologicals, #40143-R001) followed by a staining with the nuclear dye Hoechst 33342 (Fisher Scientific, # H1399) and a goat anti-rabbit Alexa Fluor 647 antibody (Invitrogen, #A-21245). Cells were imaged with an automated multimode plate reader (Biotek, Cytation 5).

Blockade of SARS-CoV and SARS-CoV-2 binding to ACE2

SARS-CoV and SARS-CoV-2 mouse/rabbit Fc-tagged RBDs (final concentration 20 ng/ml) were incubated with serially diluted recombinant mAbs (from 25 µg/ml) and incubated for 1 h 37°C. The complex RBD:mAbs was then added to a pre-coated hACE2

(2 μg/ml in PBS) 96-well plate MaxiSorp (Nunc) and incubated 1 hour at room temperature. Subsequently, the plates were washed and a goat anti-mouse/rabbit IgG (Southern Biotech) coupled to alkaline phosphatase (Jackson Immunoresearch) added to detect mouse Fc-tagged RBDs binding. After further washing, the substrate (p-NPP, Sigma) was added, and plates read at 405 nm using a microplate reader (Biotek). The percentage of inhibition was calculated as follow: (1-((OD sample-OD neg ctr)/(OD pos. ctr-OD neg. ctr))*100

Cell-surface mAb-mediated S_1 shedding
CHO cells stably expressing the prototypic SARS-CoV-2 Spike protein were harvested, washed in wash buffer (PBS 1% BSA 2 mM EDTA) and resuspended in PBS. Cells were then counted and 90'000 cells/well were dispensed into a round-bottom 96 well plate (Corning) to be treated with 10 ug/ml TPCK-Trypsin (Worthington Biochem) for 30 min at 37°C. After a washing step, cells were incubated with 15 ug/ml mAbs solution for 180, 120, 60, 30 or 5 min at 37°C. After the incubation for the allotted time, cells were washed with ice-cold wash buffer and stained with 1.5 ug/ml Alexa Fluor647-labeled Goat Anti-Human IgG secondary Ab (Jackson Immunoresearch) for 30 min on ice in the dark. Cells were then washed twice with cold wash buffer and analyzed using a ZE5 cytometer (Biorad) with acquisition chamber T= 4°C. Binding at each time point (MFI) was determined normalizing to the MFI at 5 minutes time point and data plotted using GraphPad Prism v. 9.1.1

Evaluation of escape mutants via deep mutational scanning
A previously described deep mutational scanning approach (*57*) was used to identify RBD mutations that escape S2K146 binding exactly as described in (*20*). Briefly, duplicate libraries containing virtually all possible amino acid changes compatible with ACE2 binding and RBD folding within the Wuhan-Hu-1 SARS-CoV-2 RBD sequence were expressed on the surface of yeast. Libraries were labelled at 63 ng/mL S2K146 or 83 ng/mL S2K146 UCA antibody (the EC90 for binding to yeast-displayed SARS-CoV-2 RBD determined in isogenic pilot binding experiments), and fluorescence-activated cell sorting (FACS) was used to select RBD+ cells that exhibit reduced antibody binding as previously described. Libraries were sequenced before and after selection to determine per-mutation escape fractions as previously described. Experiments were performed in duplicate with independently generated mutant libraries, and we report the average mutant escape fraction across the duplicates. Data for S2E12 exactly as described in (*20*) are included for comparison to S2K146. Complete computational pipeline for deep mutational scanning data analysis is available on GitHub: https://github.com/jbloomlab/SARS-CoV-2-RBD_MAP_S2K146.

Evaluation of sarbecovirus cross-reactivity via high-throughput yeast-display binding assays
Evaluation of mAb cross-reactivity with a panel of yeast-displayed sarbecovirus RBDs was performed as described with modifications (*20*). Whereas we previously assessed mAb binding to the sarbecovirus RBD panel at a single concentration (analogous to DMS selections), binding was here determined via FACS-seq for a mAb dilution series (10,000 to 0.01 ng/mL in 10-fold dilutions, plus 0 ng/mL mAb). The 'escape

fraction' of each sarbecovirus RBD at each mAb concentration was fit to a sigmoid binding curve to determine the quantitative EC50 for antibody binding to each RBD. In addition to S2K146 breadth, S2E12 was profiled using this new method in this paper to enable direct comparison. Complete computational pipeline for the analysis of breadth of mAb binding is available on GitHub: https://github.com/jbloomlab/SARSr-CoV_RBD_MAP.

Selection of SARS-CoV-2 monoclonal antibody escape mutants

A VSV-SARS-CoV-2 Wuhan-Hu-1 D614G S chimera was used to select for mAb resistant mutants, as previously described(*35*). Briefly, mutants were recovered by plaque isolation on Vero E6 cells with the indicated mAb in the overlay. The concentration of mAb in the overlay was determined by neutralization assays at a multiplicity of infection (MOI) of 100. Escape clones were plaque-purified on Vero E6 cells in the presence of mAb, and plaques in agarose plugs were amplified on MA104 cells with the mAb present in the medium. Viral stocks were amplified on MA104 cells at an MOI of 0.01 in Medium 199 containing 2% FBS and 20 mM HEPES pH 7.7 (Millipore Sigma) at 34°C. Viral supernatants were harvested upon extensive cytopathic effect and clarified of cell debris by centrifugation at 1,000 x g for 5 min. Aliquots were maintained at - 80°C. Viral RNA was extracted from VSV-SARS-CoV-2 S mutant viruses using RNeasy Mini kit (Qiagen), and the S gene was amplified using OneStep RT-PCR Kit (Qiagen). The mutations were identified by Sanger sequencing (GENEWIZ). Their resistance was verified by subsequent virus infection in the presence or absence of mAb. Vero E6 cells were seeded into 12 well plates overnight. The virus was serially diluted using DMEM and cells were infected at 37°C for 1 h. Cells were cultured with an agarose overlay in the presence or absence of mAb at 34°C for 2 days. Plates were scanned on a biomolecular imager and expression of eGFP monitored at 48 hours post-infection.

Determination of binding affinity to Wuhan-hu-1 and Yusing surface plasmon resonance

Measurements were performed using a Biacore T200 instrument. A CM5 chip with covalently immobilized StrepTactin XT was used for surface capture of StrepTag-containing RBDs. Running buffer was HBS-EP+ pH 7.4 (Cytiva) and measurements were performed at 25℃. Experiments were performed with a 3-fold dilution series of monomeric hACE2: 300, 100, 33, 11 nM and were run as single-cycle kinetics. Data were double reference-subtracted and fit to a binding model using Biacore Evaluation software. The 1:1 binding model was used except for S2K146 Fab binding to Y489H which was fit to a Heterogeneous Ligand model (a low-amplitude kinetic phase with very slow dissociation was assumed to be an artifact; the higher amplitude, faster-dissociation kinetic phase was interpreted as the affinity of the Fab:RBD interaction; the resulting K_D is reported as approximate).

Viral replication fitness assays

Vero E6 cells (ATCC, CRL-1586) were seeded at 1×10^6 cells per well in 6-well plates. Cells were infected with multiplicity of infection (MOI) of 0.02, with VSV chimeras harboring SARS-CoV-2 Wuhan-Hu-1 D614G S and SARS-CoV-2 Wuhan-Hu-1

Y489H/D614G S mixed at equal titers. Following 1 h incubation, cell monolayers were washed three times with Hanks' Balanced Salt Solution (HBBS) and cultures were incubated for 72 h in humidified incubators at 34°C. To passage the progeny viruses, virus mixture was continuously passaged four times in Vero E6 cells at MOI of 0.02. Cellular RNA samples from each passage were extracted using RNeasy Mini kit (QIAGEN) and subjected to next-generation sequencing as described previously to confirm the introduction and frequency of substitutions (*35*).

S2K146-induced S refolding

10 μM native-like SARS-CoV-2 S(*37*) was incubated with 13 μM S2K146 Fab for 12 hours at room temperature. Samples were diluted to 0.01 mg/mL immediately prior to adsorption to glow-discharged carbon-coated copper grids for ~30 sec prior to a 2% uranyl formate staining. Micrographs were recorded using the Leginon software on a 120 kV FEI Tecnai G2 Spirit with a Gatan Ultrascan 4000 4k x 4k CCD camera at 67,000 nominal magnification. The defocus ranged from -1.0 to -2.0 μm and the pixel size was 1.6 Å.

CryoEM sample preparation, data collection and data processing

Recombinantly expressed and purified S2K146 Fab and SARS-CoV-2 S hexapro(*54*) were incubated at 1 mg/ml (for UltraAuFoil grids) or 0.1mg/ml (for lacey thin carbon grids) with a 1.2 molar excess of Fab at 4°C for 1 hr. Three microliters of the complex mixture were loaded onto freshly glow discharged R 2/2 UltrAuFoil grids (200 mesh) or lacey grids covered with a thin layer of manually added carbon, prior to plunge freezing using a vitrobot MarkIV (ThermoFisher Scientific) with a blot force of 0 and 6-6.5 sec blot time (for the UltrAuFoil grids) or with a blot force of −1 and 2.5 sec blot time (for the lacey thin carbon grids) at 100 % humidity and 22°C.

Data were acquired using an FEI Titan Krios transmission electron microscope operated at 300 kV and equipped with a Gatan K3 direct detector and Gatan Quantum GIF energy filter, operated in zero-loss mode with a slit width of 20 eV. Automated data collection was carried out using Leginon (*58*) at a nominal magnification of 105,000x with a pixel size of 0.843 Å. The dose rate was adjusted to 15 counts/pixel/s, and each movie was acquired in super-resolution mode fractionated in 75 frames of 40 ms. 7,289 micrographs were collected with a defocus range comprised between -0.5 and -2.5 μm. Movie frame alignment, estimation of the microscope contrast-transfer function parameters, particle picking, and extraction were carried out using Warp (*59*).

Two rounds of reference-free 2D classification were performed using CryoSPARC (*60*) to select well-defined particle images. These selected particles were subjected to two rounds of 3D classification with 50 iterations each (angular sampling 7.5˚ for 25 iterations and 1.8˚ with local search for 25 iterations), using our previously reported closed SARS-CoV-2 S structure as initial model (PDB 6VXX) (*4*) using Relion (*61*). 3D refinements were carried out using non-uniform refinement along with per-particle defocus refinement in CryoSPARC (*62*). Selected particle images were subjected to the Bayesian polishing procedure (*63*) implemented in Relion3.0 before performing another round of non-uniform refinement in cryoSPARC followed by per-particle defocus refinement and again non-uniform refinement. To improve the density of the S/S2K146 interface, the particles from the class with 2 RBDs opened were subjected to focus 3D

classification without refining angles and shifts using a soft mask on the closed RBD and bound S2K146 variable domains with a tau value of 60 in Relion. Particles belonging to classes with the best resolved local density were selected and subject to local refinement using CryoSPARC. Local resolution estimation, filtering, and sharpening were carried out using CryoSPARC. Reported resolutions are based on the gold-standard Fourier shell correlation (FSC) of 0.143 criterion and Fourier shell correlation curves were corrected for the effects of soft masking by high-resolution noise substitution (*64*, *65*).

Model building and refinement

UCSF Chimera (*66*) and Coot (*67*) were used to fit atomic models into the cryoEM maps. Spike-RBD/S2K146 model was refined and relaxed using Rosetta using sharpened and unsharpened maps (*68*, *69*). Model validation and analysis used MolProbity (*70*), EMringer (*71*), Phenix (*72*) and Privateer (*73*). Figures were generated using UCSF ChimeraX (*74*).

Measurement of Fc-effector functions

S2K146-dependent activation of human FcγRIIa and IIIa was performed with a bioluminescent reporter assay. ExpiCHO cells stably expressing full-length wild-type SARS-CoV-2 S (target cells) or full-length prefusion stabilized SARS-CoV-2 S, which harbours the 2P mutation and S1/S2 furin cleavage site mutation (RRARS to SGAG) as previously described (*4*), were incubated with different amounts of mAbs. After a 15-minute incubation, Jurkat cells stably expressing FcγRIIIa receptor (V158 variant) or FcγRIIa receptor (H131 variant) and NFAT-driven luciferase gene (effector cells) were added at an effector to target ratio of 6:1 for FcγRIIIa and 5:1 for FcγRIIa. Signaling was quantified by the luciferase signal produced as a result of NFAT pathway activation. Luminescence was measured after 20 hours of incubation at 37˚C with 5% CO_2 with a luminometer using the Bio-Glo-TM Luciferase Assay Reagent according to the manufacturer’s instructions (Promega).

Hamster challenge experiment.

The hamster infection model of SARS-CoV-2 including the associated analytical procedures, have been described before (*39*, *40*). In brief, female Syrian hamsters (Mesocricetus auratus) of 6-8 weeks old were anesthetized with ketamine/xylazine/atropine and inoculated intranasally with 50 µL containing 1×10^4 TCID50 Beta B.1.351 (derived from hCoV-19/Belgium/rega-1920/2021; EPI_ISL_896474, 2021-01-11). This variant was originally isolated in house from nasopharyngeal swabs taken from travelers returning to Belgium (baseline surveillance) and were subjected to sequencing on a MinION platform (Oxford Nanopore) directly from the nasopharyngeal swabs (*39*). Animals were treated once by intraperitoneal injection 24h post SARS-CoV-2 challenge (i.e. therapeutic administration) with S2K146 mAb (at 10, 5 and 1 mg/Kg). S2E12 mAb was administrated as control (at 1 mg/kg). Isotype control was administered at 10 mg/kg. Hamsters were monitored for appearance, behavior and weight. At day 4 pi, hamsters were euthanized by i.p. injection of 500 µL Dolethal (200mg/ml sodium pentobarbital, Vétoquinol SA). Lungs were collected for viral RNA and

infectious virus quantification by RT-qPCR and end-point virus titration, respectively. Serum samples were collected at day 4 pi for analysis of Ab levels.

SARS-CoV-2 RT-qPCR

Hamster lung tissues were collected after sacrifice and were homogenized using bead disruption (Precellys) in 350 µL TRK lysis buffer (E.Z.N.A.® Total RNA Kit, Omega Bio-tek) and centrifuged (10.000 rpm, 5 min) to pellet the cell debris. RNA was extracted according to the manufacturer's instructions. RT-qPCR was performed on a LightCycler96 platform (Roche) using the iTaq Universal Probes One-Step RT-qPCR kit (BioRad) with N2 primers and probes targeting the nucleocapsid (*39*). Standards of SARS-CoV-2 cDNA (IDT) were used to express viral genome copies per mg tissue.

End-point virus titrations

Lung tissues were homogenized using bead disruption (Precellys) in 350 µL minimal essential medium and centrifuged (10,000 rpm, 5min, 4°C) to pellet the cell debris. To quantify infectious SARS-CoV-2 particles, endpoint titrations were performed on confluent Vero E6 cells in 96- well plates. Viral titers were calculated by the Reed and Muench method (*75*) using the Lindenbach calculator and were expressed as 50% tissue culture infectious dose ($TCID_{50}$) per mg tissue.

Supplemental figures

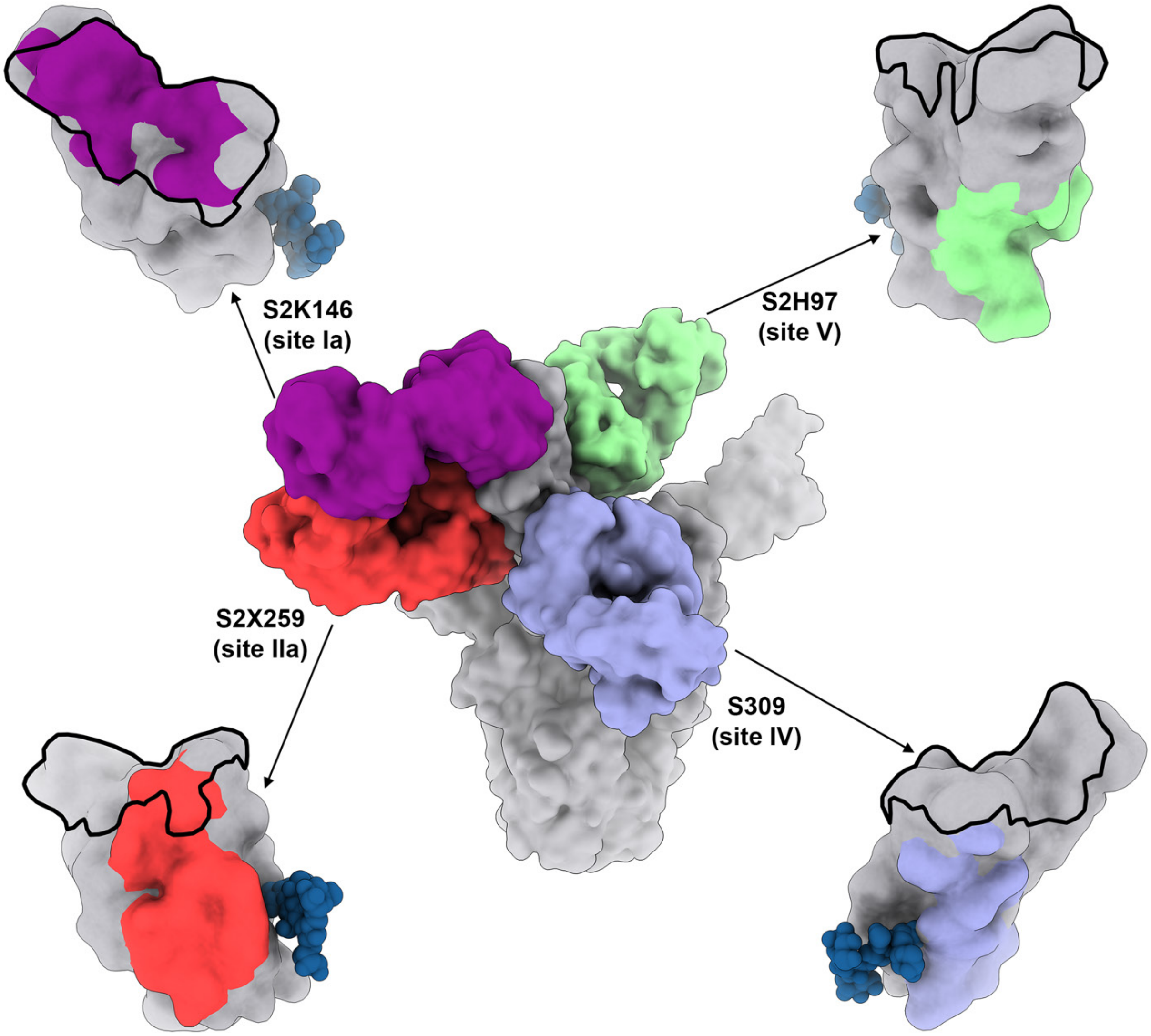

Figure S1. Overview of SARS-CoV-2 RBD cross-neutralizing antigenic sites. Composite model of the SARS-CoV-2 S-protein trimer (gray) with four distinct mAbs (S2K146 in purple, S2X259 in red (*19*), S2H97 in green (*20*), and S309 in blue (*21*)) bound to one RBD in the open conformation. The magnified panels show the footprint of each mAb on the SARS-CoV-2 RBD with matching color and the ACE2-binding site is outlined in black. The glycan at position N343, which is conserved across the sarbecovirus subgenus, is rendered as dark blue spheres.

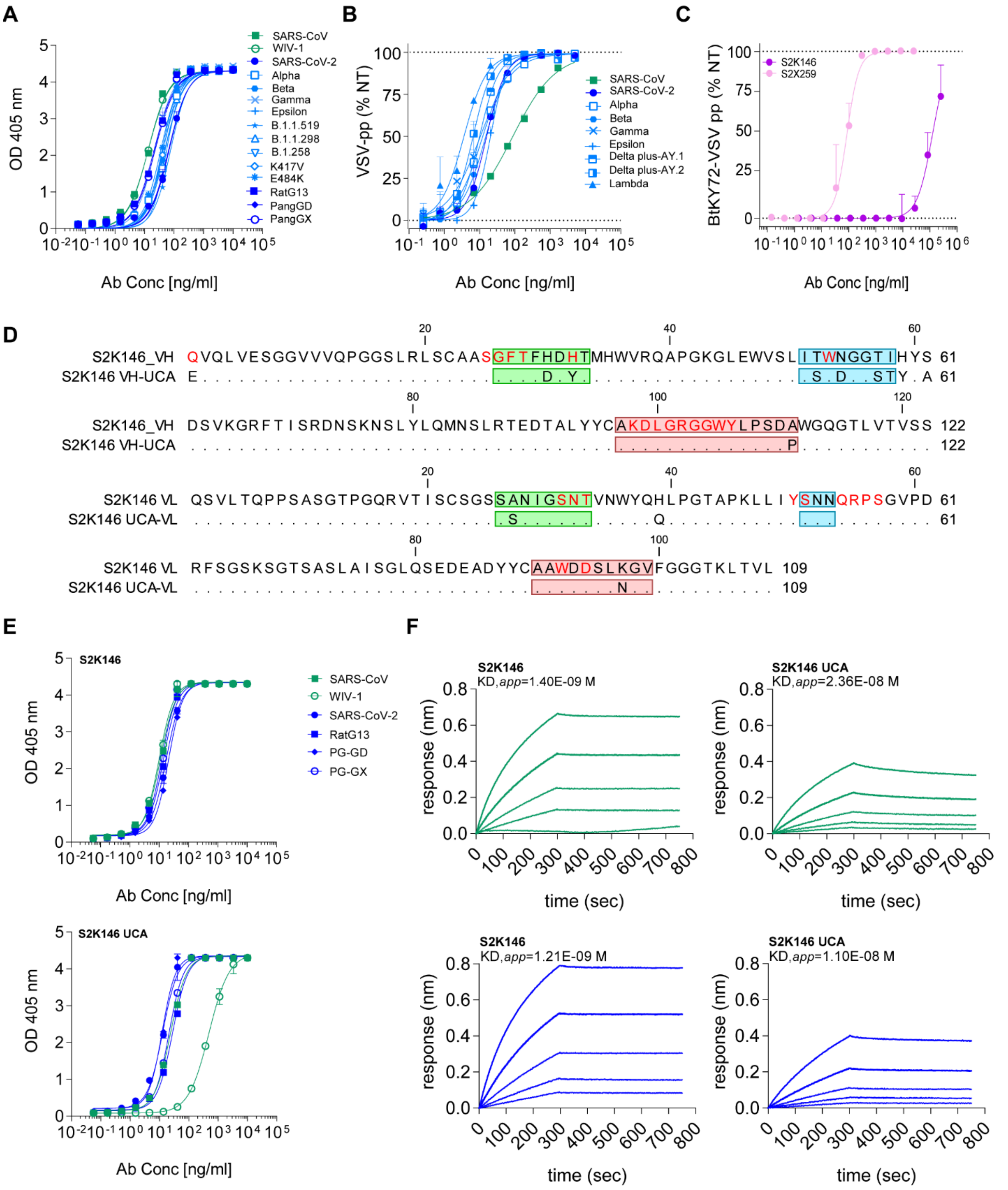
A
OD 405 nm
Ab Conc [ng/ml]
SARS-CoV
WIV-1
SARS-CoV-2
Alpha
Beta
Gamma
Epsilon
B.1.1.519
B.1.1.298
B.1.258
K417V
E484K
RatG13
PangGD
PangGX
B
VSV-pp (% NT)
Ab Conc [ng/ml]
SARS-CoV
SARS-CoV-2
Alpha
Beta
Gamma
Epsilon
Delta plus-AY.1
Delta plus-AY.2
Lambda
C
BtKY72-VSV pp (% NT)
Ab Conc [ng/ml]
S2K146
S2X259
D
S2K146_VH QVQLVESGGVVVQPGGSLRLSCAASGFTFHDHTMHWVRQAPGKGLEWVSLITWNGGTIHYS 61
S2K146 VH-UCA E . . . . D . Y . . . . S . D . . S T Y . A 61
S2K146_VH DSVKGRFTISRDNSKNSLYLQMNSLRTEDTALYYCAKDLGRGGWYLPSDAWGQGTLVTVSS 122
S2K146 VH-UCA . . . . P 122
S2K146 VL QSVLTQPPSASGTPGQRVTISCSGSSANIGSNTVNWYQHLPGTAPKLLIYSNNQRPSGVPD 61
S2K146 UCA-VL . . . . S . . . . Q 61
S2K146 VL RFSGSKSGTSASLAISGLQSEDEADYYCAAWDDSLKGVFGGGTKLTVL 109
S2K146 UCA-VL . . . . N 109
E
S2K146
S2K146 UCA
OD 405 nm
Ab Conc [ng/ml]
SARS-CoV
WIV-1
SARS-CoV-2
RatG13
PG-GD
PG-GX
F
S2K146
KD,app=1.40E-09 M
S2K146 UCA
KD,app=2.36E-08 M
S2K146
KD,app=1.21E-09 M
S2K146 UCA
KD,app=1.10E-08 M
response (nm)
time (sec)

Figure S2. In vitro characterization of the S2K146 mAb
A) S2K146 mAb binding to clade 1a and 1b sarbecovirus RBDs analyzed by ELISA. **B)** S2K146-mediated neutralization of VSV pseudotypes harboring Wuhan-Hu-1 SARS-CoV-2 S, B.1.351 (Beta) S, B.1.1.7 (Alpha) S, P.1 (Gamma) S, B.1.429 (Epsilon) S, C.37 (Lambda) S, AY.1/.2 (Delta+) S or SARS-CoV S. **C)** S2K146-mediated neutralization of VSV pseudotypes harboring BtKY72 S (K493Y/T498W, SARS-CoV-2 residue numbering (*34*)) using HEK293T cells stably expressing human ACE2 (*76*). The S2X259 mAb was included as a positive control. **D**) Alignment of the amino acid sequence of the variable regions of heavy and light chains of S2K146 and S2K146 UCA. Heavy and light chain CDR1, CDR2, and CDR3 (IMGT definition) are indicated by green, blue, and red boxes, respectively. Residues that make contact with the SARS-CoV-2 RBD are indicated in red. **E)** ELISA binding analysis of S2K146 and S2K146 UCA to sarbecovirus clade 1a and clade1b RBDs. **F)** Biolayer interferometry binding analysis of S2K146 and S2K146 UCA IgG1 to prefusion SARS-CoV S (green) and SARS-CoV-2 S (blue). Apparent K_D ($K_{D,app}$) are reported.

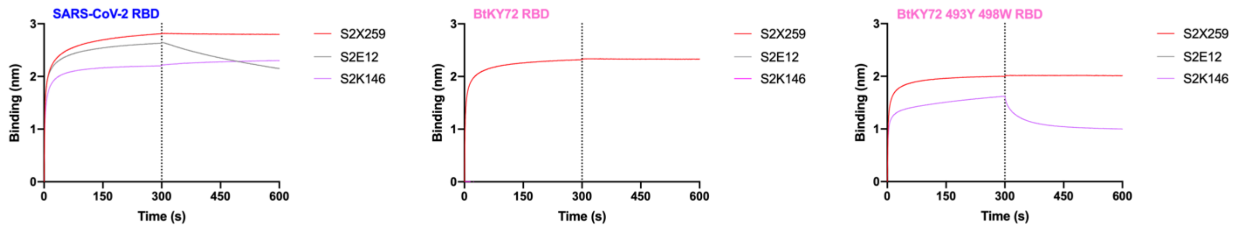

Figure S3. Evaluation of mAb binding to the BtKY72 RBD.
Binding of S2K146, S2E12 or S2X259 IgGs at 200 μg/mL to the SARS-CoV-2 Wuhan-Hu-1 RBD, the wildtype BtKY72 RBD or the BtKY72 K493Y/T498W RBD (defined based on SARS-CoV-2 numbering) immobilized at the surface of Ni-NTA biolayer interferometry biosensors.

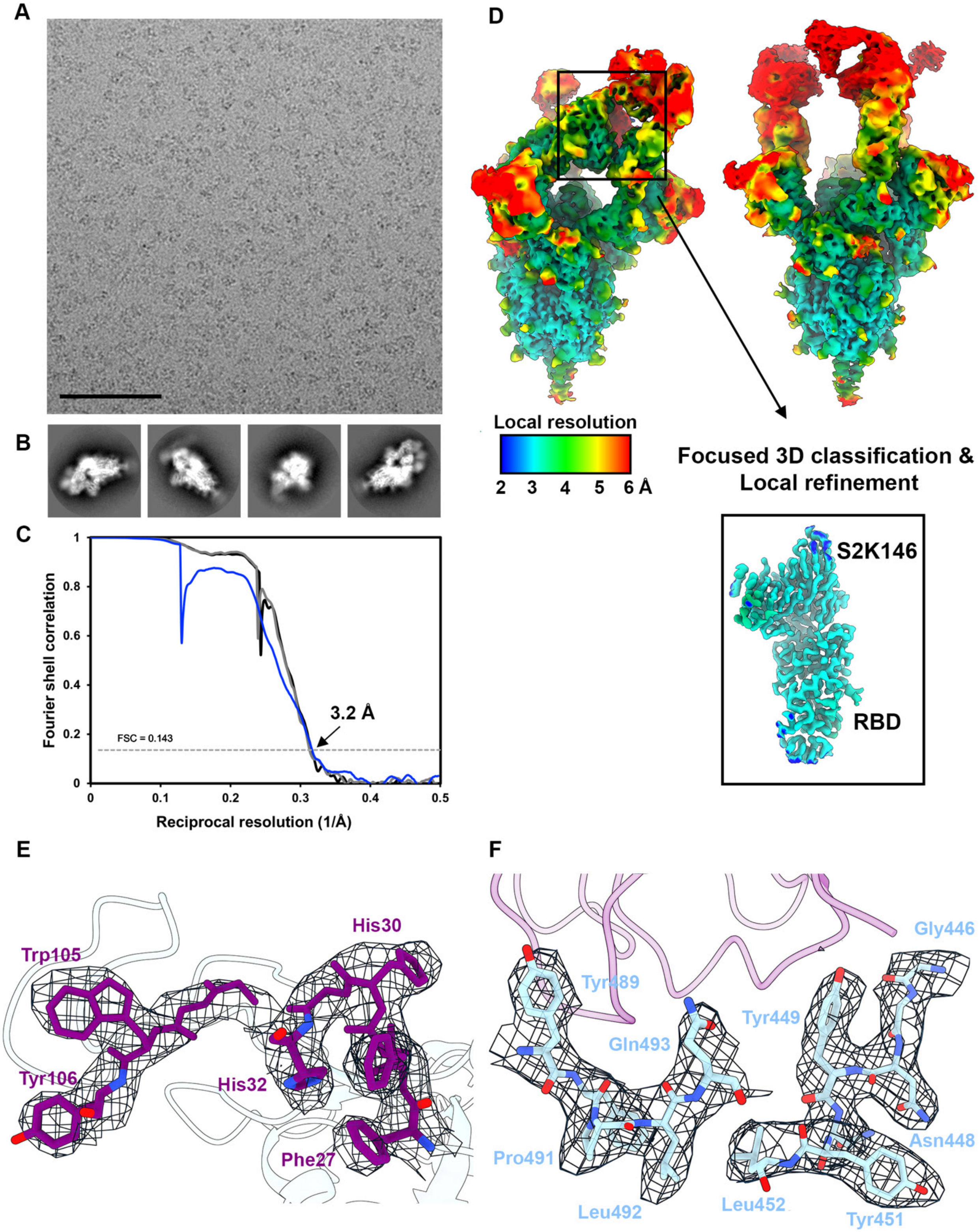
A
B
C
Fourier shell correlation
Reciprocal resolution (1/Å)
FSC = 0.143
3.2 Å
D
Local resolution
2 3 4 5 6 Å
Focused 3D classification & Local refinement
S2K146
RBD
E
Trp105
Tyr106
His30
His32
Phe27
F
Gly446
Tyr489
Tyr449
Gln493
Pro491
Leu492
Leu452
Asn448
Tyr451

Figure S4. CryoEM data processing of the S2K146-bound SARS-CoV-2 S dataset. A-B) Representative electron micrograph (A) and 2D class averages (B) of SARS-CoV-2 S in complex with the S2K146 Fab embedded in vitreous ice. The scale bar represents 100 nm. **C)** Gold-standard Fourier shell correlation curves for the S2K146-bound SARS-CoV-2 S maps with three RBDs open (black line), two RBDs open (grey line) and locally refined RBD/S2K146 variable domain (blue line). The 0.143 cutoff is indicated by a horizontal dashed line. **D)** Local resolution maps calculated using cryoSPARC for the whole SARS-CoV-2 S trimer reconstruction with two RBDs in the open state (left) and three RBDs in the open state (right) as well as for the locally refined RBD/S2K146 variable domain region (inset). **E-F)** CryoEM density (mesh representation) of the paratope (E) and epitope (F) with the corresponding model fit. The S2K146 variable heavy and light chains are shown in purple and magenta, respectively, whereas the SARS-CoV-2 RBD is light blue.

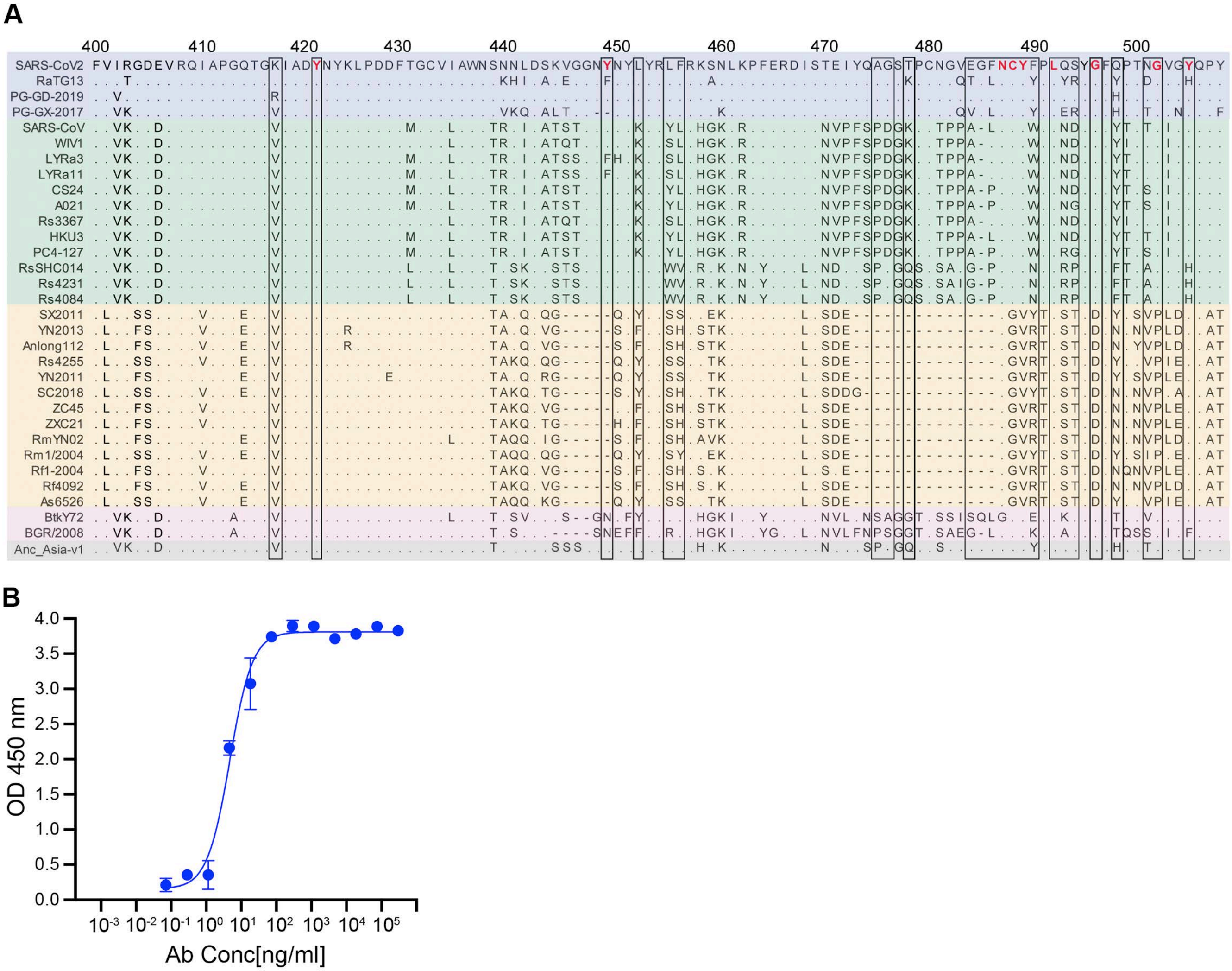

Figure S5. S2K146 epitope conservation across sarbecoviruses.

A) Protein sequence alignment of RBDs representative of sarbecovirus clade 1a (green), clade 1b (blue), clade 2 (orange) and clade 3 (pink) along with the reconstructed ancestor of all Asian sarbecoviruses (AncAsia) (*34*). Residue numbering is based on SARS-CoV-2 sequence with identical residues indicated as dots. Residues buried upon S2K146 binding are highlighted in boxes and epitope residues conserved between the SARS-CoV-2 and SARS-CoV RBDs are denoted in red.

B) Binding of S2K146 to the AncAsia RBD evaluated by ELISA.

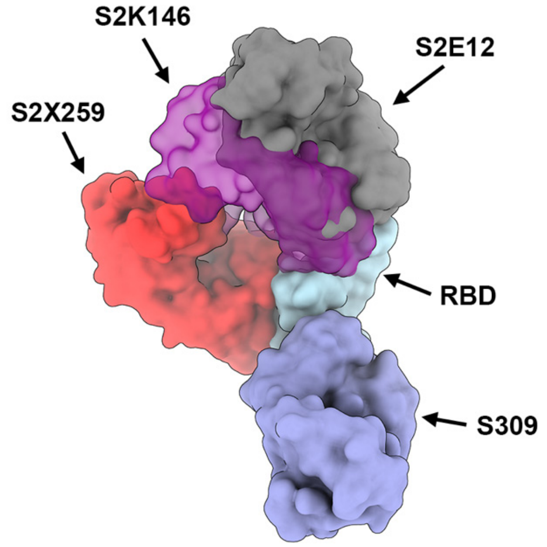

Figure S6. SARS-CoV-2 broadly neutralizing sarbecovirus mAbs.
Structural superimposition of S2K146 (purple), site I-targeting S2E12 (grey) (*33*), site II-targeting S2X259 (red) (*19*), and site IV-targeting S309 (blue) (*21*) Fabs bound to the SARS-CoV-2 RBD (light blue). S2K146 is depicted as a semi-transparent surface to show that S2E12 and S2K146 bind largely overlapping binding sites on the RBD. Although the constant domains of S2K146 and S2X259 slightly overlap, the flexibility around the Fab elbow allows binding of both mAbs as shown in Fig 1A.

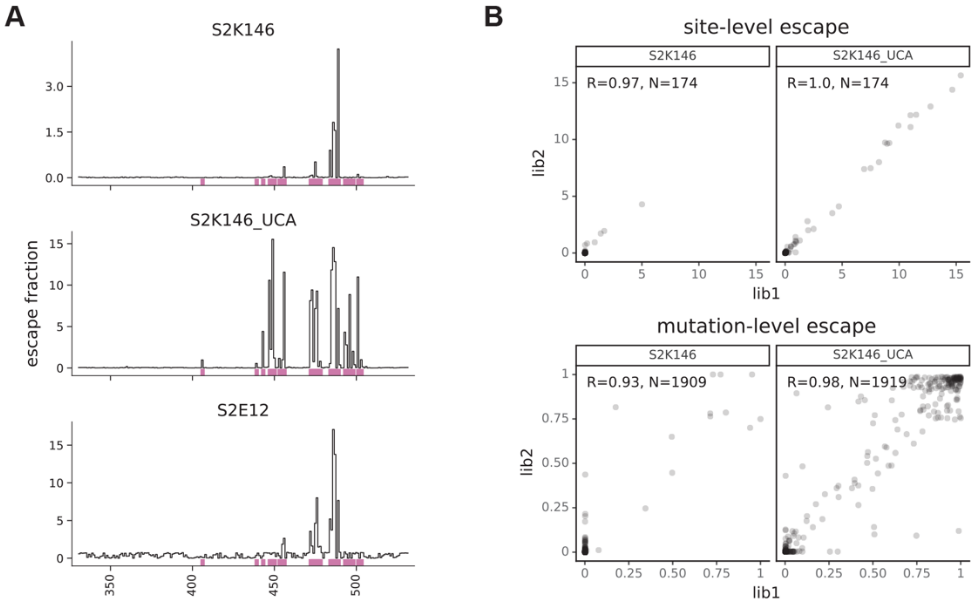

Figure S7. DMS analysis of the yeast-displayed SARS-CoV-2 RBD
A) Mapping of escape mutations reducing S2K146, S2K146 UCA or SE12 binding using yeast-displayed RBD DMS. Line plots show escape at each site in the RBD (summed effects of all mutations at each site). Sites of strong escape are indicated with purple lines. **B**) Correlation in per-mutation (down) and sum-per-site (up) escape fraction for replicate DMS library experiments.

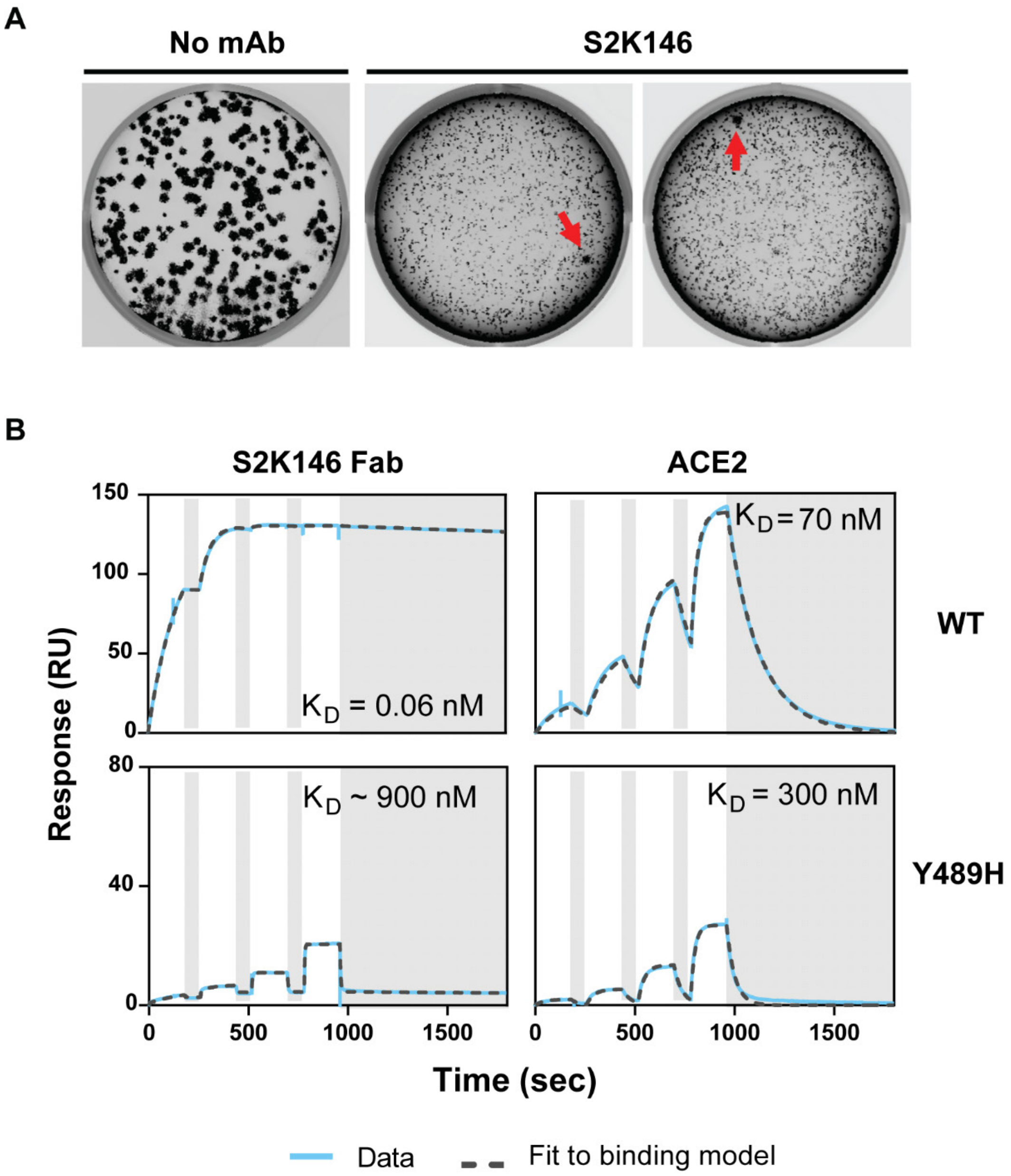

Figure S8. S2K146 escape clone selection by plaque assays using VSV-SARS-CoV-2 Wuhan-Hu-1 D614G S chimeric virus.

A) Plaque assays performed using VSV-SARS-CoV-2 Wuhan-Hu-1 D614G S chimeric virus on Vero cells with or without S2K146 in the overlay to isolate escape mutants (red arrow). Data are representative of two sets of experiments. **B)** Binding of the S2K146 Fab or monomeric human recombinant ACE2 ectodomain to the SARS-CoV-2 Wuhan-Hu-1 or Y489H RBD immobilized at the surface of SPR chips. The y axis is scaled based on RBD surface density. Experiments were performed with a 3-fold dilution series of Fab or monomeric hACE2: 300, 100, 33, 11 nM and were run as single-cycle kinetics.

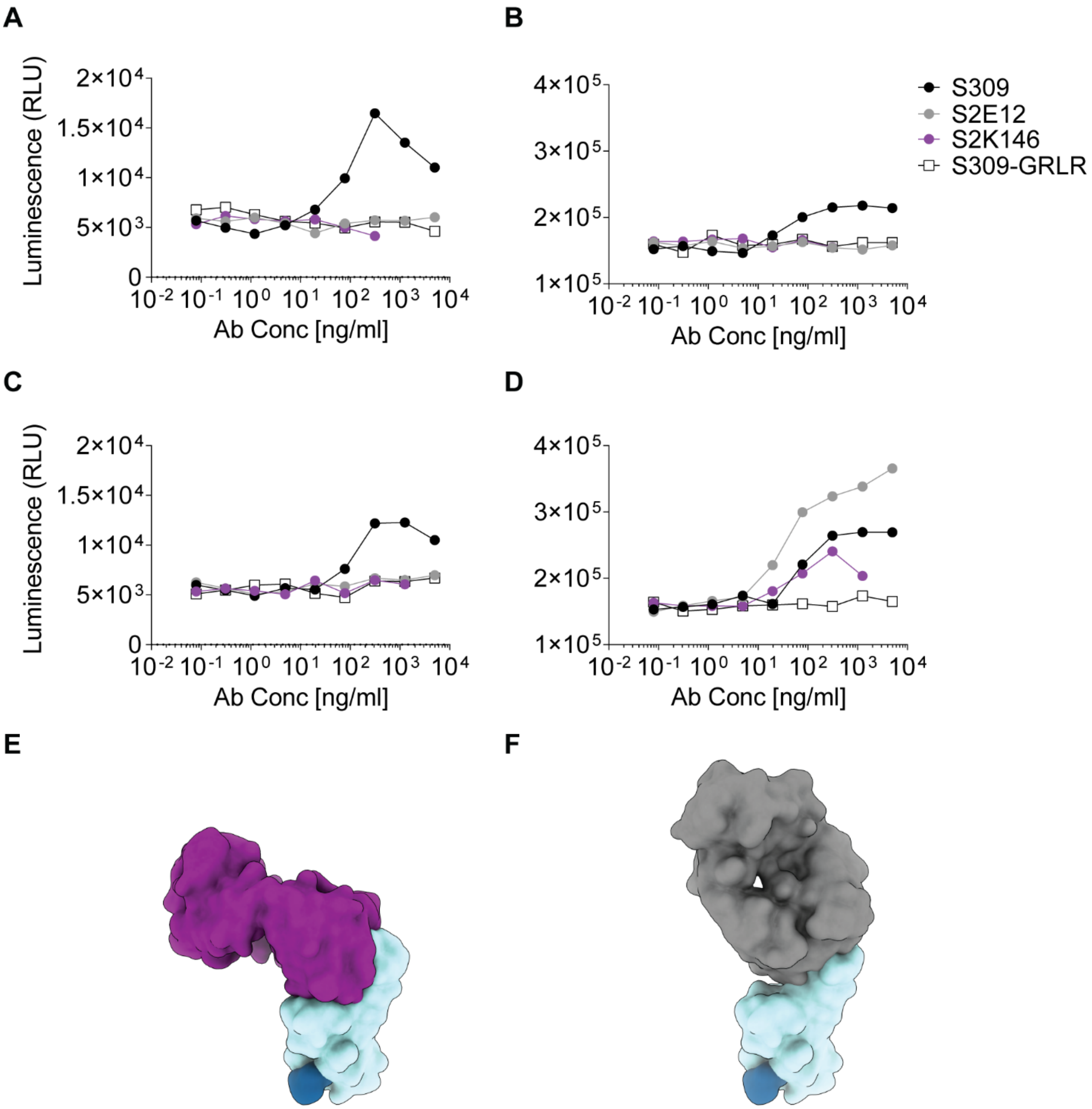

Figure S9. Activation of FcγRIIa and FcγRIIIa in vitro

A-B) NFAT-driven luciferase signal induced in Jurkat cells stably expressing FcγRIIa H131 variant (A) or FcγRIIIa V148 variant (B) upon S2K146 mAb binding to full-length wildtype SARS-CoV-2 S on CHO target cells. **C-D)** NFAT-driven luciferase signal induced in Jurkat cells stably expressing FcγRIIa H131 variant (C) or FcγRIIIa V148 variant (D) upon S2K146 mAb binding to uncleavable full-length wildtype SARS-CoV-2 S on CHO target cells. **E)** Surface rendering of site-I-targeting S2K146 (purple) mAb bound to the SARS-CoV-2 RBD (light blue). **F)** Surface rendering of site-I-targeting S2E12 (grey) mAb (*33*) bound to the SARS-CoV-2 RBD (light blue). The N343 glycan is rendered as a blue surface in E and F.

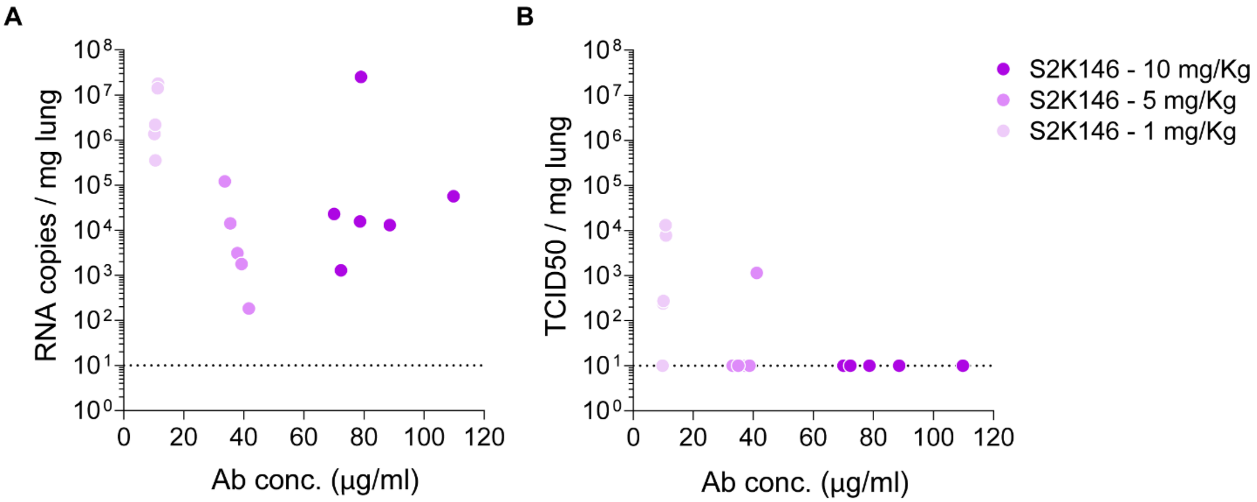

Figure S10. Correlation between mAb concentration and viral loads in the lungs of challenged hamsters. (A-B) Viral RNA copy number (A) and replicating virus titers (B) of SARS-CoV-2 Beta VOC in the lung of Syrian hamsters at 4 days post-infection plotted as a function of S2K146 mAb serum concentrations at day 1 post infection. (S2K146 10 mg/kg n = 6; S2K146 5 mg/kg n = 5; S2K146 1 mg/kg n = 5) was administered 1 day post infection.

	SARS-CoV-2 S/S2K146 (3RBDs open) EMD-25785	SARS-CoV-2 S/S2K146 (2RBDs open) PDB 7TAT EMD-25784	SARS-CoV-2 S RBD/S2K146 (Local refinement) PDB 7TAS EMD-25783
**Data collection and processing**			
Magnification	105,000	105,000	105,000
Voltage (kV)	300	300	300
Electron exposure ($e^{-}/Å^2$)	60	60	60
Defocus range (μm)	0.5-2.5	0.5-2.5	0.5-2.5
Pixel size (Å)	0.843	0.843	0.843
Symmetry imposed	C1	C1	C1
Final particle images (no.)	212,054	348,047	97,818
Map resolution (Å)	3.2	3.2	3.2
FSC threshold	0.143	0.143	0.143
Map sharpening *B* factor ($Å^2$)	-96	-99	-52
**Validation**			
MolProbity score		0.88	1.58
Clashscore		0.53	3.47
Poor rotamers (%)		0.18	1.69
Ramachandran plot			
Favored (%)		96.78	96.09
Allowed (%)		2.82	3.91
Disallowed (%)		0.4	0
EMRinger score		4.94	4.31

Table S1. CryoEM data collection and refinement statistics.

Clones	Nucleotide mutant	Amino acid Mutant
#1	T1465C/<br>T1602G	Y489H/<br>V534V
#2	T1465C	Y489H
#3	T1465C	Y489H
#4	T1465C	Y489H
#5	T1465C	Y489H
#6	T1465C	Y489H
#7	T1465C	Y489H
#8	T1465C	Y489H
#9	T1465C	Y489H
#10	T1465C	Y489H
#11	T1465C	Y489H
#12	T1465C	Y489H
#13	T1465C	Y489H
#14	T1465C	Y489H
#15	T1465C	Y489H
#16	T1465C	Y489H
#17	T1465C	Y489H
#18	T1465C	Y489H
#19	T1465C	Y489H
#20	T1465C	Y489H
#21	T1465C	Y489H
#22	T1465C	Y489H
#23	T1465C	Y489H
#24	T1465C	Y489H
#25	T1465C	Y489H
#26	T1465C	Y489H
#27	T1465C	Y489H
#28	T1464C/<br>T1465C	C488C/<br>Y489H
#29	T1465C	Y489H
#30	T1263C/<br>T1465C	Y421Y/<br>Y489H
#31	T1465C/<br>A1671G	Y489H/<br>K557K
#32	T1465C	Y489H
#33	T1465C	Y489H
#34	T1465C	Y489H
#35	T1465C	Y489H
#36	T1465C	Y489H

Table S2. Summary of nucleotide and amino acid mutations found in 36 neutralization-resistant VSV-SARS-CoV-2-S D614G chimera plaques.

References and Notes

1. C. A. Sánchez, H. Li, K. L. Phelps, C. Zambrana-Torrelio, L.-F. Wang, K. J. Olival, P. Daszak, A strategy to assess spillover risk of bat SARS-related coronaviruses in Southeast Asia. medRxiv 2021.09.09.21263359 [Preprint] (2021). https://doi.org/10.1101/2021.09.09.21263359.

2. A. C. Walls, M. A. Tortorici, B. J. Bosch, B. Frenz, P. J. M. Rottier, F. DiMaio, F. A. Rey, D. Veesler, Cryo-electron microscopy structure of a coronavirus spike glycoprotein trimer. *Nature* **531**, 114–117 (2016). doi:10.1038/nature16988 Medline

3. A. C. Walls, M. A. Tortorici, J. Snijder, X. Xiong, B. J. Bosch, F. A. Rey, D. Veesler, Tectonic conformational changes of a coronavirus spike glycoprotein promote membrane fusion. *Proc. Natl. Acad. Sci. U.S.A.* **114**, 11157–11162 (2017). doi:10.1073/pnas.1708727114 Medline

4. A. C. Walls, Y. J. Park, M. A. Tortorici, A. Wall, A. T. McGuire, D. Veesler, Structure, function, and antigenicity of the SARS-CoV-2 spike glycoprotein. *Cell* **181**, 281–292.e6 (2020). doi:10.1016/j.cell.2020.02.058 Medline

5. D. Wrapp, N. Wang, K. S. Corbett, J. A. Goldsmith, C. L. Hsieh, O. Abiona, B. S. Graham, J. S. McLellan, Cryo-EM structure of the 2019-nCoV spike in the prefusion conformation. *Science* **367**, 1260–1263 (2020). doi:10.1126/science.abb2507 Medline

6. M. A. Tortorici, D. Veesler, Structural insights into coronavirus entry. *Adv. Virus Res.* **105**, 93–116 (2019). doi:10.1016/bs.aivir.2019.08.002 Medline

7. L. Stamatatos, J. Czartoski, Y.-H. Wan, L. J. Homad, V. Rubin, H. Glantz, M. Neradilek, E. Seydoux, M. F. Jennewein, A. J. MacCamy, J. Feng, G. Mize, S. C. De Rosa, A. Finzi, M. P. Lemos, K. W. Cohen, Z. Moodie, M. J. McElrath, A. T. McGuire, mRNA vaccination boosts cross-variant neutralizing antibodies elicited by SARS-CoV-2 infection. *Science* **372**, 1413–1418 (2021). doi:10.1126/science.abg9175 Medline

8. P. Zhou, X. L. Yang, X. G. Wang, B. Hu, L. Zhang, W. Zhang, H. R. Si, Y. Zhu, B. Li, C. L. Huang, H. D. Chen, J. Chen, Y. Luo, H. Guo, R. D. Jiang, M. Q. Liu, Y. Chen, X. R. Shen, X. Wang, X. S. Zheng, K. Zhao, Q. J. Chen, F. Deng, L. L. Liu, B. Yan, F. X. Zhan, Y. Y. Wang, G. F. Xiao, Z. L. Shi, A pneumonia outbreak associated with a new coronavirus of probable bat origin. *Nature* **579**, 270–273 (2020). doi:10.1038/s41586-020-2012-7 Medline

9. M. Letko, A. Marzi, V. Munster, Functional assessment of cell entry and receptor usage for SARS-CoV-2 and other lineage B betacoronaviruses. *Nat. Microbiol.* **5**, 562–569 (2020). doi:10.1038/s41564-020-0688-y Medline

10. M. Hoffmann, H. Kleine-Weber, S. Schroeder, N. Krüger, T. Herrler, S. Erichsen, T. S. Schiergens, G. Herrler, N. H. Wu, A. Nitsche, M. A. Müller, C. Drosten, S. Pöhlmann, SARS-CoV-2 cell entry depends on ACE2 and TMPRSS2 and is blocked by a clinically proven protease inhibitor. *Cell* **181**, 271–280.e8 (2020). doi:10.1016/j.cell.2020.02.052 Medline

11. W. Li, M. J. Moore, N. Vasilieva, J. Sui, S. K. Wong, M. A. Berne, M. Somasundaran, J. L. Sullivan, K. Luzuriaga, T. C. Greenough, H. Choe, M. Farzan, Angiotensin-converting

enzyme 2 is a functional receptor for the SARS coronavirus. *Nature* **426**, 450–454 (2003). doi:10.1038/nature02145 Medline

12. F. Li, W. Li, M. Farzan, S. C. Harrison, Structure of SARS coronavirus spike receptor-binding domain complexed with receptor. *Science* **309**, 1864–1868 (2005). doi:10.1126/science.1116480 Medline

13. Q. Wang, Y. Zhang, L. Wu, S. Niu, C. Song, Z. Zhang, G. Lu, C. Qiao, Y. Hu, K.-Y. Yuen, Q. Wang, H. Zhou, J. Yan, J. Qi, Structural and functional basis of SARS-CoV-2 entry by using human ACE2. *Cell* **181**, 894–904.e9 (2020). doi:10.1016/j.cell.2020.03.045 Medline

14. R. Yan, Y. Zhang, Y. Li, L. Xia, Y. Guo, Q. Zhou, Structural basis for the recognition of SARS-CoV-2 by full-length human ACE2. *Science* **367**, 1444–1448 (2020). doi:10.1126/science.abb2762 Medline

15. J. Shang, G. Ye, K. Shi, Y. Wan, C. Luo, H. Aihara, Q. Geng, A. Auerbach, F. Li, Structural basis of receptor recognition by SARS-CoV-2. *Nature* **581**, 221–224 (2020). doi:10.1038/s41586-020-2179-y Medline

16. J. Lan, J. Ge, J. Yu, S. Shan, H. Zhou, S. Fan, Q. Zhang, X. Shi, Q. Wang, L. Zhang, X. Wang, Structure of the SARS-CoV-2 spike receptor-binding domain bound to the ACE2 receptor. *Nature* **581**, 215–220 (2020). doi:10.1038/s41586-020-2180-5 Medline

17. L. Piccoli, Y. J. Park, M. A. Tortorici, N. Czudnochowski, A. C. Walls, M. Beltramello, C. Silacci-Fregni, D. Pinto, L. E. Rosen, J. E. Bowen, O. J. Acton, S. Jaconi, B. Guarino, A. Minola, F. Zatta, N. Sprugasci, J. Bassi, A. Peter, A. De Marco, J. C. Nix, F. Mele, S. Jovic, B. F. Rodriguez, S. V. Gupta, F. Jin, G. Piumatti, G. Lo Presti, A. F. Pellanda, M. Biggiogero, M. Tarkowski, M. S. Pizzuto, E. Cameroni, C. Havenar-Daughton, M. Smithey, D. Hong, V. Lepori, E. Albanese, A. Ceschi, E. Bernasconi, L. Elzi, P. Ferrari, C. Garzoni, A. Riva, G. Snell, F. Sallusto, K. Fink, H. W. Virgin, A. Lanzavecchia, D. Corti, D. Veesler, Mapping neutralizing and immunodominant sites on the SARS-CoV-2 spike receptor-binding domain by structure-guided high-resolution serology. *Cell* **183**, 1024–1042.e21 (2020). doi:10.1016/j.cell.2020.09.037 Medline

18. A. J. Greaney, A. N. Loes, L. E. Gentles, K. H. D. Crawford, T. N. Starr, K. D. Malone, H. Y. Chu, J. D. Bloom, Antibodies elicited by mRNA-1273 vaccination bind more broadly to the receptor binding domain than do those from SARS-CoV-2 infection. *Sci. Transl. Med.* **13**, eabi9915 (2021). doi:10.1126/scitranslmed.abi9915 Medline

19. M. A. Tortorici, N. Czudnochowski, T. N. Starr, R. Marzi, A. C. Walls, F. Zatta, J. E. Bowen, S. Jaconi, J. Di Iulio, Z. Wang, A. De Marco, S. K. Zepeda, D. Pinto, Z. Liu, M. Beltramello, I. Bartha, M. P. Housley, F. A. Lempp, L. E. Rosen, E. Dellota Jr., H. Kaiser, M. Montiel-Ruiz, J. Zhou, A. Addetia, B. Guarino, K. Culap, N. Sprugasci, C. Saliba, E. Vetti, I. Giacchetto-Sasselli, C. S. Fregni, R. Abdelnabi, S. C. Foo, C. Havenar-Daughton, M. A. Schmid, F. Benigni, E. Cameroni, J. Neyts, A. Telenti, H. W. Virgin, S. P. J. Whelan, G. Snell, J. D. Bloom, D. Corti, D. Veesler, M. S. Pizzuto, Broad sarbecovirus neutralization by a human monoclonal antibody. *Nature* **597**, 103–108 (2021). doi:10.1038/s41586-021-03817-4 Medline

20. T. N. Starr, N. Czudnochowski, Z. Liu, F. Zatta, Y.-J. Park, A. Addetia, D. Pinto, M. Beltramello, P. Hernandez, A. J. Greaney, R. Marzi, W. G. Glass, I. Zhang, A. S.

Dingens, J. E. Bowen, M. A. Tortorici, A. C. Walls, J. A. Wojcechowskyj, A. De Marco, L. E. Rosen, J. Zhou, M. Montiel-Ruiz, H. Kaiser, J. R. Dillen, H. Tucker, J. Bassi, C. Silacci-Fregni, M. P. Housley, J. di Iulio, G. Lombardo, M. Agostini, N. Sprugasci, K. Culap, S. Jaconi, M. Meury, E. Dellota Jr., R. Abdelnabi, S. C. Foo, E. Cameroni, S. Stumpf, T. I. Croll, J. C. Nix, C. Havenar-Daughton, L. Piccoli, F. Benigni, J. Neyts, A. Telenti, F. A. Lempp, M. S. Pizzuto, J. D. Chodera, C. M. Hebner, H. W. Virgin, S. P. J. Whelan, D. Veesler, D. Corti, J. D. Bloom, G. Snell, SARS-CoV-2 RBD antibodies that maximize breadth and resistance to escape. *Nature* **597**, 97–102 (2021). doi:10.1038/s41586-021-03807-6 Medline

21. D. Pinto, Y. J. Park, M. Beltramello, A. C. Walls, M. A. Tortorici, S. Bianchi, S. Jaconi, K. Culap, F. Zatta, A. De Marco, A. Peter, B. Guarino, R. Spreafico, E. Cameroni, J. B. Case, R. E. Chen, C. Havenar-Daughton, G. Snell, A. Telenti, H. W. Virgin, A. Lanzavecchia, M. S. Diamond, K. Fink, D. Veesler, D. Corti, Cross-neutralization of SARS-CoV-2 by a human monoclonal SARS-CoV antibody. *Nature* **583**, 290–295 (2020). doi:10.1038/s41586-020-2349-y Medline

22. C. A. Jette, A. A. Cohen, P. N. P. Gnanapragasam, F. Muecksch, Y. E. Lee, K. E. Huey-Tubman, F. Schmidt, T. Hatziioannou, P. D. Bieniasz, M. C. Nussenzweig, A. P. West Jr., J. R. Keeffe, P. J. Bjorkman, C. O. Barnes, Broad cross-reactivity across sarbecoviruses exhibited by a subset of COVID-19 donor-derived neutralizing antibodies. *Cell Rep.* **36**, 109760 (2021). doi:10.1016/j.celrep.2021.109760

23. D. R. Martinez, A. Schaefer, S. Gobeil, D. Li, G. De la Cruz, R. Parks, X. Lu, M. Barr, K. Manne, K. Mansouri, R. J. Edwards, B. Yount, K. Anasti, S. A. Montgomery, S. Shen, T. Zhou, P. D. Kwong, B. S. Graham, J. R. Mascola, D. C. Montefiori, M. Alam, G. D. Sempowski, K. Wiehe, K. O. Saunders, P. Acharya, B. F. Haynes, R. S. Baric, A broadly neutralizing antibody protects against SARS-CoV, pre-emergent bat CoVs, and SARS-CoV-2 variants in mice. bioRxiv 2021.04.27.441655 [Preprint] (2021). https://doi.org/10.1101/2021.04.27.441655.

24. A. Z. Wec, D. Wrapp, A. S. Herbert, D. P. Maurer, D. Haslwanter, M. Sakharkar, R. K. Jangra, M. E. Dieterle, A. Lilov, D. Huang, L. V. Tse, N. V. Johnson, C. L. Hsieh, N. Wang, J. H. Nett, E. Champney, I. Burnina, M. Brown, S. Lin, M. Sinclair, C. Johnson, S. Pudi, R. Bortz 3rd, A. S. Wirchnianski, E. Laudermilch, C. Florez, J. M. Fels, C. M. O'Brien, B. S. Graham, D. Nemazee, D. R. Burton, R. S. Baric, J. E. Voss, K. Chandran, J. M. Dye, J. S. McLellan, L. M. Walker, Broad neutralization of SARS-related viruses by human monoclonal antibodies. *Science* **369**, 731–736 (2020). doi:10.1126/science.abc7424 Medline

25. C. G. Rappazzo, L. V. Tse, C. I. Kaku, D. Wrapp, M. Sakharkar, D. Huang, L. M. Deveau, T. J. Yockachonis, A. S. Herbert, M. B. Battles, C. M. O'Brien, M. E. Brown, J. C. Geoghegan, J. Belk, L. Peng, L. Yang, Y. Hou, T. D. Scobey, D. R. Burton, D. Nemazee, J. M. Dye, J. E. Voss, B. M. Gunn, J. S. McLellan, R. S. Baric, L. E. Gralinski, L. M. Walker, Broad and potent activity against SARS-like viruses by an engineered human monoclonal antibody. *Science* **371**, 823–829 (2021). doi:10.1126/science.abf4830 Medline

26. D. Corti, L. A. Purcell, G. Snell, D. Veesler, Tackling COVID-19 with neutralizing monoclonal antibodies. *Cell* **184**, 3086–3108 (2021). doi:10.1016/j.cell.2021.05.005 Medline

27. M. McCallum, J. Bassi, A. De Marco, A. Chen, A. C. Walls, J. Di Iulio, M. A. Tortorici, M.-J. Navarro, C. Silacci-Fregni, C. Saliba, K. R. Sprouse, M. Agostini, D. Pinto, K. Culap, S. Bianchi, S. Jaconi, E. Cameroni, J. E. Bowen, S. W. Tilles, M. S. Pizzuto, S. B. Guastalla, G. Bona, A. F. Pellanda, C. Garzoni, W. C. Van Voorhis, L. E. Rosen, G. Snell, A. Telenti, H. W. Virgin, L. Piccoli, D. Corti, D. Veesler, SARS-CoV-2 immune evasion by the B.1.427/B.1.429 variant of concern. *Science* **373**, 648–654 (2021). doi:10.1126/science.abi7994 Medline

28. M. McCallum, A. C. Walls, K. R. Sprouse, J. E. Bowen, L. E. Rosen, H. V. Dang, A. De Marco, N. Franko, S. W. Tilles, J. Logue, M. C. Miranda, M. Ahlrichs, L. Carter, G. Snell, M. S. Pizzuto, H. Y. Chu, W. C. Van Voorhis, D. Corti, D. Veesler, Molecular basis of immune evasion by the Delta and Kappa SARS-CoV-2 variants. *Science* **374**, 1621–1626 (2021). doi:10.1126/science.abl8506 Medline

29. P. Mlcochova, S. A. Kemp, M. S. Dhar, G. Papa, B. Meng, I. A. T. M. Ferreira, R. Datir, D. A. Collier, A. Albecka, S. Singh, R. Pandey, J. Brown, J. Zhou, N. Goonawardane, S. Mishra, C. Whittaker, T. Mellan, R. Marwal, M. Datta, S. Sengupta, K. Ponnusamy, V. S. Radhakrishnan, A. Abdullahi, O. Charles, P. Chattopadhyay, P. Devi, D. Caputo, T. Peacock, C. Wattal, N. Goel, A. Satwik, R. Vaishya, M. Agarwal, A. Mavousian, J. H. Lee, J. Bassi, C. Silacci-Fegni, C. Saliba, D. Pinto, T. Irie, I. Yoshida, W. L. Hamilton, K. Sato, S. Bhatt, S. Flaxman, L. C. James, D. Corti, L. Piccoli, W. S. Barclay, P. Rakshit, A. Agrawal, R. K. Gupta; Indian SARS-CoV-2 Genomics Consortium (INSACOG); Genotype to Phenotype Japan (G2P-Japan) Consortium; CITIID-NIHR BioResource COVID-19 Collaboration, SARS-CoV-2 B.1.617.2 Delta variant replication and immune evasion. *Nature* **599**, 114–119 (2021). doi:10.1038/s41586-021-03944-y Medline

30. T. N. Starr, A. J. Greaney, S. K. Hilton, D. Ellis, K. H. D. Crawford, A. S. Dingens, M. J. Navarro, J. E. Bowen, M. A. Tortorici, A. C. Walls, N. P. King, D. Veesler, J. D. Bloom, Deep mutational scanning of SARS-CoV-2 receptor binding domain reveals constraints on folding and ACE2 binding. *Cell* **182**, 1295–1310.e20 (2020). doi:10.1016/j.cell.2020.08.012 Medline

31. T. N. Starr, A. J. Greaney, A. S. Dingens, J. D. Bloom, Complete map of SARS-CoV-2 RBD mutations that escape the monoclonal antibody LY-CoV555 and its cocktail with LY-CoV016. *Cell Rep. Med.* **2**, 100255 (2021). doi:10.1016/j.xcrm.2021.100255 Medline

32. T. N. Starr, A. J. Greaney, A. Addetia, W. W. Hannon, M. C. Choudhary, A. S. Dingens, J. Z. Li, J. D. Bloom, Prospective mapping of viral mutations that escape antibodies used to treat COVID-19. *Science* **371**, 850–854 (2021). doi:10.1126/science.abf9302 Medline

33. M. A. Tortorici, M. Beltramello, F. A. Lempp, D. Pinto, H. V. Dang, L. E. Rosen, M. McCallum, J. Bowen, A. Minola, S. Jaconi, F. Zatta, A. De Marco, B. Guarino, S. Bianchi, E. J. Lauron, H. Tucker, J. Zhou, A. Peter, C. Havenar-Daughton, J. A. Wojcechowskyj, J. B. Case, R. E. Chen, H. Kaiser, M. Montiel-Ruiz, M. Meury, N. Czudnochowski, R. Spreafico, J. Dillen, C. Ng, N. Sprugasci, K. Culap, F. Benigni, R.

Abdelnabi, S. C. Foo, M. A. Schmid, E. Cameroni, A. Riva, A. Gabrieli, M. Galli, M. S. Pizzuto, J. Neyts, M. S. Diamond, H. W. Virgin, G. Snell, D. Corti, K. Fink, D. Veesler, Ultrapotent human antibodies protect against SARS-CoV-2 challenge via multiple mechanisms. *Science* **370**, 950–957 (2020). doi:10.1126/science.abe3354 Medline

34. T. N. Starr, S. K. Zepeda, A. C. Walls, A. J. Greaney, D. Veesler, J. D. Bloom, ACE2 binding is an ancestral and evolvable trait of sarbecoviruses. bioRxiv 2021.07.17.452804 [Preprint] (2021). https://doi.org/10.1101/2021.07.17.452804.

35. J. B. Case, P. W. Rothlauf, R. E. Chen, Z. Liu, H. Zhao, A. S. Kim, L. M. Bloyet, Q. Zeng, S. Tahan, L. Droit, M. X. G. Ilagan, M. A. Tartell, G. Amarasinghe, J. P. Henderson, S. Miersch, M. Ustav, S. Sidhu, H. W. Virgin, D. Wang, S. Ding, D. Corti, E. S. Theel, D. H. Fremont, M. S. Diamond, S. P. J. Whelan, Neutralizing antibody and soluble ACE2 inhibition of a replication-competent VSV-SARS-CoV-2 and a clinical isolate of SARS-CoV-2. *Cell Host Microbe* **28**, 475–485.e5 (2020). doi:10.1016/j.chom.2020.06.021 Medline

36. A. C. Walls, X. Xiong, Y. J. Park, M. A. Tortorici, J. Snijder, J. Quispe, E. Cameroni, R. Gopal, M. Dai, A. Lanzavecchia, M. Zambon, F. A. Rey, D. Corti, D. Veesler, Unexpected receptor functional mimicry elucidates activation of coronavirus fusion. *Cell* **176**, 1026–1039.e15 (2019). doi:10.1016/j.cell.2018.12.028 Medline

37. F. A. Lempp, L. B. Soriaga, M. Montiel-Ruiz, F. Benigni, J. Noack, Y.-J. Park, S. Bianchi, A. C. Walls, J. E. Bowen, J. Zhou, H. Kaiser, A. Joshi, M. Agostini, M. Meury, E. Dellota Jr., S. Jaconi, E. Cameroni, J. Martinez-Picado, J. Vergara-Alert, N. Izquierdo-Useros, H. W. Virgin, A. Lanzavecchia, D. Veesler, L. A. Purcell, A. Telenti, D. Corti, Lectins enhance SARS-CoV-2 infection and influence neutralizing antibodies. *Nature* **598**, 342–347 (2021). doi:10.1038/s41586-021-03925-1 Medline

38. J. Huo, Y. Zhao, J. Ren, D. Zhou, H. M. E. Duyvesteyn, H. M. Ginn, L. Carrique, T. Malinauskas, R. R. Ruza, P. N. M. Shah, T. K. Tan, P. Rijal, N. Coombes, K. R. Bewley, J. A. Tree, J. Radecke, N. G. Paterson, P. Supasa, J. Mongkolsapaya, G. R. Screaton, M. Carroll, A. Townsend, E. E. Fry, R. J. Owens, D. I. Stuart, Neutralization of SARS-CoV-2 by destruction of the prefusion spike. *Cell Host Microbe* **28**, 445–454.e6 (2020). doi:10.1016/j.chom.2020.06.010 Medline

39. R. Abdelnabi, R. Boudewijns, C. S. Foo, L. Seldeslachts, L. Sanchez-Felipe, X. Zhang, L. Delang, P. Maes, S. J. F. Kaptein, B. Weynand, G. Vande Velde, J. Neyts, K. Dallmeier, Comparing infectivity and virulence of emerging SARS-CoV-2 variants in Syrian hamsters. *EBioMedicine* **68**, 103403 (2021). doi:10.1016/j.ebiom.2021.103403 Medline

40. R. Boudewijns, H. J. Thibaut, S. J. F. Kaptein, R. Li, V. Vergote, L. Seldeslachts, J. Van Weyenbergh, C. De Keyzer, L. Bervoets, S. Sharma, L. Liesenborghs, J. Ma, S. Jansen, D. Van Looveren, T. Vercruysse, X. Wang, D. Jochmans, E. Martens, K. Roose, D. De Vlieger, B. Schepens, T. Van Buyten, S. Jacobs, Y. Liu, J. Martí-Carreras, B. Vanmechelen, T. Wawina-Bokalanga, L. Delang, J. Rocha-Pereira, L. Coelmont, W. Chiu, P. Leyssen, E. Heylen, D. Schols, L. Wang, L. Close, J. Matthijnssens, M. Van Ranst, V. Compernolle, G. Schramm, K. Van Laere, X. Saelens, N. Callewaert, G. Opdenakker, P. Maes, B. Weynand, C. Cawthorne, G. Vande Velde, Z. Wang, J. Neyts, K. Dallmeier, STAT2 signaling restricts viral dissemination but drives severe pneumonia

in SARS-CoV-2 infected hamsters. *Nat. Commun.* **11**, 5838 (2020). doi:10.1038/s41467-020-19684-y Medline

41. W. Dejnirattisai, D. Zhou, H. M. Ginn, H. M. E. Duyvesteyn, P. Supasa, J. B. Case, Y. Zhao, T. S. Walter, A. J. Mentzer, C. Liu, B. Wang, G. C. Paesen, J. Slon-Campos, C. López-Camacho, N. M. Kafai, A. L. Bailey, R. E. Chen, B. Ying, C. Thompson, J. Bolton, A. Fyfe, S. Gupta, T. K. Tan, J. Gilbert-Jaramillo, W. James, M. Knight, M. W. Carroll, D. Skelly, C. Dold, Y. Peng, R. Levin, T. Dong, A. J. Pollard, J. C. Knight, P. Klenerman, N. Temperton, D. R. Hall, M. A. Williams, N. G. Paterson, F. K. R. Bertram, C. A. Siebert, D. K. Clare, A. Howe, J. Radecke, Y. Song, A. R. Townsend, K.-Y. A. Huang, E. E. Fry, J. Mongkolsapaya, M. S. Diamond, J. Ren, D. I. Stuart, G. R. Screaton, The antigenic anatomy of SARS-CoV-2 receptor binding domain. *Cell* **184**, 2183–2200.e22 (2021). doi:10.1016/j.cell.2021.02.032 Medline

42. P. S. Arunachalam, A. C. Walls, N. Golden, C. Atyeo, S. Fischinger, C. Li, P. Aye, M. J. Navarro, L. Lai, V. V. Edara, K. Röltgen, K. Rogers, L. Shirreff, D. E. Ferrell, S. Wrenn, D. Pettie, J. C. Kraft, M. C. Miranda, E. Kepl, C. Sydeman, N. Brunette, M. Murphy, B. Fiala, L. Carter, A. G. White, M. Trisal, C.-L. Hsieh, K. Russell-Lodrigue, C. Monjure, J. Dufour, S. Spencer, L. Doyle-Meyers, R. P. Bohm, N. J. Maness, C. Roy, J. A. Plante, K. S. Plante, A. Zhu, M. J. Gorman, S. Shin, X. Shen, J. Fontenot, S. Gupta, D. T. O'Hagan, R. Van Der Most, R. Rappuoli, R. L. Coffman, D. Novack, J. S. McLellan, S. Subramaniam, D. Montefiori, S. D. Boyd, J. A. L. Flynn, G. Alter, F. Villinger, H. Kleanthous, J. Rappaport, M. S. Suthar, N. P. King, D. Veesler, B. Pulendran, Adjuvanting a subunit COVID-19 vaccine to induce protective immunity. *Nature* **594**, 253–258 (2021). doi:10.1038/s41586-021-03530-2 Medline

43. A. C. Walls, B. Fiala, A. Schäfer, S. Wrenn, M. N. Pham, M. Murphy, L. V. Tse, L. Shehata, M. A. O'Connor, C. Chen, M. J. Navarro, M. C. Miranda, D. Pettie, R. Ravichandran, J. C. Kraft, C. Ogohara, A. Palser, S. Chalk, E. C. Lee, K. Guerriero, E. Kepl, C. M. Chow, C. Sydeman, E. A. Hodge, B. Brown, J. T. Fuller, K. H. Dinnon 3rd, L. E. Gralinski, S. R. Leist, K. L. Gully, T. B. Lewis, M. Guttman, H. Y. Chu, K. K. Lee, D. H. Fuller, R. S. Baric, P. Kellam, L. Carter, M. Pepper, T. P. Sheahan, D. Veesler, N. P. King, Elicitation of potent neutralizing antibody responses by designed protein nanoparticle vaccines for SARS-CoV-2. *Cell* **183**, 1367–1382.e17 (2020). doi:10.1016/j.cell.2020.10.043 Medline

44. A. C. Walls, M. C. Miranda, A. Schäfer, M. N. Pham, A. Greaney, P. S. Arunachalam, M.-J. Navarro, M. A. Tortorici, K. Rogers, M. A. O'Connor, L. Shirreff, D. E. Ferrell, J. Bowen, N. Brunette, E. Kepl, S. K. Zepeda, T. Starr, C.-L. Hsieh, B. Fiala, S. Wrenn, D. Pettie, C. Sydeman, K. R. Sprouse, M. Johnson, A. Blackstone, R. Ravichandran, C. Ogohara, L. Carter, S. W. Tilles, R. Rappuoli, S. R. Leist, D. R. Martinez, M. Clark, R. Tisch, D. T. O'Hagan, R. Van Der Most, W. C. Van Voorhis, D. Corti, J. S. McLellan, H. Kleanthous, T. P. Sheahan, K. D. Smith, D. H. Fuller, F. Villinger, J. Bloom, B. Pulendran, R. S. Baric, N. P. King, D. Veesler, Elicitation of broadly protective sarbecovirus immunity by receptor-binding domain nanoparticle vaccines. *Cell* **184**, 5432–5447.e16 (2021). doi:10.1016/j.cell.2021.09.015 Medline

45. K. O. Saunders, E. Lee, R. Parks, D. R. Martinez, D. Li, H. Chen, R. J. Edwards, S. Gobeil, M. Barr, K. Mansouri, S. M. Alam, L. L. Sutherland, F. Cai, A. M. Sanzone, M. Berry, K. Manne, K. W. Bock, M. Minai, B. M. Nagata, A. B. Kapingidza, M. Azoitei, L. V.

Tse, T. D. Scobey, R. L. Spreng, R. W. Rountree, C. T. DeMarco, T. N. Denny, C. W. Woods, E. W. Petzold, J. Tang, T. H. Oguin 3rd, G. D. Sempowski, M. Gagne, D. C. Douek, M. A. Tomai, C. B. Fox, R. Seder, K. Wiehe, D. Weissman, N. Pardi, H. Golding, S. Khurana, P. Acharya, H. Andersen, M. G. Lewis, I. N. Moore, D. C. Montefiori, R. S. Baric, B. F. Haynes, Neutralizing antibody vaccine for pandemic and pre-emergent coronaviruses. *Nature* **594**, 553–559 (2021). doi:10.1038/s41586-021-03594-0 Medline

46. D. R. Martinez, A. Schäfer, S. R. Leist, G. De la Cruz, A. West, E. N. Atochina-Vasserman, L. C. Lindesmith, N. Pardi, R. Parks, M. Barr, D. Li, B. Yount, K. O. Saunders, D. Weissman, B. F. Haynes, S. A. Montgomery, R. S. Baric, Chimeric spike mRNA vaccines protect against Sarbecovirus challenge in mice. *Science* **373**, 991–998 (2021). doi:10.1126/science.abi4506 Medline

47. D. Pinto, M. M. Sauer, N. Czudnochowski, J. S. Low, M. A. Tortorici, M. P. Housley, J. Noack, A. C. Walls, J. E. Bowen, B. Guarino, L. E. Rosen, J. di Iulio, J. Jerak, H. Kaiser, S. Islam, S. Jaconi, N. Sprugasci, K. Culap, R. Abdelnabi, C. Foo, L. Coelmont, I. Bartha, S. Bianchi, C. Silacci-Fregni, J. Bassi, R. Marzi, E. Vetti, A. Cassotta, A. Ceschi, P. Ferrari, P. E. Cippà, O. Giannini, S. Ceruti, C. Garzoni, A. Riva, F. Benigni, E. Cameroni, L. Piccoli, M. S. Pizzuto, M. Smithey, D. Hong, A. Telenti, F. A. Lempp, J. Neyts, C. Havenar-Daughton, A. Lanzavecchia, F. Sallusto, G. Snell, H. W. Virgin, M. Beltramello, D. Corti, D. Veesler, Broad betacoronavirus neutralization by a stem helix-specific human antibody. *Science* **373**, 1109–1116 (2021). doi:10.1126/science.abj3321 Medline

48. M. M. Sauer, M. A. Tortorici, Y.-J. Park, A. C. Walls, L. Homad, O. J. Acton, J. E. Bowen, C. Wang, X. Xiong, W. de van der Schueren, J. Quispe, B. G. Hoffstrom, B.-J. Bosch, A. T. McGuire, D. Veesler, Structural basis for broad coronavirus neutralization. *Nat. Struct. Mol. Biol.* **28**, 478–486 (2021). doi:10.1038/s41594-021-00596-4 Medline

49. G. Song, W.-T. He, S. Callaghan, F. Anzanello, D. Huang, J. Ricketts, J. L. Torres, N. Beutler, L. Peng, S. Vargas, J. Cassell, M. Parren, L. Yang, C. Ignacio, D. M. Smith, J. E. Voss, D. Nemazee, A. B. Ward, T. Rogers, D. R. Burton, R. Andrabi, Cross-reactive serum and memory B-cell responses to spike protein in SARS-CoV-2 and endemic coronavirus infection. *Nat. Commun.* **12**, 2938 (2021). doi:10.1038/s41467-021-23074-3 Medline

50. P. Zhou, M. Yuan, G. Song, N. Beutler, N. Shaabani, D. Huang, W.-T. He, X. Zhu, S. Callaghan, P. Yong, F. Anzanello, L. Peng, J. Ricketts, M. Parren, E. Garcia, S. A. Rawlings, D. M. Smith, D. Nemazee, J. R. Teijaro, T. F. Rogers, I. A. Wilson, D. R. Burton, R. Andrabi, A protective broadly cross-reactive human antibody defines a conserved site of vulnerability on beta-coronavirus spikes. bioRxiv 2021.03.30.437769 [Preprint] (2021). https://doi.org/10.1101/2021.03.30.437769.

51. C. Wang, R. van Haperen, J. Gutiérrez-Álvarez, W. Li, N. M. A. Okba, I. Albulescu, I. Widjaja, B. van Dieren, R. Fernandez-Delgado, I. Sola, D. L. Hurdiss, O. Daramola, F. Grosveld, F. J. M. van Kuppeveld, B. L. Haagmans, L. Enjuanes, D. Drabek, B.-J. Bosch, A conserved immunogenic and vulnerable site on the coronavirus spike protein delineated by cross-reactive monoclonal antibodies. *Nat. Commun.* **12**, 1715 (2021). doi:10.1038/s41467-021-21968-w Medline

52. C.-L. Hsieh, A. P. Werner, S. R. Leist, L. J. Stevens, E. Falconer, J. A. Goldsmith, C.-W. Chou, O. M. Abiona, A. West, K. Westendorf, K. Muthuraman, E. J. Fritch, K. H. Dinnon 3rd, A. Schäfer, M. R. Denison, J. D. Chappell, R. S. Baric, B. S. Graham, K. S. Corbett, J. S. McLellan, Stabilized coronavirus spike stem elicits a broadly protective antibody. *Cell Rep.* **37**, 109929 (2021). doi:10.1016/j.celrep.2021.109929 Medline

53. A. L. Cathcart, C. Havenar-Daughton, F. A. Lempp, D. Ma, M. Schmid, M. L. Agostini, B. Guarino, J. Di iulio, L. Rosen, H. Tucker, J. Dillen, S. Subramanian, B. Sloan, S. Bianchi, J. Wojcechowskyj, J. Zhou, H. Kaiser, A. Chase, M. Montiel-Ruiz, N. Czudnochowski, E. Cameroni, S. Ledoux, C. Colas, L. Soriaga, A. Telenti, S. Hwang, G. Snell, H. W. Virgin, D. Corti, C. M. Hebner, The dual function monoclonal antibodies VIR-7831 and VIR-7832 demonstrate potent in vitro and in vivo activity against SARS-CoV-2. bioRxiv 2021.03.09.434607 [Preprint] (2021). https://doi.org/10.1101/2021.03.09.434607.

54. C. L. Hsieh, J. A. Goldsmith, J. M. Schaub, A. M. DiVenere, H. C. Kuo, K. Javanmardi, K. C. Le, D. Wrapp, A. G. Lee, Y. Liu, C. W. Chou, P. O. Byrne, C. K. Hjorth, N. V. Johnson, J. Ludes-Meyers, A. W. Nguyen, J. Park, N. Wang, D. Amengor, J. J. Lavinder, G. C. Ippolito, J. A. Maynard, I. J. Finkelstein, J. S. McLellan, Structure-based design of prefusion-stabilized SARS-CoV-2 spikes. *Science* **369**, 1501–1505 (2020). doi:10.1126/science.abd0826 Medline

55. D. Pinto, C. Fenwick, C. Caillat, C. Silacci, S. Guseva, F. Dehez, C. Chipot, S. Barbieri, A. Minola, D. Jarrossay, G. D. Tomaras, X. Shen, A. Riva, M. Tarkowski, O. Schwartz, T. Bruel, J. Dufloo, M. S. Seaman, D. C. Montefiori, A. Lanzavecchia, D. Corti, G. Pantaleo, W. Weissenhorn, Structural basis for broad HIV-1 neutralization by the MPER-specific human broadly neutralizing antibody LN01. *Cell Host Microbe* **26**, 623–637.e8 (2019). doi:10.1016/j.chom.2019.09.016 Medline

56. Y. Kaname, H. Tani, C. Kataoka, M. Shiokawa, S. Taguwa, T. Abe, K. Moriishi, T. Kinoshita, Y. Matsuura, Acquisition of complement resistance through incorporation of CD55/decay-accelerating factor into viral particles bearing baculovirus GP64. *J. Virol.* **84**, 3210–3219 (2010). doi:10.1128/JVI.02519-09 Medline

57. A. J. Greaney, T. N. Starr, P. Gilchuk, S. J. Zost, E. Binshtein, A. N. Loes, S. K. Hilton, J. Huddleston, R. Eguia, K. H. D. Crawford, A. S. Dingens, R. S. Nargi, R. E. Sutton, N. Suryadevara, P. W. Rothlauf, Z. Liu, S. P. J. Whelan, R. H. Carnahan, J. E. Crowe Jr., J. D. Bloom, Complete mapping of mutations to the SARS-CoV-2 spike receptor-binding domain that escape antibody recognition. *Cell Host Microbe* **29**, 44–57.e9 (2021). doi:10.1016/j.chom.2020.11.007 Medline

58. C. Suloway, J. Pulokas, D. Fellmann, A. Cheng, F. Guerra, J. Quispe, S. Stagg, C. S. Potter, B. Carragher, Automated molecular microscopy: The new Leginon system. *J. Struct. Biol.* **151**, 41–60 (2005). doi:10.1016/j.jsb.2005.03.010 Medline

59. D. Tegunov, P. Cramer, Real-time cryo-electron microscopy data preprocessing with Warp. *Nat. Methods* **16**, 1146–1152 (2019). doi:10.1038/s41592-019-0580-y Medline

60. A. Punjani, J. L. Rubinstein, D. J. Fleet, M. A. Brubaker, cryoSPARC: Algorithms for rapid unsupervised cryo-EM structure determination. *Nat. Methods* **14**, 290–296 (2017). doi:10.1038/nmeth.4169 Medline

61. J. Zivanov, T. Nakane, B. O. Forsberg, D. Kimanius, W. J. Hagen, E. Lindahl, S. H. Scheres, New tools for automated high-resolution cryo-EM structure determination in RELION-3. *eLife* **7**, e42166 (2018). doi:10.7554/eLife.42166 Medline

62. A. Punjani, H. Zhang, D. J. Fleet, Non-uniform refinement: Adaptive regularization improves single-particle cryo-EM reconstruction. *Nat. Methods* **17**, 1214–1221 (2020). doi:10.1038/s41592-020-00990-8 Medline

63. J. Zivanov, T. Nakane, S. H. W. Scheres, A Bayesian approach to beam-induced motion correction in cryo-EM single-particle analysis. *IUCrJ* **6**, 5–17 (2019). doi:10.1107/S205225251801463X Medline

64. S. Chen, G. McMullan, A. R. Faruqi, G. N. Murshudov, J. M. Short, S. H. Scheres, R. Henderson, High-resolution noise substitution to measure overfitting and validate resolution in 3D structure determination by single particle electron cryomicroscopy. *Ultramicroscopy* **135**, 24–35 (2013). doi:10.1016/j.ultramic.2013.06.004 Medline

65. P. B. Rosenthal, R. Henderson, Optimal determination of particle orientation, absolute hand, and contrast loss in single-particle electron cryomicroscopy. *J. Mol. Biol.* **333**, 721–745 (2003). doi:10.1016/j.jmb.2003.07.013 Medline

66. E. F. Pettersen, T. D. Goddard, C. C. Huang, G. S. Couch, D. M. Greenblatt, E. C. Meng, T. E. Ferrin, UCSF Chimera—A visualization system for exploratory research and analysis. *J. Comput. Chem.* **25**, 1605–1612 (2004). doi:10.1002/jcc.20084 Medline

67. P. Emsley, B. Lohkamp, W. G. Scott, K. Cowtan, Features and development of *Coot*. *Acta Crystallogr. D Biol. Crystallogr.* **66**, 486–501 (2010). doi:10.1107/S0907444910007493 Medline

68. B. Frenz, S. Rämisch, A. J. Borst, A. C. Walls, J. Adolf-Bryfogle, W. R. Schief, D. Veesler, F. DiMaio, Automatically fixing errors in glycoprotein structures with Rosetta. *Structure* **27**, 134–139.e3 (2019). doi:10.1016/j.str.2018.09.006 Medline

69. R. Y. Wang, Y. Song, B. A. Barad, Y. Cheng, J. S. Fraser, F. DiMaio, Automated structure refinement of macromolecular assemblies from cryo-EM maps using Rosetta. *eLife* **5**, e17219 (2016). doi:10.7554/eLife.17219 Medline

70. V. B. Chen, W. B. Arendall 3rd, J. J. Headd, D. A. Keedy, R. M. Immormino, G. J. Kapral, L. W. Murray, J. S. Richardson, D. C. Richardson, *MolProbity*: All-atom structure validation for macromolecular crystallography. *Acta Crystallogr. D Biol. Crystallogr.* **66**, 12–21 (2010). doi:10.1107/S0907444909042073 Medline

71. B. A. Barad, N. Echols, R. Y. Wang, Y. Cheng, F. DiMaio, P. D. Adams, J. S. Fraser, EMRinger: Side chain-directed model and map validation for 3D cryo-electron microscopy. *Nat. Methods* **12**, 943–946 (2015). doi:10.1038/nmeth.3541 Medline

72. D. Liebschner, P. V. Afonine, M. L. Baker, G. Bunkóczi, V. B. Chen, T. I. Croll, B. Hintze, L. W. Hung, S. Jain, A. J. McCoy, N. W. Moriarty, R. D. Oeffner, B. K. Poon, M. G. Prisant, R. J. Read, J. S. Richardson, D. C. Richardson, M. D. Sammito, O. V. Sobolev, D. H. Stockwell, T. C. Terwilliger, A. G. Urzhumtsev, L. L. Videau, C. J. Williams, P. D. Adams, Macromolecular structure determination using X-rays, neutrons and electrons: Recent developments in *Phenix*. *Acta Crystallogr. D Struct. Biol.* **75**, 861–877 (2019). doi:10.1107/S2059798319011471 Medline

73. J. Agirre, J. Iglesias-Fernández, C. Rovira, G. J. Davies, K. S. Wilson, K. D. Cowtan, Privateer: Software for the conformational validation of carbohydrate structures. *Nat. Struct. Mol. Biol.* **22**, 833–834 (2015). doi:10.1038/nsmb.3115 Medline

74. T. D. Goddard, C. C. Huang, E. C. Meng, E. F. Pettersen, G. S. Couch, J. H. Morris, T. E. Ferrin, UCSF ChimeraX: Meeting modern challenges in visualization and analysis. *Protein Sci.* **27**, 14–25 (2018). doi:10.1002/pro.3235 Medline

75. L. J. Reed, H. Muench, A simple method of estimating fifty per cent endpoints. *Am. J. Epidemiol.* **27**, 493–497 (1938). doi:10.1093/oxfordjournals.aje.a118408

76. K. H. D. Crawford, R. Eguia, A. S. Dingens, A. N. Loes, K. D. Malone, C. R. Wolf, H. Y. Chu, M. A. Tortorici, D. Veesler, M. Murphy, D. Pettie, N. P. King, A. B. Balazs, J. D. Bloom, Protocol and reagents for pseudotyping lentiviral particles with SARS-CoV-2 spike protein for neutralization assays. *Viruses* **12**, 513 (2020). doi:10.3390/v12050513 Medline
